# Novel Liver-targeted conjugates of Glycogen Phosphorylase Inhibitor PSN-357 for the Treatment of Diabetes: Design, Synthesis, Pharmacokinetic and Pharmacological Evaluations

**DOI:** 10.1038/srep42251

**Published:** 2017-02-22

**Authors:** Liying Zhang, Chengjun Song, Guangxin Miao, Lianzhi Zhao, Zhiwei Yan, Jing Li, Youde Wang

**Affiliations:** 1 Key Laboratory of Traditional Chinese Medicine Research and Development of Hebei Province, Institute of Traditional Chinese Medicine, Chengde Medical University, Chengde 067000, China; 2 Department of Human Anatomy, Chengde Medical University, Chengde 067000, China; 3 School of Bioscience and Bioengineering, South China University of Technology, Guangzhou 510006, China

## Abstract

**PSN-357**, an effective glycogen phosphorylase (GP) inhibitor for the treatment for type 2 diabetics, is hampered in its clinical use by the poor selectivity between the GP isoforms in liver and in skeletal muscle. In this study, by the introduction of cholic acid, 9 novel potent and liver-targeted conjugates of **PSN-357** were obtained. Among these conjugates, conjugate **6** exhibited slight GP inhibitory activity (IC_50_ = 31.17 μM), good cellular efficacy (IC_50_ = 13.39 μM) and suitable stability under various conditions. The distribution and pharmacokinetic studies revealed that conjugate **6** could redistribute from plasma to liver resulting in a considerable higher exposure of **PSN-357** metabolizing from **6** in liver (AUC_liver_/AUC_plasma_ ratio was 18.74) *vs* that of **PSN-357** (AUC_liver_/AUC_plasma_ ratio was 10.06). In the *in vivo* animal study of hypoglycemia under the same dose of 50 mg/kg, conjugate **6** exhibited a small but significant hypoglycemic effects in longer-acting manners, that the hypoglycemic effects of **6** is somewhat weaker than **PSN-357** from administration up to 6 h, and then became higher than **PSN-357** for the rest time of the test. Those results indicate that the liver-targeted glycogen phosphorylase inhibitor may hold utility in the treatment of type 2 diabetes.

Type 2 diabetes mellitus (T2DM) continue to expand at epidemic rates and new medicines targeting novel mechanisms are urgently needed. Glycogen phosphorylase (GP) is the key enzyme that catalyzes glycogenolysis, leading to the release of glucose from glycogen^1^. Three isoforms of GP have been identified that located within different metabolically active tissues for different physiological functions^2^. The muscle isoform provides energy for muscle contraction, the brain isoform provides an emergency supply of glucose during periods of anoxia or severe hypoglycemia, and the liver isoform regulates glucose release from hepatic glycogen stores^3^. In addition, it has been demonstrated that the inhibition of GP is involved in the promoting of glycogen synthesis in liver. Thus, GP, especially, the isoform in liver has received great recent interest as potential target for T2DM^4^. Although liver GP inhibition is regarded as an excellent therapeutic target for the treatment of diabetes, one very important factor relating to the relevance and importance of isoform specificity with this new therapeutic remains to be proven. As previously stated, brain, liver and skeletal muscle isoforms demonstrate 80% homology in their structures^5^, thus finding 100% specific inhibitors of the liver isoforms has proved difficult. Therefore, drug development programmes must consider the potential side effects of such compounds in relevant models. For example, inhibition of skeletal muscle GP when the liver isoform is the primary target could have devastating effects on maintaining muscle function. Since many type 2 diabetics are overweight and advised to increase their level of exercise as part of a treatment regimen, adverse effects on skeletal muscle function during exercise would severely limit the utility of a GP inhibitor^6^.

Effects of pharmacologic GP inhibition on skeletal muscle function after both acute and prolonged muscle contraction in a perfused rat hindlimb model have been reported. For example, the potent GP inhibitor **CP316819**, inhibition of GP during prolonged (60 min) muscle contraction resulted in a 35% greater muscle fatigue than the control group^7^,^8^. OSI Pharmaceuticals, Inc. have previously patented compound structures relating to pyrroloprridine-2-carboxylic acid amides and in early 2006 launched a Phase II clinical study of their promising GP inhibitor **PSN-357** for the treatment of diabetes and obesity, but development was discontinued for its side effects. The study was a combined single and multiple dose escalation program that is designed to determine the safety and tolerability of **PSN-357**, including appropriate exercise tolerance tests to evaluate the potential to compromise recommended positive life changes^9^,^10^.

Since the amino acid sequence homology among the GP isoforms is very high, the development of liver isoform-selective GP inhibitors may be a big challenge. And then, we thought about cholic acid, one of the “primary” bile acids formed from cholesterol in the liver, is tightly constrained within the enterohepatic loop through the action of a series of transporter proteins^11^. The highly efficient enteric and hepatic uptake mechanism ensures that less than 2% of cholic acid pool is excreted daily, even as it makes 10 or more passes through the intestine, portal vein, liver, and bile duct^12^. A cholic acid conjugate that retained these properties would be expected to have exceptionally substantial hepatic levels and a high liver-to-plasma ratio^13^. A series of works revealed that hybrid molecules formed by covalent linkage of a drug to cholic acid are recognized by the bile acid uptake systems in the liver and the ileum^14^,^15^,^16^. All the facts definitely indicate that cholic acid-drug conjugates should be considered as the solution to enhance the efficiency of liver-specific of **PSN-357**.

According to the previous primary structure-activity relationship of **PSN-357**, the hydroxyl of piperidine is not an essential group for the GP inhibitory activity that modification of it would not drastically decrease the hypoglycemic potency except for a few derivatives^17^. However, the molecular size of cholic acid is not so small as compared with **PSN-357**, it’s not hard to speculate that sterically hindrance might be occurred to interfering the interaction between **PSN-357** and GP when cholic acid is introduced to the hydroxyl directly, therefore, appropriate linkages such as amino acid, dipeptide or azo linkages were designed to providing enough space between **PSN-357** and cholic acid. 9 novel (**PSN-357**)**-**cholic acid conjugates were designed (Fig. 1) and synthesized, and the GP inhibition, hypoglycemic effects, biodistributionand pharmacokinetic studies were performed to evaluate the hypoglycemic activity and liver-targeting efficiency of the compounds. To the best of our knowledge, this is the first time to develop cholic acid-based conjugates as liver-specific derivatives of GP inhibitors.

## Results and Discussion

### Chemistry

**Figure 2:**
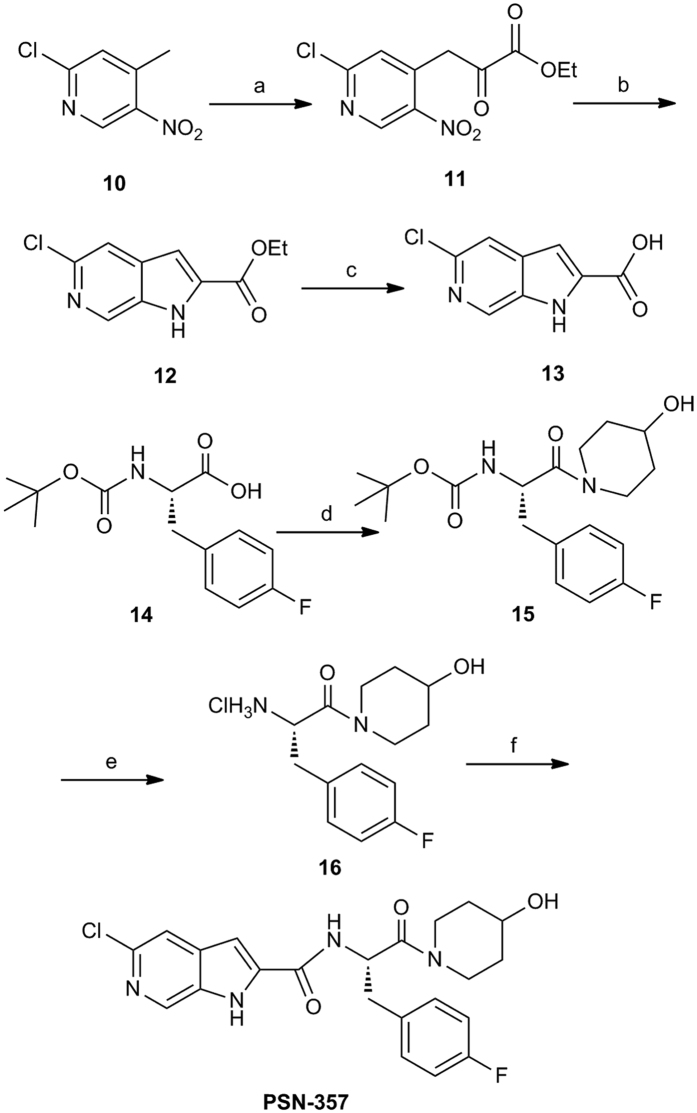
Synthesis of PSN-357^a^. Reagents and conditions: (**a**) Diethyl oxalate, C_2_H_5_OK, CH_3_OH/(C_2_H_5_)_2_O, rt; (**b**) Fe, NH_4_Cl, C_2_H_5_OH/THF, reflux, 32%; (**c**) NaOH, C_2_H_5_OH, reflux, 83%; (**d**) 4-hydroxypiperidine, HATU, DIPEA, anhydrous CH_2_Cl_2_, 0 °C to rt, 98%; (**e**) HCl/CH_2_Cl_2_, 0 °C to rt, 89%; (**f**) **13**, HATU, DIPEA, CH_2_Cl_2_, 0 °C to rt, 69%.

The GP inhibitor **PSN-357** was obtained according to the method previously described, with some modifications, as outlined in Fig. 2^18^. Commercially available 2-chloro-4-methyl-5-nitropyridine (**10**) was reacted with diethyl oxalate using potassium ethoxide as the base to give the corresponding derivative **11**. Derivative **11** was reduced with iron in saturated aqueous NH_4_Cl followed by cyclization to give pyrrolopyridine ester **12**. Hydrolyzed of **12** with NaOH afforded the carboxylic acid derivative **13** in excellent yield. On the other hand, for segment coupling, (*S*)-N-Boc-4-fluorophenylalanine (**14**) coupled to the 4-hydroxypiperidine in the presence of HATU and DIPEA to give **15** in 98% yield. Deprotection of the N-Boc group from **15** with HCl gave the amine hydrochloride salt **16**. Reaction of **16** with the carboxylic acid derivative **13** in the presence of HATU and DIPEA furnished **PSN-357** in 69% yield (Fig. 2).

**Figure 3:**
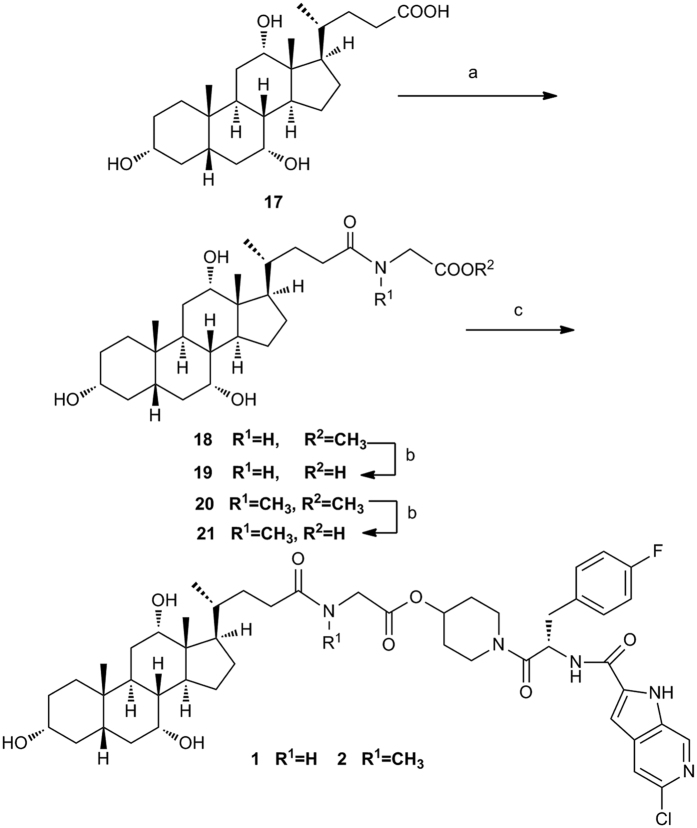
Synthesis of conjugates 1–2^a^. Reagents and conditions: (**a**) **R**^**1**^**NHCHCOOMe·HCl**, HATU, DIPEA, THF, 30 °C, 63% yield for **18**, 48.6% yield for **20**; (**b**) LiOH·H_2_O, THF/H_2_O, rt, 76% yield for **19**, 79% yield for **21**; (**c**) **PSN-357**, DCC, DMAP, DMF, rt, 10.9% yield for **1**, 21% yield for **2**.

The synthesis of cholate conjugate **1** and **2** are shown in Fig. 3. Reaction of cholic acid (**17**) with glycine methyl ester provided amide derivative **18** in 63% yield. Hydrolyzed of **18** with LiOH afforded the glycine conjugate **19** in 76% yield. Compound **19** was treated with **PSN-357** in the presence of DCC and DMAP to obtain conjugate **1**. In the same fashion, conjugate **2** was prepared from cholic acid and sarcosine methyl ester.

**Figure 4:**
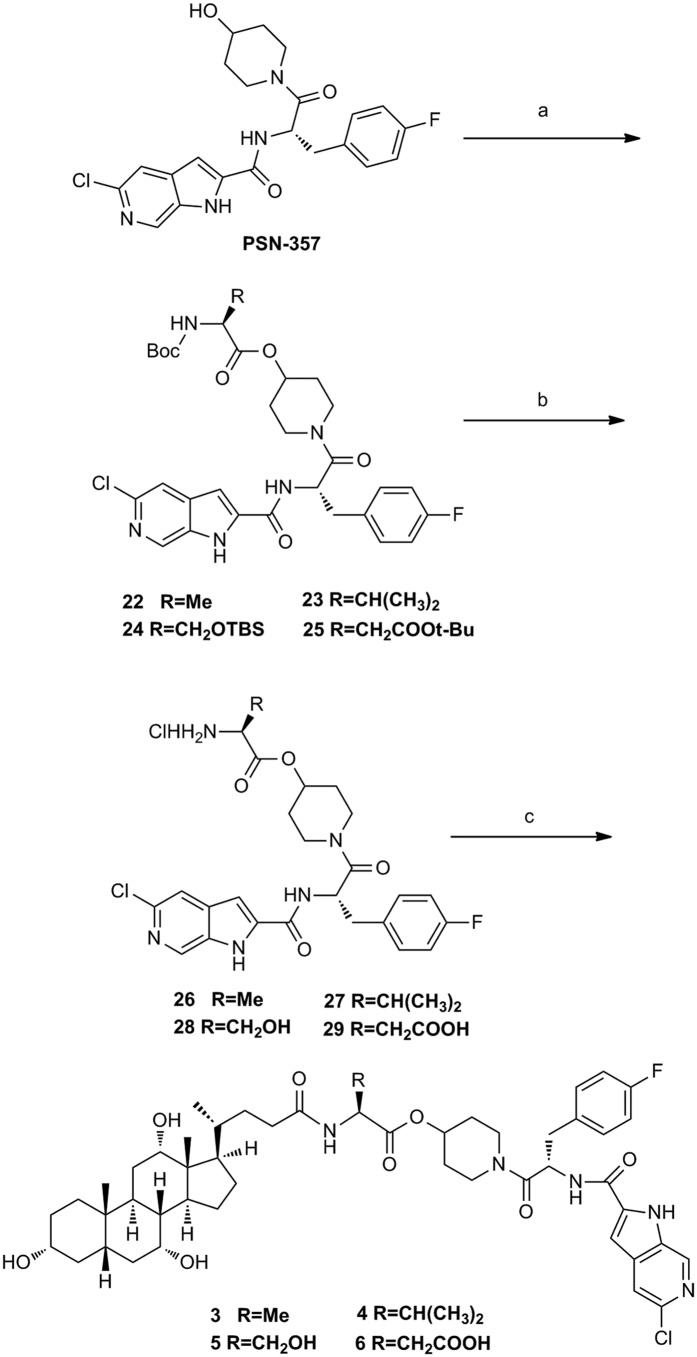
Synthesis of conjugates 3–6^a^. Reagents and conditions: (**a**) Boc-NH-CHR-COOH, DCC, DMAP, CH_2_Cl_2_, 0 °C to rt; (**b**) HCl/EtOAc, 0 °C to rt, 2 steps 40% yield for **26**, 2 steps 44% yield for **27**, 2 steps 70% yield for **28**, 67.2% yield for **29**; (**c**) Cholic acid, HATU, DIPEA, DMF, rt, 30.7% yield for **3**, 29.2% yield for **4**, 20% yield for **5**, 15.8% yield for **6**.

The synthesis of conjugates **3–6** was carried out starting from **PSN-357** (Fig. 4). Reaction of **PSN-357** with various protected amino acids (*N*-Boc-L-Ala-OH, *N*-Boc-L-Val-OH, N-Boc-O-TBS-L-Ser-OH, Boc-Asp(OtBu)-OH) produced intermediates **22–25**. Cleavage of the protecting groups in intermediates **22–25** afforded hydrochloride salts **26–29**. Direct coupling of **26–29** with cholic acid in the presence of HATU and DIPEA gave the expected conjugates **3–6**.

**Figure 5:**
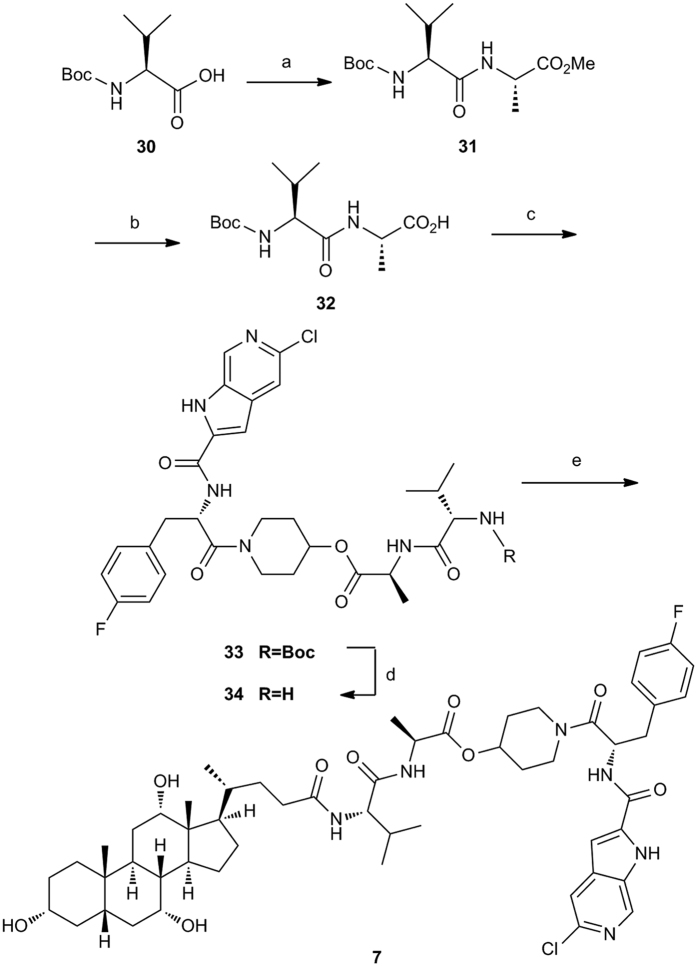
Synthesis of conjugate 7^a^. Reagents and conditions: (**a**) L-Alanine methyl ester hydrochloride, EDCI, HOBt, DIPEA, DMF, rt, 69.8%; (**b**) NaOH, MeOH/H_2_O, rt, 66.9%; (**c**) **PSN-357**, DCC, DMF, rt, 52%; (**d**) TFA, CH_2_Cl_2_, 0 °C; (**e**) Cholic acid, HATU, Et_3_N, DMF, rt, 2 steps 10.7% yield for **7**.

As shown in Fig. 5, the preparation of conjugate **7** via a multistep process by modifying the well-known synthesis method reported in the literature^19^. Coupling of *N*-Boc-L-Val-OH with L-alanine methyl ester hydrochloride yielded N-protected dipeptide ester **31**. Hydrolyzed of **31** with LiOH provided Boc-val-ala-OH **32**. The dipeptide derivative **32** was treated with **PSN-357** to obtain compound **33** in 52% yield. Subsequent deprotection of compound **33** using TFA gave compound **34**, which was reacted with cholic acid through the action of HATU and Et_3_N to give the conjugate **7**.

**Figure 6:**
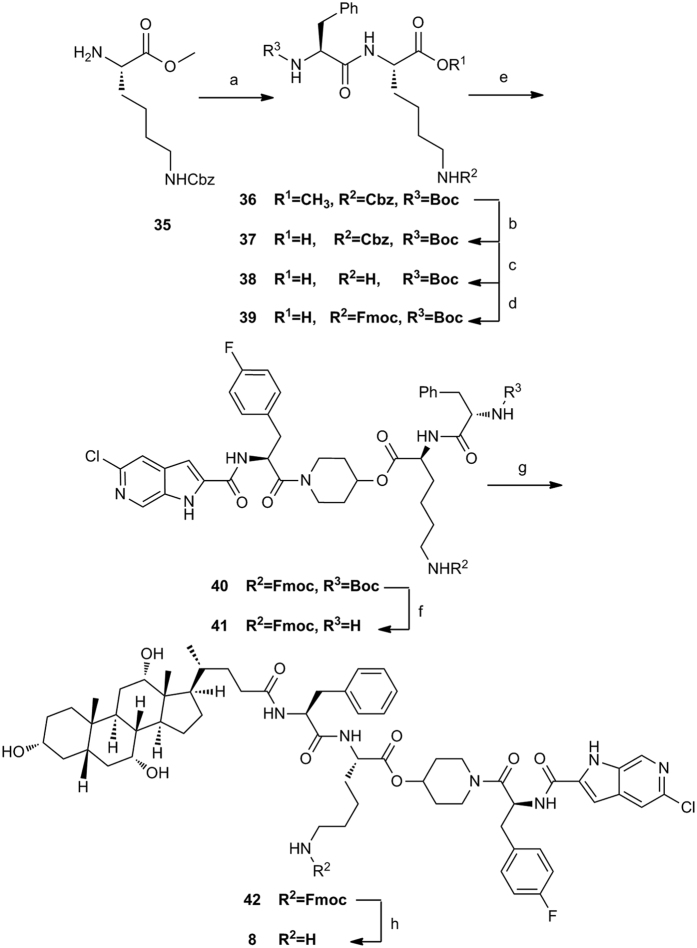
Synthesis of conjugate 8^a^. Reagents and conditions: (**a**) Boc-L-phenylalanine, EDCI, HOBt, DIPEA, DMF, rt, 73%; (**b**) NaOH, MeOH/H_2_O, rt, 79%; (**c**) H_2_, Pd/C, CH_3_OH, rt,; (**d**) FmocCl, NaHCO_3_, 1,4-dioxane, 0 °C; (**e**) **PSN-357**, T_3_P, CH_2_Cl_2_, rt, 3 steps 19.2% yield for **40**; (**f**) TFA, CH_2_Cl_2_, 0 °C; (**g**) Cholic acid, HATU, Et_3_N, DMF, rt, 2 steps 48.5% yield for **42**; (**h**) Piperidine, CH_3_CN, rt, 45%.

Conjugate **8** was obtained in a similar manner using dipeptide linkage prepared from the protected lysine ester (Fig. 6). Treatment of N6-Cbz-L-lysine methyl ester **35** with Boc-L-phenylalanine in the presence of EDCI in DMF at room temperature afforded dipeptide ester **36**. Hydrolyzed of **36** with NaOH in MeOH/H_2_O gave dipeptide derivative **37** in 79% yield. Hydrogenolysis of **37** over Pd/C in MeOH furnished **38**, followed by protection of the resulting amine with FmocCl produced dipeptide intermediate **39**. Conjugation of **39** with **PSN-357** in the presence of 1-propanephosphonic acid cyclic anhydride (T3P) gave the compound **40**. Removal of the protected group from **40** with TFA furnished intermediate **41**, which was directly reacted with cholic acid to give the intermediate **42** in a similar reaction pattern as conjugate **7**. Deprotection of the Fmoc group of **42** with piperidine afforded the target conjugate **8**.

**Figure 7:**
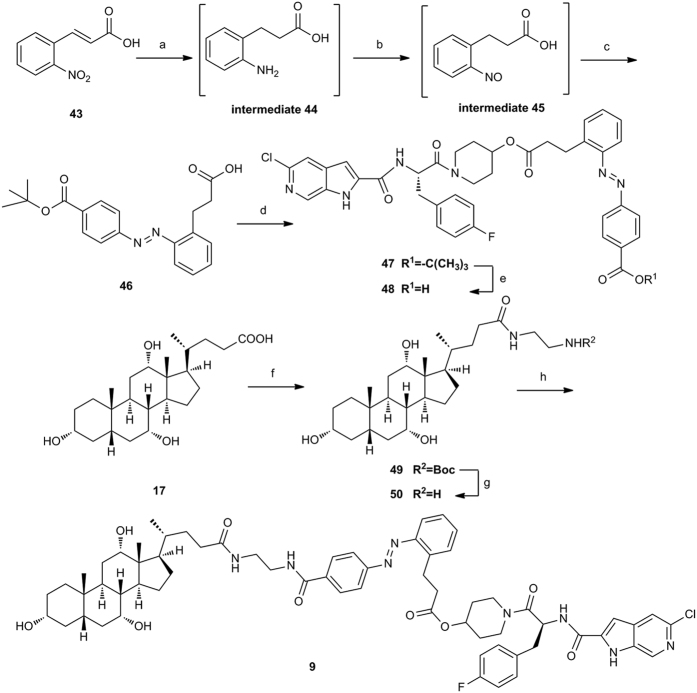
Synthesis of conjugate 9^a^. Reagents and conditions: (**a**) H_2_, Pd/C, NaOH, rt; (**b**) Oxone, H_2_O/CH_2_Cl_2_; rt; (**c**) tert-Butyl 4-aminobenzoate, AcOH, 80 °C, 3 steps 17.4% yield for **46**; (**d**) **PSN-357**, DCC, DMAP, CH_2_Cl_2_, rt, 82.9%; (**e**) TFA, CH_2_Cl_2_, rt, 79.5%; (**f**) N-Boc-ethylenediamine, DEPC, Et_3_N, DMF, 0 °C to rt, 74%; (**g**) HCl/MeOH, 0 °C to rt, 97%; (**h**) **48**, HATU, DIPEA, DMF, rt, 10.4%.

The complete synthesis of conjugate **9** containing an aromatic azo-linkage is depicted in Fig. 7. Reduction of *o*-nitrocinnamic acid **43** under H_2_ atmosphere with Pd/C in aqueous NaOH gave the amine intermediate **44**. It was necessary to perform the reduction under basic condition in order to inhibit cyclization of itself. Intermediate **44** was oxidized using Oxone to give nitroso acid intermediate **45**. The condensation between intermediate **45** and *tert*-butyl 4-aminobenzoate was performed in glacial acetic acid at room temperature to give the compound **46**. Treatment of **46** with **PSN-357** using the Steglich reaction afforded compound **47**. Deprotection of the *tert*-butyl group from **47** using TFA gave the intermediate carboxylic acid **48** in high yield. On the other hand, coupling of cholic acid with N-Boc-ethylenediamine in the presence of DEPC and Et_3_N in DMF gave compound **49** in 74% yield. Deprotection of the Boc group was performed with 6 M methanolic HCl to yield the cholic acid derivative **50**. Compound **50** was attached to the corresponding carboxylic acid **48** under amide-bond-forming condition in the presence of HATU to give the conjugate **9**.

### Enzyme Assay and SAR Analysis

All the compounds were evaluated for their inhibitory activity against rabbit muscle GPa (RMGPa). The activity of RMGPa was measured through detecting the release of phosphate from glucose-1-phosphate in the direction of glycogen synthesis^20^. **CP-91149**, a well-known allosteric GP inhibitor, which shares the same binding site with **PSN-357**, was used as the positive control and the results are summarized in Table 1^21^. The assay results showed that most of the synthesized compounds exhibited moderate to good inhibitory activities against RMGPa. Compared to **PSN-357** (IC_50_ = 0.42 μM), introduction of cholic acid group to **PSN-357** resulted in a great loss of activity (**1–3**, **5–9**) or no activity (**4**). This remarkable loss of activity was probably due to the direct steric interference by such bulky substituent of cholic acid group. Among the compounds, conjugate **1** (IC_50_ = 5.9 μM) displayed the most efficient inhibition. While the GP inhibition of the intermediates (e.g. **25**–**28, 33, 40, 42, 48**) seemed much acceptable, indicating that introduction of different substitutions in small size on the hydroxyl moiety of **PSN-357** resulted in a slight loss of potency. The results is consistent with the SARs of **PSN-357** that the hydroxyl of piperidine is not an essential group for the GP inhibitory activity that could be modified slightly^9^. In general, the L-serine derivative **28** (IC_50_ = 0.52 μM) was the most active of the series, being approximately 1.25-fold less potent than that of **PSN-357**.

### Cell Assay and SAR Analysis

To evaluate the effects of all compounds in cells, the glycogenolysis assays were established in both rat and human liver cells based on the published method^20^. These results are summarized in Table 2. Two of the conjugates revealed excellent inhibitory activity in the cellular assays. Of these, conjugate **9** showed the best activity in isolated rat hepatocytes and HepG2 cells, with IC_50_ value of 12.3 μM and 6.4 μM, respectively. Likewise, conjugate **6** showed IC_50_ value of 13.4 μM in isolated rat hepatocytes and 6.1 μM in HepG2 cells, respectively. The inhibitory activities of the derivatives of **PSN-357** (e.g. **25**–**28**) were also explored. It is not surprising that the derivatives still retained micromolar inhibitory activities. Introduction of steric bulks to the hydroxyl group (e.g. **33, 40, 42, 48**) led to a complete loss of activity. Data analysis indicated no clear SAR for the substitutents in the cell-based assays.

### *In Vitro* Stability studies

All the conjugates were tested for their chemical and metabolic stability in multiple assays, including simulated gastric fluid (SGF), simulated interstinal fluid (SIF), mouse plasma, and mouse liver microsome (MLM).

**Figure 8:**
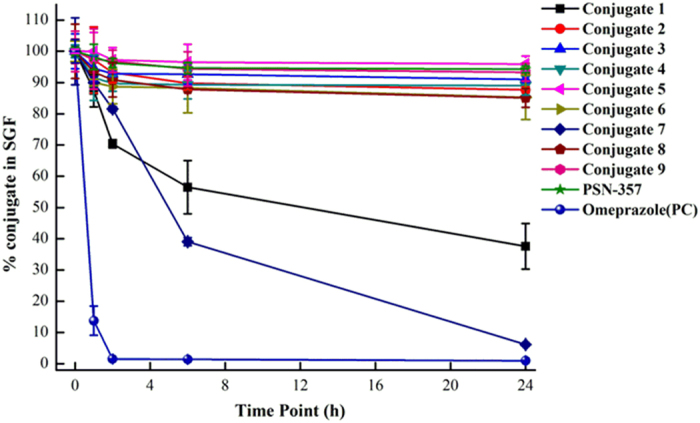
Time-course of SGF stability for conjugates 1–9 (n = 3).

**Figure 9:**
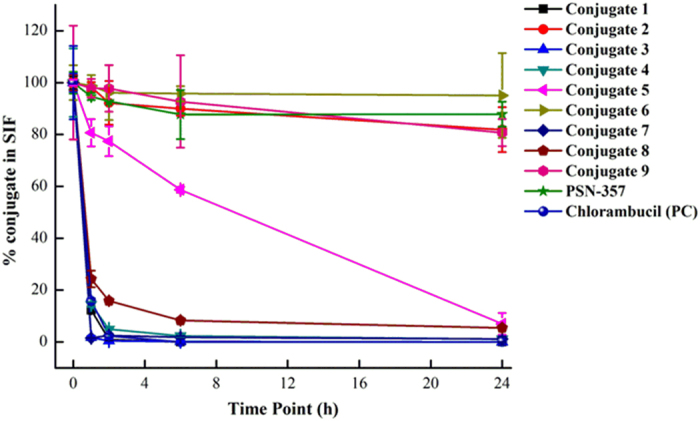
Time-course of SIF stability for conjugates 1–9 (n = 3).

For the stability in SGF (Fig. 8) and SIF (Fig. 9), the conjugates could be divided into three groups: (1) stable conjugates **2**, **6** and **9**. All of them were stable in SGF for 24 h with almost no detectable **PSN-357**, but in SIF, conjugate **6**, being stable up to 24 h, were much more stable than **2** and **9**. Still, **2** and **9** were relatively stable than other conjugates that no more than 20% of the two compounds were degraded during the 24 h incubation in SIF; (2) unstable conjugates **1** and **7**. The two conjugates were degraded within 6 h and 1 h in SGF and SIF, respectively, with alomost no conjugates at the end of the 24 h incubation in both SGF and SIF; (3) complicated conjugates **3**, **4**, **5** and **8**. They were relatively stable in SGF over 24 h of incubation, but degraded rapidly in SIF within 1 h.

**Figure 10:**
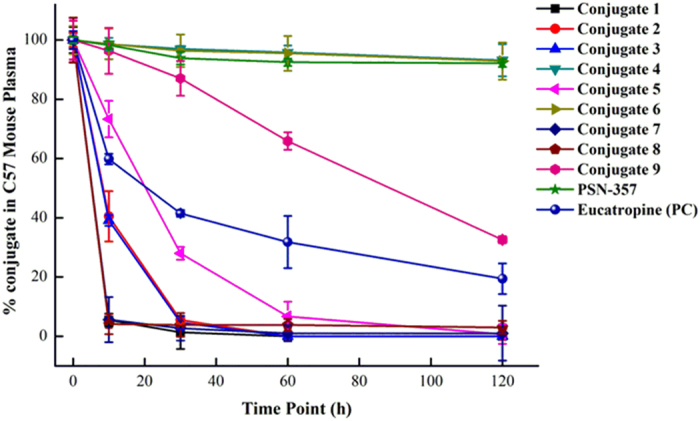
Time-course of mouse plasma stability for conjugates 1–9 (n = 3).

As shown in Fig. 10, **PSN-357** was relatively stable in mouse plasma within the 120-min test, while the conjugates degraded and declined in a mono-exponential model, except conjugates **4** and **6**. Conjugates **4** and **6** exhibited considerable stablity in mouse plasma as **PSN-357** with approximately 4% and 12% degradation, respectively.

Additionally, the compounds’ stability in microsome were evaluated by measuring the rate of compounds consumpation in MLM and the results are shown in Table 3. **PSN-357** demonstrated good metabolic stability in MLM with longer half life (t_1/2_ > 145 min) and slower elimination rate (CL_int_ < 9.6 μL/min/mg protein, CL < 38.0 μL/min/mg protein). Conjugates **6**, **8** and **9** showed considerable metabolic stability with t_1/2_ over a range of 84.5 to 91.2 min and CL_int_ over a range of 15.2 to 16.4 μL/min/mg protein. Conjugates **1–5**, exhibited poor metabolic stability resulting in a short elimination half-life and a high systemic clearance relative to conjugates **6**, **8** and **9**. In addition, conjugate **7**, it metabolized fastest in MLM, with a t_1/2_ of 4.7 min and displayed very high intrinsic hepatic clearance (CL_int_ of 297.4 μl**/**min/mg), suggesting the potential for unacceptably high hepatic clearance. Those results indicate that the release of **PSN-357** from the conjugates were greatly affecting by the linkers.

### Biodistribution and Pharmacokinetic Studies for Compound PSN-357 and Conjugate 6 in Mice

**Figure 11:**
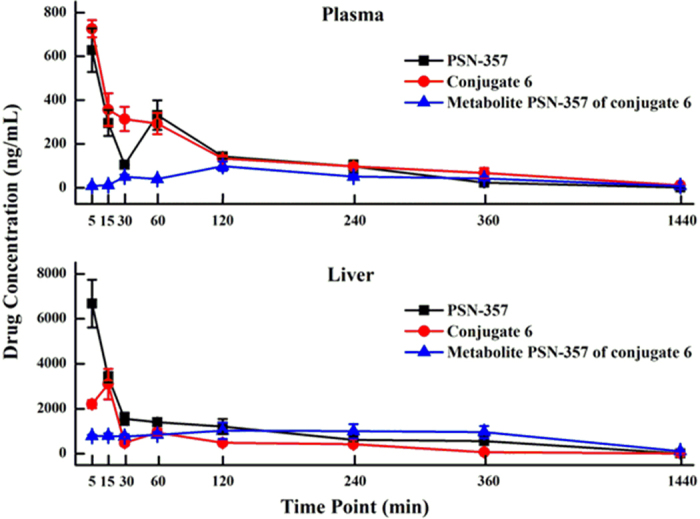
The drug concentration-time curve in the plasma and liver (ng/mL) after intravenous administration of PSN-357 and conjugate 6 in mice (n = 3).

Based on the results of the potency and *in vitro* stability studies, conjugate **6** was selected for *in vivo* pharmacokinetic analysis following a single intravenous injection of 5 mg/kg in male C57 BL/6. Doses of **PSN-357** intravenous were used as standard regimens for comparison. The results are shown in Table 4 and Fig. 11. In plasma, the concentration of **PSN-357** reached the C_max_ of 628 ng/mL at 5 min and then sharply decreased during 30 min after dosing, but it gradually increased to reach a secondary peak at 60 min perhaps due to enterohepatic circulation, and the value of AUC_0-t_ is 965 ng/mL.h. For conjugate **6**, the concentration reached the C_max_ of 726 ng/mL at 5 min and then rapidly decreased to 97 ng/mL at 240 min after dosing. In addition, the concentration of major metabolite **PSN-357** released from **6** reached the C_max_ of 98 ng/mL at 120 min with the AUC_0-t_ of 691.67 ng/mL.h, about 6 and 1.4-fold lower than that of **PSN-357**, respectively. On the other hand, in livers, conjugate **6** did not show the highest concentration at 5 min after administration as compared with **PSN-357**. The concentration of **6** is dramatically increased to reaching a mean C_max_ of 3094.55 ng/mL at about 15 min after dosing, suggesting the redistribution from plasma or other tissues to livers may have happened, then **6** was eliminated during 30–240 min. For **PSN-357** released from conjugate **6**, the value of C_max_ is 1023 ng/mL, about 6-fold lower than that of **PSN-357**, but the value of AUC_0-t_ is 12960 ng/mL.h, about 1.4-fold higher than that of **PSN-357** (9711 ng/mL.h), further, (AUC_liver_/AUC_plasma_) of **PSN-357** metaboliting from **6** is 18.74, about 2-fold higher than that of **PSN-357** (10.06). Those results suggested that conjugate **6** exhibited some targeting effect to liver that might enrich and display a longer duration of action in liver in comparison with **PSN-357**.

### *In Vivo* Efficacy of Compound PSN-357 and Conjugate 6

**Figure 12:**
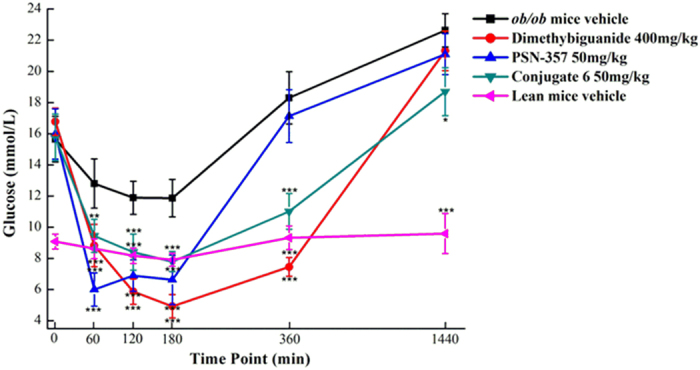
Time-course of glucose lowering following oral administration of PSN-357 and conjugate 6 in diabetic *ob/ob* mice. (n = 10; *p < 0.05, **p < 0.01 and ***p < 0.005 vs vehicle-treated *ob/ob* mice).

*In vivo* efficacy of compound **PSN-357** and conjugate **6** were studied on leptin-deficient *ob*/*ob* mice. Metformin was chosen as a positive control. Figure 12 showed that treatment with **PSN-357** (50 mg/kg) could significantly reduc the blood glucose (BG) to a nadir of 6.36 ± 1.46 mg/dl at 1 h min vs 12.80 ± 1.58 in control *ob/ob* mice (p < 0.005), with significant effects also being evident at 2 h (p < 0.005) and 3 h (p < 0.005) vs controls. A similar but somewhat weaker hypoglycemic effect was observed for the conjugate **6** under the same dosage. Glucose lowering was statistically significant at 1 (p < 0.01), 2 (p < 0.005), 3 (p < 0.005), 6 (p < 0.005) and 24 h (p < 0.05), with the largest drop (BG level is around 7.79 mmol/L) occurring at 3 h. Especially, there was still significant decrease in BG levels by conjugate **6** up to 6 h after administration. The effect might be due to the fact that **6** acts in a sustained release and longer acting manners. It is noteworthy that the findings are highly consistent with the results from *in vivo* pharmacokinetic studies.

## Conclusions

In summary, though a strategy of bile acid conjugation, it has been found possible to prepare liver-selective conjugates of **PSN-357**. The *in vitro* biological and stability studies of these conjugates were evaluated to supporting the selection of a conjugate candidate for *in vivo* pharmacokinetics and pharmacological evaluation. Among the conjugates, conjugate **6** exhibited moderate enzyme inhibitory activity, suitable cellular activity and acceptable stability in various biological fluids. This compound is preferentially distributed into liver and possesses a longer duration of action than **PSN-357** at the same dose. Moreover, conjugate **6** was able to maintain acceptable antidiabetic effects relative to **PSN-357**. These results implied that the development of liver-selective conjugates might offer a potential opportunity to overcome the muscles side-effects caused by sequence homology of three GP isoforms.

## Materials and General Methods

### Chemistry section

(The detailed information is in Supplementary information).

### Enzyme Kinetics

The inhibitory activity of the test compounds against rabbit muscle glycogen phosphorylase a (GPa) was monitored using microplate reader (BIO-RAD) based on the published method^20^. In brief, GPa activity was measured in the direction of glycogen synthesis by the release of phosphate from glucose-1-phosphate. Each test compound was dissolved in DMSO and diluted at different concentrations for IC_50_ determination. The enzyme was added into 100 μL of buffer containing 50 mM Hepes (pH = 7.2), 100 mM KCl, 2.5 mM MgCl_2_, 0.5 mM glucose-1-phosphate, 1 mg/mL glycogen and the test compound in 96-well microplates (Costar). After the addition of 150 μL of 1 M HCl containing 10 mg/mL ammonium molybdate and 0.38 mg/mL malachite green, reactions were run at 22 °C for 25 min, and then the phosphate absorbance was measured at 655 nm. The IC_50_ values were estimated by fitting the inhibition data to a dose-dependent curve using a logistic derivative equation.

### Glycogenolysis Inhibition in Rat Hepatocytes and HepG2 cells

The inhibition of hepatic glycogenolysis was monitored by the measurement of liver glycogen, which was done quantitatively by the anthrone reagent (Sigma) colorimetric method based on the published method^20^. Isolated rat hepatocytes or HepG2 cells (Sigma) were treated with the test compound or DMSO solvent (final concentration, 0.10%), followed by 60-min incubation with 0.3 nM glucagon (GGN). Assays were terminated by centrifugation, and cells were digested with 30% KOH followed by glycogen determination. The IC_50_ values were estimated by fitting the inhibition data to a dose-dependent curve using a logistic derivative equation.

### Stability Tests of the Conjugates

#### Stability in Simulated Gastrointestinal Fluids

The simulated gastrointestinal fluids were prepared according to USP specifications. For SGF, NaCl (0.12 g) and pepsin (0.18 g, from porcine stomach mucosa) was dissolved in HCl (0.42 mL) and sufficient H_2_O was added to make 60 mL. The pH of the test solution was determined as 1.21 by pH meter. For the SIF, KH_2_PO_4_ (0.408 g) and pancreatin (0.6 g, from porcine pancreas) was dissolved in H_2_O (60 mL). The pH of the test solution was determined as 6.79 by pH meter. Each test compound was dissolved in DMSO and diluted to a final concentration of 2 μM in SIF and SGF. The final concentrations of DMSO and CH_3_CN in the incubation mixture were 0.2% and 0.45%. Aliquots of these solutions were pipetted into glass tubes, and placed in a 37 °C shaking water bath. Test samples at corresponding time point (1, 2, 6, 24 h) were removed at the end of incubation time and immediately mixed with 800 μL of cold acetonitrile containing 500 ng/mL tolbutamide (internal standard). Samples were subjected to centrifuge at 4000 rpm, 4 °C for 20 min. Aliquots 60 μL of supernatant was combined with 120 μL water for LC-MS/MS analysis.

#### Stability in Plasma

Frozen plasma from male C57 BL/6 mice was incubated for 5 min at 37 °C before the addition of the test compound. Then prepared 2 μM incubation sample, and aliquots of the incubation mixtures (100 μL) were taken at predetermined time points (10, 30, 60, 120 min) at 37 °C. After incubation, the mixtures was added 400 μL of 50% ACN/50% MeOH containing internal standard (IS, 200 ng/mL Tolbutamide and 20 ng/mL Buspirone) to each sample tube and then centrifuged at 13000 rpm for 8 min. Blank incubations in the absence of the test compound were also performed. The analyses of test compounds were performed by LC-MS/MS analysis.

#### Stability in Liver Microsomes

The microsomal pellet was suspended in potassium phosphate buffer (100 mM, pH 7.4), 10 μM test compound or positive control (diclofenac) was added. Liver microsomes from C57BL/6 mice (BD Gentest) were pooled. The mixture of microsome solution and compound was incubated at 37 °C for about 10 min in the presence of a NADPH-regenerating system, consisting of 0.02 M DL-isocitric acid (trisodium salt), 0.1 mg/mL isocitrate dehydrogenase and 1 mM NADPH. The addition of ice-cold CH_3_CN (including 100 ng/mL tolbutamide as internal standard) terminated the reaction. The mixture was vortexed for approx (30 s), centrifuged (20 min, 4000 rpm) and the supernatant was collected and analysed by LC-MS/MS method.

### Tissue Distribution and Pharmacokinetic Parameters for Compound PSN-357 and Conjugate 6

The *in vivo* experiments of compound **PSN-357** and conjugate **6** were determined as described below. The animals were housed and cared for in accordance with the guidelines established by the National Science Council of Republic China. All experimental protocols were approved by Animal Care and Use Committee of Chengde Medical University, and all experiments were performed in accordance with the approved guidelines.

Male C57 BL/6 mice (18–20 g, 5–6 weeks old) were provided by the Laboratory Animal Center of Academy of Military Medical Science of People’s Liberation Army, and were reared in standard rodent cages in animal house of Chengde Medical University, under the condition of a constant temperature of 24 °C (±2 °C) and a 12 h light/dark cycle. **PSN-357** and conjugate **6** were injected through the caudal vein. For each sampling time point, three mice were treated with a single dose of either **PSN-357** or conjugate **6** at 5 mg/kg in 10% DMSO/saline solutions. Blood and liver samples were taken at 0.0833, 0.25, 0.5, 1, 2, 4, 6 and 24 h following intravenous administration, and stored at −80 °C until analysis. Aliquots of all biological matrixes were deproteinized with a mixture of methanol/acetonitrile (1:1). The suspension was vortexed, mixed, and centrifuged at 13000 rpm for 10 min. The organic phase was injected into the LC-MS/MS system. PK calculations and statistical comparisons on PK data were performed according to a non-compartmental kinetic model with validated software (Kinetica™ version 5.1, Thermo Electron Corporation, USA).

### Glucose Lowering *in Vivo*

Five- to six-week old obese, diabetic *ob*/*ob* mice (male C57BL/6 J) and their lean, nondiabetic male C57BL/6 J littermates were provided by the Model Animal Research Center of Nanjing University, and housed under standard animal care practices with *ad libitum* access to food and water throughout the procedures. After 1 week acclimation, blood was collected from the retroorbital sinus for plasma glucose determination, and mice were randomized to groups with similar mean ± SD. Mice were then dosed p.o. daily for 4 days with vehicle consisting of DMSO/PEG400/H-β-CD (1:3:6, v/v/v). On day 5, mice were treated p.o. with **PSN-357** (50 mg/kg) or conjugate **6** (50 mg/kg) or vehicle, then bled at 0, 1, 2, 3, 6 and 24 h post-dose for plasma glucose determination. Statistical analysis of the hypoglycemic effect was determined by unpaired t test with the ob/ob mice vehicle treated group.

## Additional Information

**How to cite this article**: Zhang, L. *et al*. Novel Liver-targeted conjugates of Glycogen Phosphorylase Inhibitor PSN-357 for the Treatment of Diabetes: Design, Synthesis, Pharmacokinetic and Pharmacological Evaluations. *Sci. Rep.* **7**, 42251; doi: 10.1038/srep42251 (2017).

**Publisher's note:** Springer Nature remains neutral with regard to jurisdictional claims in published maps and institutional affiliations.

## Supplementary Material

Supplementary Information

## Figures and Tables

**Figure 1:**
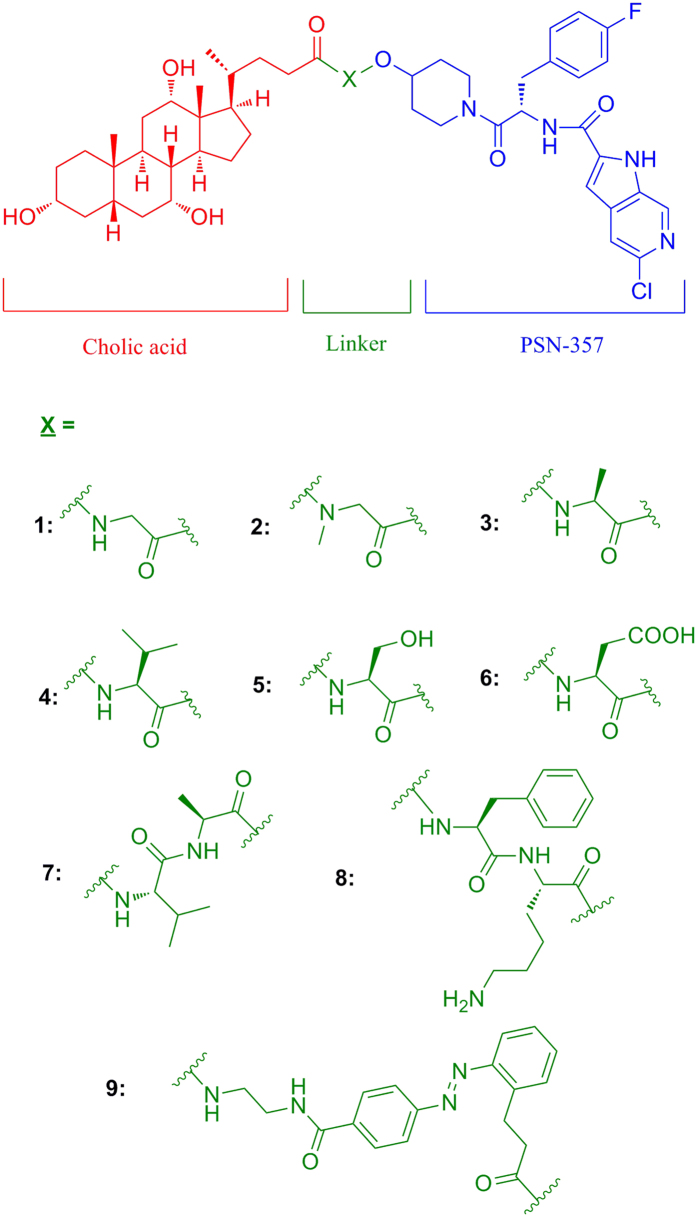
Chemical Structures of 9 novel cholic acid conjugates of **PSN-357**.

**Table 1:** RMGPa inhibition assay for conjugates **1**–**9** and some intermediates.

Compound	IC_50_^a^ (μM)	Compound	IC_50_ (μM)
1	5.94 ± 0.74	**27**	0.88 ± 0.17
2	26.01 ± 13.9	**28**	0.52 ± 0.01
3	40.53 ± 15.7	**33**	27.03 ± 6.39
4	NI^b^	**40**	3.44 ± 0.22
5	81.94 ± 14.4	**42**	15.42 ± 0.89
6	31.17 ± 11.1	**47**	NI
7	6.07 ± 0.27	**48**	3.11 ± 0.05
8	10.74 ± 5.6	**49**	NI
9	51.69 ± 4.1	**50**	NI
17	NI	**PSN-357**	0.42 ± 0.01
25	0.81 ± 0.077	**CP-91149**^c^	0.09 ± 0.04
26	0.56 ± 0.043		

^a^Each value represents the mean ± S.D. of three determinations.

^b^NI means no inhibition.

^c^CP-91149 was used as positive control.

**Table 2:** Glycogenolysis inhibition assay for conjugates **1**–**9** and some intermediates in liver cells.

Compound	IC_50_^a^ (μM, rat hepatocytes)	IC_50_^a^ (μM, HepG2 cells)	Compound	IC_50_^a^ (μM, rat hepatocytes)	IC_50_^a^ (μM, HepG2 cells)
1	41.09 ± 5.11	203.27 ± 3.39	27	14.67 ± 4.99	16.76 ± 3.71
2	845.35 ± 132.30	171.61 ± 18.22	28	13.60 ± 0.23	2.88 ± 0.16
3	631.67 ± 99.43	NI^b^	33	11.09 ± 3.67	NI
4	NI	NI	40	38.42 ± 5.76	33.65 ± 12.17
5	NI	NI	42	5.56 ± 2.41	179.68 ± 51.21
6	13.40 ± 2.55	6.12 ± 1.38	47	97.58 ± 1.10	NI
7	42.08 ± 10.87	11.73 ± 3.80	48	35.02 ± 3.46	9.18 ± 7.42
8	64.54 ± 4.40	36.76 ± 4.14	49	NI	NI
9	12.29 ± 3.93	6.41 ± 1.25	50	NI	NI
25	10.20 ± 1.13	15.10 ± 4.11	PSN-357	10.73 ± 2.73	3.10 ± 0.37
26	9.79 ± 9.65	6.99 ± 2.10	CP-91149^c^	3.08 ± 1.16	2.53 ± 0.78

^a^Each value represents the mean ± S.D. of three determinations.

^b^NI means no inhibition.

^c^CP-91149 was used as positive control.

**Table 3:** *In vitro* T_1/2_ data and intrinsic clearance values for conjugates **1**–**9** in mouse liver microsomes (n = 3).

Compound	Mouse liver microsomes		
	t_1/2_ (min)	CL_int_ (μL/min/mg)	CL (mL/min/kg)
1	31.9	43.4	171.9
2	24.9	55.6	220.2
3	27.4	50.6	200.4
4	36.5	38.0	150.5
5	40.8	34.0	134.6
6	84.5	16.4	64.9
7	4.7	297.4	1177.7
8	85.6	16.2	64.2
9	91.2	15.2	60.2
PSN-357	>145	<9.6	<38.0
Diclofenac (PC)	26.3	52.6	208.3

**Table 4:** Pharmacokinetic parameters in plasma and liver tissue of **PSN-357** and conjugate **6** by 5 mg/kg intravenous administration in mice.

Tissue	PSN-357			Conjugate 6			Metabolite PSN-357 of conjugate 6		
	AUC_0-t_ (ng/mL·h)	C_max_ (ng/mL)	MRT_0-t_ (h)	AUC_0-t_ (ng/mL·h)	C_max_ (ng/mL)	MRT_0-t_ (h)	AUC_0-t_ (ng/mL·h)	C_max_ (ng/mL)	MRT_0-t_ (h)
Plasma	965.16	628.35	3.18	1518.40	726.2	7.52	691.67	98.33	10.55
Liver	9711.94	6677.85	4.52	3981.99	3094.55	4.42	12960.21	1023.14	9.97
