# Supplementary material for: Novel Liver-targeted conjugates of Glycogen Phosphorylase Inhibitor PSN-357 for the Treatment of Diabetes: Design, Synthesis, Pharmacokinetic and Pharmacological Evaluations
Source: Sci Rep. 2017 Feb 22;7:42251. doi: 10.1038/srep42251 (PMC5320518; doi:10.1038/srep42251)
Supplement: Supplementary Information [file srep42251-s1.doc]

**Supplementary Information**

**Novel Liver-targeted conjugates of Glycogen Phosphorylase Inhibitor PSN-357 for the Treatment of Diabetes: Design, Synthesis, Pharmacokinetic and Pharmacological Evaluations**

Liying Zhang,, Chengjun Song, Guangxin Miao, Lianzhi Zhao, Zhiwei Yan, Jing Li, Youde Wang.

**Contents**

[Detailed reaction procedures of all the compounds](#__RefHeading___Toc313788047) 5

[Copy of ^1^H NMR of 12](#__RefHeading___Toc313788047) 32

[Copy of ^13^C NMR of 12](#__RefHeading___Toc313788048) 33

[Copy of ^1^H NMR of 13](#__RefHeading___Toc313788049) 34

[Copy of ^13^C NMR of 13](#__RefHeading___Toc313788050) 35

[Copy of ^1^H NMR of 15](#__RefHeading___Toc313788051) 36

[Copy of ^13^C NMR of 15](#__RefHeading___Toc313788052) 37

[Copy of ^1^H NMR of 16](#__RefHeading___Toc313788053) 38

[Copy of ^13^C NMR of 16](#__RefHeading___Toc313788054) 39

[Copy of ^1^H NMR of PSN-357](#__RefHeading___Toc313788055) 40

[Copy of ^13^C NMR of PSN-357](#__RefHeading___Toc313788056) 41

[Copy of ^1^H NMR of 18](#__RefHeading___Toc313788057) 42

[Copy of ^13^C NMR of 18](#__RefHeading___Toc313788058) 43

[Copy of ^1^H NMR of 19](#__RefHeading___Toc313788060) 44

[Copy of ^13^C NMR of 19](#__RefHeading___Toc313788059) 45

[Copy of ^1^H NMR of 1](#__RefHeading___Toc313788062) 46

[Copy of ^13^C NMR of 1](#__RefHeading___Toc313788061) 47

[Copy of ^1^H NMR of 20](#__RefHeading___Toc313788064) 48

[Copy of ^13^C NMR of 20](#__RefHeading___Toc313788063) 49

[Copy of ^1^H NMR of 21](#__RefHeading___Toc313788066) 50

[Copy of ^13^C NMR of 21](#__RefHeading___Toc313788065) 51

[Copy of ^1^H NMR of 2](#__RefHeading___Toc313788068) 52

[Copy of ^13^C NMR of 2](#__RefHeading___Toc313788067) 53

[Copy of ^1^H NMR of 23](#__RefHeading___Toc313788070) 54

[Copy of ^13^C NMR of 23](#__RefHeading___Toc313788069) 55

[Copy of ^1^H NMR of 3](#__RefHeading___Toc313788072) 56

[Copy of ^13^C NMR of 3](#__RefHeading___Toc313788071) 57

[Copy of ^1^H NMR of 25](#__RefHeading___Toc313788074) 58

[Copy of ^13^C NMR of 25](#__RefHeading___Toc313788073) 59

[Copy of ^1^H NMR of 4](#__RefHeading___Toc313788076) 60

[Copy of ^13^C NMR of 4](#__RefHeading___Toc313788075) 61

[Copy of ^1^H NMR of 27](#__RefHeading___Toc313788078) 62

[Copy of ^13^C NMR of 27](#__RefHeading___Toc313788077) 63

[Copy of ^1^H NMR of 5](#__RefHeading___Toc313788080) 64

[Copy of ^13^C NMR of 5](#__RefHeading___Toc313788079) 65

[Copy of ^1^H NMR of 28](#__RefHeading___Toc313788082) 66

[Copy of ^13^C NMR of 28](#__RefHeading___Toc313788081) 67

[Copy of ^1^H NMR of 6](#__RefHeading___Toc313788084) 68

[Copy of ^13^C NMR of 6](#__RefHeading___Toc313788083) 69

[Copy of ^1^H NMR of 31](#__RefHeading___Toc313788086) 70

[Copy of ^13^C NMR of 31](#__RefHeading___Toc313788085) 71

[Copy of ^1^H NMR of 32](#__RefHeading___Toc313788088) 72

[Copy of ^13^C NMR of 32](#__RefHeading___Toc313788087) 73

[Copy of ^1^H NMR of 33](#__RefHeading___Toc313788090) 74

[Copy of ^13^C NMR of 33](#__RefHeading___Toc313788089) 75

[Copy of ^1^H NMR of 7](#__RefHeading___Toc313788092) 76

[Copy of ^13^C NMR of 7](#__RefHeading___Toc313788091) 77

[Copy of ^1^H NMR of 36](#__RefHeading___Toc313788094) 78

[Copy of ^13^C NMR of 36](#__RefHeading___Toc313788093) 79

[Copy of ^1^H NMR of 37](#__RefHeading___Toc313788094) 80

[Copy of ^13^C NMR of 37](#__RefHeading___Toc313788095) 81

[Copy of ^1^H NMR of 40](#__RefHeading___Toc313788094) 82

[Copy of ^13^C NMR of 40](#__RefHeading___Toc313788095) 83

[Copy of ^1^H NMR of 42](#__RefHeading___Toc313788094) 84

[Copy of ^13^C NMR of 42](#__RefHeading___Toc313788093) 85

[Copy of ^1^H NMR of 8](#__RefHeading___Toc313788094) 86

[Copy of ^13^C NMR of 8](#__RefHeading___Toc313788095) 87

[Copy of ^1^H NMR of 46](#__RefHeading___Toc313788094) 88

[Copy of ^13^C NMR of 46](#__RefHeading___Toc313788095) 89

[Copy of ^1^H NMR of 47](#__RefHeading___Toc313788094) 90

[Copy of ^13^C NMR of 47](#__RefHeading___Toc313788093) 91

[Copy of ^1^H NMR of 48](#__RefHeading___Toc313788094) 92

[Copy of ^13^C NMR of 48](#__RefHeading___Toc313788095) 93

[Copy of ^1^H NMR of 49](#__RefHeading___Toc313788094) 94

[Copy of ^13^C NMR of 49](#__RefHeading___Toc313788095) 95

[Copy of ^1^H NMR of 50](#__RefHeading___Toc313788094) 96

[Copy of ^13^C NMR of 50](#__RefHeading___Toc313788093) 97

[Copy of ^1^H NMR of 9](#__RefHeading___Toc313788094) 98

[Copy of ^13^C NMR of 9](#__RefHeading___Toc313788094) 99

Copy of LC-MS chromatogram for 6 100

**S-1. Detailed reaction procedures of all the compounds**

**Material and General Methods.** All commercially available solvents and reagents were used without further purification. Melting points of compounds were measured on a RY-1 melting point apparatus. Column chromatography was carried out on silica gel (200-300 mesh, Qingdao Ocean Chemical Company, China). ^1^H and ^13^C NMR spectra were measured on a Bruker AV-400 spectrometer. Chemical shifts are reported as δ values from an internal tetramethylsilane standard. Mass spectral data were obtained on Agilent 1100 LC/DAD/MSD or Q-Tof Micro MS/MS spectrometer.

**Abbreviations**: **NMR** (nuclear magnetic resonance), **MS** (mass spectrometer), **(Et)_2_O** (ether), **EtOH** (ethanol),  **THF** (tetrahydrofuran), **EtOAc (**ethyl acetate**)**, **Na_2_SO_4_** (sodium sulfate), **NaOH** (sodium hydroxide), **HCl** (hydrogen chloride), **CH_2_Cl_2_** (dichloromethane), **HATU** (1-[Bis(dimethylamino)methylene]-1H-1,2,3-triazolo[4,5-b]pyridinium 3-oxid hexafluorophosphate), **DIPEA** (*N,N*-diisopropylethylamine), **NH_4_Cl** (ammonium chloride), **MeOH** (methanol), **LiOH·H_2_O** (lithium hydroxide monohydrate), **DMAP** (4-dimethylaminopyridine), **DMF** (N, N-dimethylformamide), **DCC** (dicyclohexylcarbodiimide), **TBDMSCl** (tert-butyldimethylsilyl chloride), **EDCI** (carbodiimide), **HOBt** (1-hydroxybenzotriazole), **TFA** (trifluoroacetic acid), **NaHCO_3_** (sodium bicarbonate), **Et_3_N** (triethylamine), **Pd-C** (palladium on carbon), **T_3_P** (1-propanephosphonic acid cyclic anhydride), **CH_3_CN** (acetonitrile), **AcOH** (acetic acid), **DEPC** (diethyl cyanophosphonate).

**5-Chloro-1H-pyrrolo[2,3-c]pyridine-2-carboxylic acid ethyl ester (12).** A reported procedure was used to prepare compound **12**.^15^ To a mixture of potassium ethoxide (0.25 g，3.0 mmol) in (Et)_2_O/EtOH (10:1, 22 mL), diethyl oxalate (0.4 mL，2.9 mmol) was added. After the mixture was stirred for 30 min under a nitrogen atmosphere at room temperature, a solution of 2-chloro-4-methyl-5-nitropyridine (**10**, 0.5 g，2.9 mmol) in diethyl ether (5 mL) was added. The mixture was stirred at room temperature over night and then concentrated vacuo to give crude product **11** as an orange oil, which was used for the next reaction without further purification. The crude product **11** was redissolved in EtOH/THF (2:1, 54 mL), and then iron powder (1.3 g, 23.2 mmol) and saturated ammonium chloride solution (18 mL) were added. The mixture heated at reflux for 2h. After cooling to room temperature, the reaction mixture was filtered through Celite, and the insoluble substance was washed with EtOAc (50 mL × 3). The filtrate was then concentrated in vacuo, and the residue was redissolved in EtOAc (50 mL) and washed with brine (50 mL×3), dried over Na_2_SO_4_, filtered, and concentrated in vacuo. The crude product was purified by column chromatography over silica gel [petroleum ether-EtOAc (10:1)] to give **12** as a white solid (0.24 g, 32%). M.p. 200-202 °C. ^1^H NMR (400 MHz, CDCl_3_): 1.44 (t, *J* = 7.2, 3H, CH_3_), 4.47 (q, *J* = 7.2, 2H, CH_2_), 7.15 (s, 1H, Pyrrole-H), 7.62 (s, 1H, Py-H), 8.75 (s, 1H, Py-H), 9.96 (br *s*, 1H, NH). ^13^C NMR (100 MHz, CDCl_3_): 161.4 (C=O), 141.0 (ArC), 135.1 (ArC), 135.0 (ArC), 132.7 (ArC), 132.6 (ArC), 115.9 (ArC), 106.4 (ArC), 62.0 (CH_2_), 14.3 (CH_3_). ESI-MS *m/z*: 224.0 [*M*-H]^-^.

**5-Chloro-1*H*-pyrrolo[2,3-c]pyridine-2-carboxylic acid (13).** To a solution of **12** (0.55 g，2.5 mmol) in ethanol (25 mL) under ice bath, a solution of NaOH (2M, 1.5 mL) was added dropwise. The mixture heated at reflux for 2h and then concentrated in vacuo. The residue redissolved in H_2_O (50 mL) and was adjusted to pH = 2~3 with HCl aqueous (1 M) and the solid was participated. The precipitate was filtered, washed with water, and dried to give **13**, which was almost a pure product, and was used for the next reaction without further purification. High degree of purity for **13** as a white solid was obtained by column chromatography over a C18 reversephase column to give **13** as a white solid (0.4 g, 83%). M.p. >300℃. ^1^H NMR (400 MHz, *d_6_*-DMSO): 6.89 (s, 1H, Pyrrole-H), 7.61 (s, 1H, Py-H), 8.62 (s, 1H, Py-H), 12.20 (br *s*, 1H, NH). ^13^C NMR (100 MHz, *d_6_*-DMSO): 163.8 (C=O), 140.3 (ArC), 138.7 (ArC), 135.0 (ArC), 134.8 (ArC), 132.5 (ArC), 114.5 (ArC), 102.6 (ArC). ESI-MS *m/z*: 197.6[*M*-H]^-^.

***Tert*-Butyl [1-(*S*)-(4-Fluorobenzyl)-2-(4-hydroxypiperidin-1-yl)-2-oxoethyl] carbamate (15).** (*S*)-N-BOC-4-fluorophenylalanine (**14**, 15.6 g, 55.1 mmol) was dissolved in anhydrous CH_2_Cl_2_ (160 mL), to which HATU (25.1 g, 66.1 mmol), DIPEA (11.5 mL, 66.1 mmol) were added at 0 °C. After the mixture was stirred for 10 min at room temperature, 4-hydroxypiperidine hydrochloride (6.75 g, 66.1 mmol) was added. The mixture was stirred at room temperature over night and then concentrated vacuo. The residue was redissolved in EtOAc (160 mL) and washed with brine (160 mL×3), dried over Na_2_SO_4_, filtered, and concentrated in vacuo. The crude product was purified by column chromatography over silica gel [petroleum ether-EtOAc (1:1)] to give **15** as a white solid (19.7 g, 98%). M.p. 127-129 °C. ^1^H NMR (400 MHz, CDCl_3_): 1.41 (s, 9H, CH_3_), 1.04-1.51 (m, 1H, CH_2_), 1.75-1.85 (m, 3H, CH_2_), 2.84-2.96 (m, 3H, PhCH_2_+CH_2_+CH_2_), 3.15-3.29 (m, 1H, CH_2_), 3.46-3.61 (m, 1H, CH_2_), 3.82-4.00 (m, 2H, PhCH_2_+CH_2_), 4.81-4.83 (m, 1H, OH), 5.44 (t, *J* = 11.2 Hz, 1H, CH), 6.94-6.99 (m, 2H, ArH), 7.14-7.15 (m, 2H, ArH). ^13^C NMR (100 MHz, CDCl_3_): 169.75 (C=O), 169.70 (C=O of isomer), 161.93 (d, *J* = 243.8 Hz, ArC), 161.89 (d, *J* = 243.7 Hz, ArC of isomer), 155.1 (C=O), 132.26 (d, *J* = 13.0 Hz, ArC), 132.23 (d, *J* = 13.0 Hz, ArC of isomer), 131.1 (ArC), 131.0 (ArC), 115.4 (d, *J* = 8.0 Hz, ArC), 115.2 (d, *J* = 8.1 Hz, ArC), 79.78 (C), 79.75 (C of isomer), 66.4 (CH), 66.6 (CH of isomer), 50.92 (CH), 50.88 (CH of isomer), 42.6 (CH_2_), 39.2 (CH_2_), 39.3 (CH_2_ of isomer), 39.12 (CH_2_), 39.19 (CH_2_ of isomer), 34.03 (CH_2_), 34.19 (CH_2_ of isomer), 33.62 (CH_2_ ), 33.52 (CH_2_ of isomer), 28.3 (CH_3_). MS (ESI) *m/z*: 367.2 [*M*+H]^+^.

**(*S*)-2-Amino-3-(4-fluorophenyl)-1-(4-hydroxypiperidin-1-yl) propan-1-one (16).** To a solution of **15** (19.0 g, 51.9 mmol) in anhydrous CH_2_Cl_2_ (50 mL) was added dropwise a solution of HCl in CH_2_Cl_2_ (2N, 50 mL) at 0 °C. The reaction mixture stirred at room temperature over night and then concentrated in vacuo to give a white solid, which was almost a pure product, and was used for the next reaction without further purification. Crystallization of the crude product from EtOH gave **16** as a white solid (14.0 g, 89%). M.p. 139-141 °C. ^1^H NMR (400 MHz, CD_3_OD): 0.93-1.39 (m, 2H, CH_2_), 1.43-1.80 (m, 2H, CH_2_), 2.76-2.82 (m, 1H, PhCH_2_), 3.06-3.08 (m, 2H, CH+CH_2_), 3.12-3.16 (m, 1H, CH_2_), 3.33-3.54 (m, 1H, CH_2_), 3.73-3.81 (m, 1H, CH_2_), 3.83-4.03 (m, 1H, PhCH_2_), 4.63 (t, *J* = 6.4 Hz 1H, CH), 7.07-7.12 (m, 2H, ArH), 7.25-7.30 (m, 2H, ArH), 8.45 (br *s*, 1H, NH). ^13^C NMR (100 MHz, CD_3_OD): 168.30 (C=O), 168.38 (C=O of isomer), 163.90 (d, *J* = 243.8 Hz, ArC), 163.88 (d, *J* = 243.7 Hz, ArC of isomer), 132.67 (ArC), 132.65 (ArC of isomer), 132.58 (ArC), 132.57 (ArC of isomer), 131.62 (d, *J* = 3.4 Hz, ArC), 131.59 (d, *J* = 3.4 Hz, ArC of isomer), 116.9 (d, *J* = 9.0 Hz, ArC), 116.7 (d, *J* = 9.0 Hz,ArC), 67.1 (CH), 66.8 (CH of isomer) 52.03 (CH), 51.99 (CH of isomer), 44.0 (CH_2_), 43.9 (CH_2_ of isomer) 40.8 (CH_2_), 40.6 (CH_2_ of isomer), 38.10 (CH_2_), 38.13 (CH_2_ of isomer), 35.0 (CH_2_), 34.7 (CH_2_ of isomer), 34.4 (CH_2_), 34.1 (CH_2_ of isomer). MS (ESI) *m/z*: 267.2 [*M*+H]^+^.

**5-Chloro-1*H*-pyrrolo[2,3-c]pyridine-2-carboxylic acid [1-(*S*)-(4- fluorobenzyl)-2-(4-hydroxypiperidin-1-yl)-2-oxoethyl]amide (PSN-357).** Following the procedure for preparation of **15**, the amidation reaction of 5-Chloro-1H-pyrrolo[2,3-c]pyridine-2-carboxylic acid (**13**) with **16** was carried out to give **PSN-357** as a white solid (69%). M.p. 137-139 °C. ^1^H NMR (400 MHz, *d_6_*-DMSO): 1.15-1.24 (m, 2H, CH_2_), 1.57-1.67 (m, 2H, CH_2_), 2.91-3.29 (m, 4H, PhCH_2_+CH_2_+CH), 3.65-4.04 (m, 3H, PhCH_2_+CH_2_), 4.75 (s, 1H, OH), 5.15-5.17 (m, 1H, CH), 7.06 (d, *J* = 3.6, 2H, Pyrrole-H+NH), 7.34 (d, *J* = 9.6 Hz, 3H, ArH), 7.77 (s, 1H, ArH), 8.56 (s, 1H, Py-H), 9.22 (t, *J* = 8.8 Hz, 1H, Py-H), 12.27 (s, 1H, NH). ^13^C NMR (100 MHz, *d_6_*-DMSO): 169.07 (C=O), 169.01 (C=O of isomer), 161.48 (d, *J* = 237.2 Hz, ArC), 161.45 (d, *J* = 240.4 Hz, ArC of isomer), 160.0 (C=O), 159.9 (C=O of isomer), 139.7 (ArC), 136.7 (ArC), 135.5 (ArC), 134.9 (ArC), 134.4 (d, *J* = 2.8 Hz, ArC), 134.3 (d, *J* = 2.8 Hz, ArC of isomer ), 133.0 (ArC), 131.7 (ArC), 131.6 (ArC), 115.6 (ArC), 115.3 (d, *J* = 4.7 Hz, ArC), 115.1 (d, *J* = 4.6 Hz, ArC), 102.2 (ArC), 66.2 (CH), 65.4 (CH of isomer), 50.8 (CH), 43.2 (CH_2_), 42.8 (CH_2_ of isomer), 36.8 (PhCH_2_), 36.9 (PhCH_2_ of isomer), 35.1 (CH_2_), 34.7 (CH_2_ of isomer), 34.5 (CH_2_), 34.0 (CH_2_ of isomer). MS (ESI) *m/z*: 444.9 [*M*+H]^+^. HRMS (ESI) [M+H]^+^ m/z: 445.1437 (calcd for C_22_H_23_ClFN_4_O_3_, 445.1443).

**N-Methoxycarbonylmethyl-3*α*, 7*α*, 12*α*-trihydroxy-5*β*-cholan-24-amide (18).** Cholic acid (**17**, 204.0 mg, 0.5 mmol) was dissolved anhydrous THF (4 mL), to which HATU (228 mg, 0.6 mmol), DIPEA (261 μL, 1.5 mmol) were added at 0 °C. After the mixture was stirred for 10 min at room temperature, glycine methyl ester hydrochloride (125 mg, 1.0 mmol) was added. The mixture was stirred at room temperature for 2h at 30°C and quenched by adding saturated NH_4_Cl (10 mL), extracted with EtOAc (10 mL×3). The combined organic layers were washed with saturated NaHCO_3_ (20 mL) and brine (20 mL), dried over Na_2_SO_4_, filtered, and concentrated in vacuo. The crude product was purified by column chromatography over silica gel [CH_2_Cl_2_-MeOH (10:1)] to give **18** as a white solid (150 mg, 63%). M.p. 131-133 °C. ^1^H NMR (400 MHz, CDCl_3_): 0.66 (s, 3H, CH_3_), 0.87 (s, 3H, CH_3_), 0.98 (d, *J* = 5.6 Hz, 3H, CH_3_), 3.38-3.50 (m, 1H, CH), 3.73 (s, 3H, CH_3_), 3.79-3.86 (m, 1H, CH), 3.93-3.97 (m, 1H, CH), 4.00 (d, *J* = 5.2 Hz, 2H, CH_2_), 6.70 (t, *J* = 5.2 Hz, 1H, NH). ^13^C NMR (100 MHz, CDCl_3_): 174.7 (C=O), 170.9 (C=O), 73.1, 71.7 and 68.5 (CH-OH of the cholic acid residues), 60.4 (CH_3_O), 52.4, 46.3, 46.2, 41.6, 41.3, 41.2, 39.3, 39.2, 35.2, 34.7, 34.6, 32.5, 31.4, 30.0, 28.0, 27.6, 26.4 and 23.2 (CH_2_, CH and C of the cholic acid residues), 22.4, 17.3 and 12.4 (CH_3_ of the cholic acid residues). MS (ESI) *m/z*: 462.3 [*M*-OH]^-^.

**N-Carboxymethyl-3*α*, 7*α*, 12*α*-trihydroxy-5*β*-cholan-24-amide (19).** To a solution of **18** (2.20 g, 4.60 mmol) in THF/H_2_O (2:1, 30 mL), LiOH·H_2_O (0.58 g, 13.80 mmol) was added. The reaction mixture stirred at room temperature over night. After most organic solvent was removed by evaporation, the residue was adjusted to pH = 2~3 with HCl aqueous (1 M) and the solid was participated. The precipitate was filtered, washed with water, and dried to give **19**, which was almost a pure product, and was used for the next reaction without further purification. Crystallization of the crude product from ethanol gave **19** as a white solid (1.60 g, 76%). M.p.145-147 °C. ^1^H-NMR (400 MHz, CD_3_OD): 0.73 (s, 3H, CH_3_), 0.94 (s, 3H, CH_3_), 1.05 (d, *J* = 7.6 Hz, 3H, CH_3_), 3.35-3.43 (m, 1H, CH), 3.79-3.85 (m, 1H, CH), 3.91 (s, 2H, CH_2_), 3.95-4.00 (m, 1H, CH). ^13^C-NMR (100 MHz, CD_3_OD): 175.9 (C=O), 171.7 (C=O), 72.7, 71.5 and 67.7 (CH-OH of the cholic acid residues), 46.7, 46.1, 41.8, 41.6, 40.4, 39.6, 39.1, 35.4, 35.1, 34.5, 34.4, 32.4, 31.7, 29.8, 28.2, 27.3, 26.5 and 22.9 (CH_2_, CH and C of the cholic acid residues), 21.8, 16.3 and 11.6 (CH_3_ of the cholic acid residues). MS (ESI) *m/z*: 448.3 [*M*-OH]^-^.

**N-(2-{[(S)*-*2-(5-Chloro-1*H*-pyrrolo[2,3-c]pyridine-2-carboxamido)-2-(4-fluorobenzyl)-1-oxoethyl]-piperidin-4-yloxy}-2-oxoethyl)-3*α*, 7*α*, 12*α*-trihydroxy-5*β*-cholan-24-amide (1).** To a mixture of **19** (0.32 g, 0.69 mmol) and catalytic amount of DMAP (18 mg, 0.15 mmol) in DMF (6 mL) under ice bath, DCC (0.25 g, 1.21mmol) was added. After the mixture was stirred for 1h at room temperature, a solution of **PSN-357** (0.15 g, 0.34 mmol) in DMF (4 mL) was added dropwise. The mixture was stirred at room temperature over night and then concentrated in vacuo. The residue redissolved in EtOAc (100 mL) and cooled at 0 °C over night and then filtrated, the filtrate was concentrated in vacuo. High degree of purity for **1** as a white solid was obtained using preparative-reverse phase HPLC (33 mg, 10.9%). M.p.169-171 °C. ^1^H NMR (400 MHz, CD_3_OD): 0.68 (d, *J* = 34.8 Hz, 3H, CH_3_), 0.89 (d, *J* = 23.6 Hz, 3H, CH_3_), 1.02 (dd, *J* = 20.0, 6.4 Hz, 3H, CH_3_), 3.11 (ddd, *J* = 13.2, 7.2, 2.4 Hz, 1H, CH), 3.20 (dd, *J* = 13.6, 7.6 Hz, 1H, CH), 3.38-3.47 (m, 2H, CH+PhCH_2_), 3.57-3.72 (m, 4H, CH_2_), 3.80-3.97 (m, 1H, PhCH_2_), 3.91 (d, *J* = 9.2 Hz, 2H, CH_2_), 4.81-5.06 (m, 1H, CH), 5.34 (q, *J* = 7.2 Hz, 1H, CH), 7.04 (td, *J* = 8.8, 2.0 Hz, 2H, ArH), 7.21 (d, *J* = 4.8 Hz, 1H, ArH), 7.31-7.36 (m, 2H, ArH), 7.76 (s, 1H, ArH), 8.63 (d, *J* = 4.4 Hz, 1H, ArH). ^13^C NMR (100 MHz, CD_3_OD): 175.96 (C=O), 175.99 (C=O of isomer), 169.91 (C=O), 169.88 (C=O of isomer), 169.1 (C=O), 169.2 (C=O of isomer), 162.0 (d, *J* = 323.4 Hz, ArC), 160.2 (C=O ), 160.3 (C=O of isomer), 138.5 (ArC), 137.3 (ArC), 135.8 (ArC), 133.9 (ArC), 132.7 (ArC), 132.4 (ArC), 131.0 (ArC), 130.9 (ArC), 115.7 (ArC), 115.0 (ArC), 114.7 (ArC), 101.8 (ArC), 72.6, 71.5, 69.6, 69.5 (from isomer) and 67.6 (CH-OH of the cholic acid residues and CH-O of piperidine), 50.7 (CH), 50.5 (CH of isomer ), 46.57, 4.63 (from isomer) and 46.02, 46.08 (from isomer) (C and CH of the cholic acid residues), 42.4 (CH_2_), 41.8, 41.5, 41.6 (from isomer), 41.0, 40.9 (from isomer), 39.6 and 39.0, 38.9 (from isomer) (CH_2_ and CH of the cholic acid residues), 36.9 (PhCH_2_), 37.0 (PhCH_2_ of isomer), 35.43, 35.39 (from isomer), 35.1, 34.45, 34.49 (from isomer), 32.2, 32.3 (from isomer) and 31.7 (CH_2_ and C of the cholic acid residues), 30.3 (CH_2_), 29.8, 28.1, 28.2 (from isomer), 27.3, 26.4 and 22.85, 22.78 (from isomer) (CH_2_ and CH of the cholic acid residues), 21.8, 16.26, 16.33 (from isomer) and 11.6 (CH_3_ of the cholic acid residues). MS (ESI) *m/z*: 892.3 [*M*+H]^+^. HR-MS (ESI, M+Na) m/z: calcd for C_48_H_63_ClFN_5_NaO_8_ 914.4247, found 914.4241.

**N-Methoxycarbonylmethyl-N’-methyl-3*α*, 7*α*, 12*α*-trihydroxy-5*β*-cholan-24-amide (20).** Following the procedure for preparation of **18**, the amidation reaction of cholic acid **17** with sarcosine methylester was carried out to give **20** as a white solid (48.6%). M.p.110-112 °C. ^1^H NMR (400 MHz, CD_3_OD): 0.74 (s, 3H, CH_3_), 0.94 (s, 3H, CH_3_), 1.07 (d, *J* = 6.4 Hz, 3H, CH_3_), 3.15 (s, 3H, CH_3_), 3.35-3.43 (m, 1H, CH), 3.74 (s, 3H, CH_3_), 3.79-3.85 (m, 1H, CH), 3.96-3.98 (m, 1H, CH), 4.13 (s, 2H, CH_2_). ^13^C NMR (100 MHz, CD_3_OD): 174.2 (C=O), 168.6 (C=O), 71.2, 70.0 and 66.2 (CH-OH of the cholic acid residues), 49.7 (CH_3_O), 47.6, 45.1, 44.6, 40.3, 40.1, 38.1, 37.6, 34.5, 34.0, 33.6, 33.0, 32.9, 29.4, 28.3, 28.1, 26.7, 25.8, 25.0 and 21.4 (CH_2_, CH and C of the cholic acid residues), 20.3, 14.9 and 10.1 (CH_3_ of the cholic acid residues). MS (ESI) *m/z*: 494.3 [*M*+H]^+^.

**N-Carboxymethyl-N’-methyl-3*α*, 7*α*, 12*α*-trihydroxy-5*β*-cholan-24-amide (21).** Following the procedure for preparation of **20**, the hydrolysis reaction of **20** with LiOH was carried out to give **21** as a white solid (79%). M.p. 133-135 °C. ^1^H NMR (400 MHz, CD_3_OD): 0.74 (s, 3H, CH_3_), 0.94 (s, 3H, CH_3_), 1.07 (d, *J* = 6.4 Hz, 3H, CH_3_), 3.14 (s, 3H, CH_3_), 3.37-3.43 (m, 1H, CH), 3.80-3.84 (m, 1H, CH), 3.96-3.98 (m, 1H, CH), 4.10 (s, 2H, CH_2_). ^13^C NMR (100 MHz, CD_3_OD): 175.6 (C=O), 171.5 (C=O), 72.7, 71.5 and 67.7 (CH-OH of the cholic acid residues), 49.0, 46.6, 46.1, 41.8, 41.6, 39.6, 39.1, 35.9 (CH_3_-N), 35.5, 35.1, 34.5, 34.4, 30.9, 29.8, 29.6, 28.2, 27.3, 26.5 and 22.9 (CH_2_, CH and C of the cholic acid residues), 21.8, 16.5 and 11.6 (CH_3_ of the cholic acid residues). MS (ESI) *m/z*: 462.3 [*M*-OH]^-^.

**N-(2-{[(S)*-*2-(5-Chloro-1*H*-pyrrolo[2,3-c]pyridine-2-carboxamido)-2-(4-fluorobenzyl)-1-oxoethyl]-piperidin-4-yloxy}-2-oxoethyl)-N’-methyl-3*α*, 7*α*, 12*α*-trihydroxy-5*β*-cholan-24-amide (2).** Following the procedure for preparation of **1**, the esterification reaction between **21** and **PSN-357** was carried out to give **2** as a white solid (21%). m.p. 183-185 °C. ^1^H NMR (400 MHz, CD_3_OD): 0.65 (s, 3H, CH_3_), 0.87 (d, *J* = 28.4 Hz, 3H, CH_3_), 0.98-1.06 (m, 3H, CH_3_), 3.05-3.21 (m, 5H, CH+CH_3_), 3.34-3.79 (m, 6H, CH+PhCH_2_+CH_2_), 3.88-3.96 (m, 1H, PhCH_2_), 4.06-4.24 (m, 2H, CH_2_), 4.94-4.98 (m, 1H, CH), 5.32 (q, *J* = 8.0 Hz, 1H, CH), 7.02 (td, *J* = 8.4, 4.0 Hz, 2H, ArH), 7.22 (d, *J* = 3.6 Hz, 1H, ArH), 7.29-7.34 (m, 2H, ArH), 7.79 (s, 1H, ArH), 8.64 (d, *J* = 4.0 Hz, 1H, ArH). ^13^C NMR (100 MHz, CD_3_OD): 175.68 (C=O), 175.63 (C=O of isomer), 169.95 (C=O), 169.90 (C=O of isomer), 168.74 (C=O), 168.70 (C=O of isomer), 162.0 (d, *J* = 242.6 Hz, ArC), 160.3 (C=O), 160.2 (C=O of isomer), 138.33 (ArC), 138.26 (ArC of isomer), 137.4 (ArC), 135.8 (ArC), 133.9 (ArC), 133.8 (ArC of isomer), 132.68 (ArC), 132.65 (ArC of isomer), 132.4 (ArC), 131.1 (ArC), 131.0 (ArC), 115.7 (ArC), 115.0 (d, *J* = 1.8 Hz, ArC), 114.8 (d, *J* = 1.9 Hz, ArC), 101.8 (ArC), 72.6, 71.5, 69.63, 69.53 (from isomer) and 67.7, 67.6 (from isomer) (CH-OH of the cholic acid residues and CH-O of piperidine), 50.7 (CH), 50.5 (CH of isomer), 49.8, 49.7 (from isomer), 46.53, 46.50 (from isomer) and 46.0, 46.1 (from isomer) (C and CH of the cholic acid residues), 42.5 (CH_2_), 42.4 (CH_2_ of isomer), 41.8, 41.6, 41.5 (from isomer), 39.62, 39.56 (from isomer) and 39.1 (CH_2_ and CH of the cholic acid residues), 39.0 (CH_2_), 38.9 (CH_2_ of isomer), 36.9 (PhCH_2_), 37.0 (PhCH_2_ of isomer), 36.1 (CH_3_), 35.44, 35.38 (from isomer), 35.0, 35.1 (from isomer), 34.4, 34.5 (from isomer), 32.4 and 30.89, 30.85 (from isomer) (CH_2_ and C of the cholic acid residues), 30.32 (CH_2_), 30.28 (CH_2_ of isomer) 29.8 (CH_2_), 29.7 (CH_2_ of isomer), 29.49 (CH_2_), 29.45 (CH_2_ of isomer), 28.2, 28.1 (from isomer), 27.33, 27.28 (from isomer) 26.5, 26.4 (from isomer) and 22.85, 22.78 (from isomer) (CH_2_ and CH of the cholic acid residues), 21.79, 21.75 (from isomer), 16.44, 16.36 (from isomer) and 11.63, 11.57 (from isomer) (CH_3_ of the cholic acid residues). MS (ESI) *m/z*: 906.3 [*M*+H]^+^.

**(*S*)-1-[2S-(5-Chloro-1H-pyrrolo[2,3-c]pyridine-2-carboxamido)-2-(4-fluorobenzyl)-1-oxoethyl]-piperidine-4-yl 2-aminopropionate (26).** To a mixture of Boc-L-alanine (127 mg, 0.67 mmol) and catalytic amount of DMAP (16 mg, 0.13 mmol) in CH_2_Cl_2_ (10 mL) under ice bath, DCC (167 mg, 0.81 mmol) was added. After the mixture was stirred for 45 min at room temperature, **PSN-357** (250 mg, 0.56 mmol) was added. The mixture was stirred at room temperature over night and then filtered. The filtrate was concentrated and redissolved in EtOAc (100 mL) and cooled at 0 °C over night and then filtrated, the filtrate was concentrated in vacuo to give **22** as a white solid, which was used for the next reaction without further purification. To a solution of the crude product **22** (0.14 g, 0.23 mmol) in EtOAc (10 mL) was added dropwise a solution of HCl in EtOAc (4N, 3 mL) at 0 °C. The reaction mixture stirred at room temperature for 2h and then concentrated in vacuo to give a white solid, which was almost a pure product, and was used for the next reaction without further purification. Crystallization of the crude product from ethanol gave **26** as a white solid (101 mg, 40% for 2 steps). m.p. 217-219 °C. ^1^H NMR (400 MHz, CD_3_OD): 1.09-1.70 (m, 5H, CH_3_+CH_2_), 1.81-1.93 (m, 2H, CH_2_), 3.12-3.19 (m, 2H, PhCH_2_+CH_2_), 3.47-3.55 (m, 2H, CH_2_), 3.77-3.82 (m, 2H, PhCH_2_+CH_2_), 4.09 (t, *J* = 7.2 Hz, 1H, CH), 5.03-5.05 (m, 1H, CH), 5.32 (t, *J* = 7.6 Hz, 1H, CH), 6.99-7.05 (m, 2H, ArH+Pyrrole-H), 7.30-7.34 (m, 2H, ArH), 7.42 (s, 1H, ArH), 8.14 (s, 1H, Py-H), 8.91 (s, 1H, Py-H). ^13^C NMR (100 MHz, CD_3_OD): 169.9 (C=O), 168.95 (C=O), 168.97 (C=O of isomer), 162.0 (d, *J* = 243.3 Hz, ArC), 159.2 (C=O), 142.22 (ArC), 142.18 (ArC of isomer), 138.4 (ArC), 132.64 (ArC), 132.61 (ArC of isomer), 132.33 (ArC), 132.27 (ArC of isomer), 131.1 (d, *J* = 4.3 Hz, ArC), 131.2 (d, *J* = 3.7 Hz, ArC of isomer), 131.0 (d, *J* =1.8 Hz, ArC), 130.9 (d, *J* = 4.1 Hz, ArC of isomer), 117.9 (ArC), 115.1 (d, *J* = 7.4 Hz, ArC), 115.0 (d, *J* = 7.2 Hz, ArC), 114.9 (ArC), 114.8 (ArC), 103.1 (ArC), 71.5 (CH), 71.4 (CH of isomer), 50.99 (CH), 50.92 (CH of isomer), 48.6 (CH), 42.5 (CH_2_), 39.03 (CH_2_), 38.96 (CH_2_ of isomer), 36.8 (PhCH_2_), 36.9 (PhCH_2_ of isomer), 30.3 (CH_2_), 30.1 (CH_2_ of isomer), 29.6 (CH_2_), 29.7 (CH_2_ of isomer), 19.2 (CH_3_). MS (ESI) *m/z*: 516.0 [*M*+H]^+^.

**(*S*)-N-(1-Methyl-2-{[(S)*-*2-(5-chloro-1*H*-pyrrolo[2,3-c]pyridine-2-carboxamido)-2-(4-fluorobenzyl)-1-oxoethyl]-piperidin-4-yloxy}-2-oxoethyl)-3*α*, 7*α*, 12*α*-trihydroxy-5*β*-cholan-24-amide (3).** Cholic acid (**17**, 88.70 mg, 0.22 mmol) was dissolved in 4 mL of anhydrous DMF, to which HATU (93.6 mg, 0.25 mmol), DIPEA (126 μL, 0.72 mmol) were added at 0 °C. After the mixture was stirred for 10 min at room temperature, compound **26** (0.10 g, 0.18 mmol) was added. The mixture was stirred at room temperature over night and then concentrated in vacuo. High degree of purity for **3** as a white solid was obtained using preparative-reverse phase HPLC (50 mg, 30.7%). m.p.180-182 °C. ^1^H NMR (400 MHz, CD_3_OD): 0.65 (d, *J* = 42.0 Hz, 3H, CH_3_), 0.87 (d, *J* = 29.2 Hz, 3H, CH_3_), 1.01 (dd, *J* = 24.4, 6.4 Hz, 3H, CH_3_), 1.38 (t, *J* = 7.6 Hz, 3H, CH_3_), 3.09-3.14 (m, 1H, CH), 3.20 (dd, *J* = 13.2, 8.0 Hz, 1H, CH), 3.35-3.49 (m, 2H, CH+PhCH_2_), 3.54-3.63 (m, 2H, CH_2_), 3.67-3.79 (m, 2H, CH_2_), 3.87-3.95 (m, 1H, PhCH_2_), 4.32-4.36 (m, 1H, CH), 5.33 (q, *J* = 7.6 Hz, 1H, CH), 7.03 (td, *J* = 8.8, 3.2 Hz, 2H, ArH+Pyrrole-H), 7.17 (d, *J* = 6.0 Hz, 1H, ArH), 7.31-7.36 (m, 2H, ArH), 7.69 (s, 1H, Py-H), 8.09 (s, 1H, NH), 8.10 (s, 1H, NH), 8.58 (d, *J* = 4.4 Hz, 1H, Py-H). ^13^C NMR (100 MHz, CD_3_OD): 175.2 (C=O), 175.3 (C=O of isomer), 172.17 (C=O), 172.23 (C=O of isomer), 169.93 (C=O), 169.98 (C=O of isomer), 162.0 (d, *J* = 242.7 Hz, ArC), 160.3 (C=O), 160.4 (C=O of isomer), 139.03 (ArC), 138.98 (ArC of isomer), 136.9 (ArC), 136.8 (ArC of isomer), 135.5 (ArC), 134.3 (ArC), 134.2 (ArC of isomer), 132.7 (d, *J* = 3.1 Hz, ArC), 132.6 (d, *J* = 3.0 Hz, ArC of isomer), 132.5 (ArC), 131.1 (ArC), 131.0 (ArC), 115.54 (ArC), 115.50 (ArC of isomer), 115.0 (d, *J* = 2.8 Hz, ArC), 114.8 (d, *J* = 2.8 Hz, ArC), 101.8 (ArC), 72.61, 72.64 (from isomer), 71.5, 69.5, 69.3 (from isomer) and 67.68, 67.63 (from isomer) (CH-OH of the cholic acid residues and CH-O of piperidine), 50.7 (CH), 50.5 (CH of isomer), 48.41 (CH), 48.35 (CH of isomer), 46.7 and 46.1, 46.0 (from isomer) (C and CH of the cholic acid residues), 42.42 (CH_2_), 42.37 (CH_2_ of isomer), 41.72, 41.74 (from isomer), 41.6, 41.5 (from isomer), 39.6, 39.5 (from isomer) and 39.0 (CH_2_ and CH of the cholic acid residues), 38.96 (CH_2_), 38.84 (CH_2_ of isomer), 37.0 (PhCH_2_), 36.9 (PhCH_2_ of isomer), 35.5, 35.1, 35.0 (from isomer), 34.42, 34.48 (from isomer), 34.37 (from isomer), 32.2, 32.3 (from isomer) and 31.8, 31.7 (from isomer) (CH_2_ and C of the cholic acid residues), 30.25 (CH_2_), 30.17 (CH_2_ of isomer), 29.7 (CH_2_), 29.6, 28.1, 28.2 (from isomer), 27.3, 26.4, 26.5 (from isomer) and 22.85, 22.78 (from isomer) (CH_2_ and CH of the cholic acid residues), 21.76, 21.79 (from isomer) and 16.3,16.4 (from isomer) (CH_3_ of the cholic acid residues), 15.80 (CH_3_), 15.85 (CH_3_ of isomer), 11.6, 11.7 (from isomer) (CH_3_ of the cholic acid residues). MS (ESI) *m/z*: 906.2 [*M*+H]^+^. HRMS (ESI) [M+H]^+^ m/z: 906.4578 (calcd for C_49_H_66_ClFN_5_O_8_, 906.4584).

**(*S*)-1-[(*S*)-2-(5-Chloro-1H-pyrrolo[2,3-c]pyridine-2-carboxamido)-2-(4-fluorobenzyl)-1-oxoethyl]-piperidine-4-yl 2-amino-3-methylbutanoate (27).** Following the procedure for preparation of **26**, the esterification reaction between N-(tert-butoxycarbonyl)-L-valine and **PSN-357** was carried out, followed by deprotection to give **27** as a white solid (44% for 2 steps). M.p. 214-216 °C. ^1^H NMR (400 MHz, CD_3_OD): 1.09 (t, *J* = 6.8 Hz, 6H, CH_3_), 1.51-1.75 (m, 2H, CH_2_), 1.87-1.94 (m, 2H, CH_2_), 2.31-2.34 (m, 1H, CH), 3.15-3.22 (m, 2H, PhCH_2_+CH_2_), 3.38-3.53 (m, 2H, CH_2_), 3.59-3.85 (m, 2H, PhCH_2_+CH_2_), 3.94-3.97 (m, 1H, CH), 5.10-5.12 (m, 1H, CH), 5.35 (t, *J* = 7.6 Hz, 1H, CH), 7.02-7.08 (m, 2H, ArH+Pyrrole-H), 7.33-7.38 (m, 2H, ArH), 7.44 (d, *J* = 4.4 Hz, 1H, ArH), 8.14 (s, 1H, Py-H), 8.93 (s, 1H, Py-H). ^13^C NMR (100 MHz, CD_3_OD): 169.90 (C=O), 169.87 (C=O of isomer), 167.9 (C=O), 168.0 (C=O of isomer), 162.0 (d, *J* = 242.6 Hz, ArC), 159.3 (C=O), 141.7 (ArC), 141.6 (ArC of isomer), 138.13 (ArC), 138.10 (ArC of isomer), 132.96 (ArC), 133.05 (ArC of isomer), 132.60 (d, *J* =2.8 Hz, ArC), 132.58 (d, *J* =2.9 Hz, ArC of isomer), 131.20 (ArC), 131.26 (ArC of isomer), 131.1(ArC), 131.04 (ArC), 130.96 (ArC of isomer), 117.6 (ArC), 115.0 (d, *J* = 7.3 Hz, ArC), 114.8 (d, *J* = 7.2 Hz, ArC), 102.92 (ArC), 102.89 (ArC of isomer), 71.5 (CH), 71.7 (CH of isomer), 50.8 (CH), 50.9 (CH of isomer), 48.5 (CH), 42.6 (CH_2_), 39.0 (CH_2_), 39.1 (CH_2_ of isomer), 36.9 (PhCH_2_), 36.8 (PhCH_2_ of isomer),30.3 (CH_2_), 30.4(CH_2_ of isomer), 29.7 (CH_2_), 29.8 (CH_2_ of isomer), 29.6 (CH), 17.1 (CH_3_), 16.9 (CH_3_). MS (ESI) *m/z*: 544.1 [*M*+H]^+^.

**(*S*)-N-(1-Isopropyl-2-{[(S)*-*2-(5-chloro-1*H*-pyrrolo[2,3-c]pyridine-2-carboxamido)-2-(4-fluorobenzyl)-1-oxoethyl]-piperidin-4-yloxy}-2-oxoethyl)-3*α*,7*α*,12*α*-trihydroxy-5*β*-cholan-24-amide (4).** Following the procedure for preparation of **3**, the amidation reaction of cholic acid with **27** was carried out to give **4** as a white solid (29.2%). m.p. 185-187 °C. ^1^H NMR (400 MHz, CD_3_OD): 0.68 (d, *J* = 36.4 Hz, 3H, CH_3_), 0.88 (d, *J* = 24.0 Hz, 3H, CH_3_), 0.95-1.01 (m, 9H, CH_3_), 3.09-3.14 (m, 1H, CH), 3.20 (dd, *J* = 12.8, 8.4 Hz, 1H, CH), 3.39-3.49 (m, 1H, CH), 3.52-3.60 (m, 2H, PhCH_2_+CH_2_), 3.70-3.81 (m, 3H, CH_2_), 3.90-3.97 (m, 1H, PhCH_2_), 4.25 (dd, *J* = 15.6, 6.0 Hz, 1H, CH), 4.94-4.99 (m, 1H, CH), 5.31-5.34 (m, 1H, CH), 7.02-7.07 (m, 2H, ArH+Pyrrole-H), 7.18 (d, *J* = 2.8 Hz, 1H, ArH), 7.31-7.36 (m, 2H, ArH), 7.70 (s, 1H, Py-H), 8.59 (d, *J* = 2.4 Hz, 1H, Py-H). ^13^C NMR (10`0 MHz, CD_3_OD): 175.7 (C=O), 175.8 (C=O of isomer), 171.1 (C=O), 171.0 (C=O of isomer), 170.0 (C=O), 169.98 (C=O of isomer),162.0 (d, *J* = 241.4 Hz, ArC), 160.54 (C=O), 160.45 (C=O of isomer), 139.5 (ArC), 136.51 (ArC), 136.47 (ArC of isomer), 135.3 (ArC）, 134.48 (ArC), 134.46 (ArC of isomer), 132.68 (ArC), 132.71 (ArC of isomer), 132.65 (ArC), 132.61 (ArC of isomer), 131.1 (d, *J* = 6.8 Hz, ArC), 131.0 (d, *J* = 7.4 Hz, ArC of isomer), 115.4 (ArC), 115.3 (ArC of isomer), 115.0 (d, *J* = 4.4 Hz, ArC), 114.8 (d, *J* = 4.5 Hz, ArC), 101.7 (ArC), 72.63, 72.61 (from isomer), 71.5, 69.5, 69.4 (from isomer) and 67.64, 67.61 (from isomer) (CH-OH of the cholic acid residues and CH-O of piperidine), 58.3 (CH), 58.1 (CH of isomer), 50.6 (CH ), 50.5 (CH of isomer), 48.5, 46.7 and 46.0 (C and CH of the cholic acid residues), 42.5 (CH_2_), 41.8, 41.6, 41.5 (from isomer), 39.6, 39.5 (from isomer) and 39.1 (CH_2_ and CH of the cholic acid residues), 39.0 (CH_2_), 38.98 (CH_2_ of isomer), 37.0 (PhCH_2_), 35.5, 35.09, 35.06 (from isomer), 34.5, 34.4 (from isomer), 32.3,32.2 (from isomer) and 31.9 (CH_2_ and C of the cholic acid residues), 30.4, 30.3 (from isomer), 30.0, 30.1 (from isomer), 29.8, 28.2, 28.1 (from isomer), 27.3, 26.5, 26.4 (from isomer) and 22.85, 22.80 (from isomer)(CH_2_ and CH of the cholic acid residues), 21.81, 21.79 (from isomer), 18.2, 17.35, 17.30 (from isomer), 16.4, 16.3 (from isomer), 11.7, 11.6 (from isomer) (CH_3_ of the cholic acid residues and CH_3_ of the isopropyl). MS (ESI) *m/z*: 934.3 [*M*+H]^+^. HRMS (ESI) [M+Na]^+^ m/z: 956.4711 (calcd for C_51_H_69_ClFN_5_NaO_8_, 956.4716).

**(*S*)-1-[(*S*)-2-(5-Chloro-1H-pyrrolo[2,3-c]pyridine-2-carboxamido)-2-(4-fluorobenzyl)-1-oxoethyl]-piperidine-4-yl 2-amino-3-hydroxypropionate (28).** To a solution of N-(tert-butoxycarbonyl)-L-serine (0.19 g, 0.63 mmol) and imidazole (0.17 g, 2.52 mmol) in DMF (5 mL) was added TBDMSCl (0.19 g, 1.26 mmol). The reaction mixture was stirred at room temperature over night and then diluted with water (20 mL) and extracted with ethyl acetate (10 mL×3). The organic layers was washed with brine (10 mL), dried over Na_2_SO_4_, filtered, and concentrated to give a light yellow solid, which was used for next reaction without further purification. Following the procedure for preparation of **26**, the esterification reaction between the product obtained through the above step and **PSN-357** was carried out, followed by deprotection to give **28** as a white solid (70% for 2 steps). m.p. 203-205 °C. ^1^H NMR (400 MHz, CD_3_OD): 1.07-1.67 (m, 2H, CH_2_), 1.76-1.87 (m, 2H, CH_2_), 3.06-3.21 (m, 2H, PhCH_2_+CH_2_), 3.34-3.51 (m, 1H, CH_2_), 3.52-3.60 (m, 2H, CH_2_), 3.64-3.83 (m, 4H, CH+PhCH_2_+CH_2_), 4.96-5.01 (m, 1H, CH), 5.31 (t, *J* = 7.2 Hz, 1H, CH), 7.02 (td, *J* = 8.4, 2.4 Hz, 2H, ArH+Pyrrole-H), 7.16 (s, 1H, ArH), 7.31 (t, *J* = 7.6 Hz, 2H, ArH), 7.68 (s, 1H, Py-H), 8.57 (s, 1H, Py-H). ^13^C NMR (100 MHz, CD_3_OD): 173.4 (C=O), 171.4 (C=O), 163.5 (d, *J* = 241.4 Hz, ArC), 162.0 (C=O), 140.9 (ArC), 138.0 (ArC), 136.8 (ArC), 135.8 (ArC), 134.1 (ArC), 132.5 (ArC), 132.4 (ArC), 132.3 (ArC), 116.7 (ArC), 116.4 (d, *J* = 4.3 Hz, ArC), 116.1 (d, *J* = 4.5 Hz, ArC), 103.0 (ArC), 71.3 (CH), 71.1 (CH of isomer), 64.6 (CH_2_-OH), 64.5 (CH_2_-OH of isomer), 57.2 (CH), 52.0 (CH), 43.9 (CH_2_), 40.3 (CH_2_), 38.4 (PhCH_2_), 31.7 (CH_2_), 31.1 (CH_2_). MS (ESI) *m/z*: 532.0 [*M*+H]^+^.

**(*S*)-N-(1-Hydroxymethyl-2-{[(*S*)*-*2-(5-chloro-1*H*-pyrrolo[2,3-c]pyridine-2-carboxamido)-2-(4-fluorobenzyl)-1-oxoethyl]-piperidin-4-yloxy}-2-oxoethyl)-3*α*,7*α*,12*α*-trihydroxy-5*β*-cholan-24-amide (5).** Following the procedure for preparation of **3**, the amidation reaction of cholic acid with **27** was carried out to give **5** as a white solid (20%). m.p. 175-177 °C. ^1^H NMR (400 MHz, CD_3_OD): 0.67 (d, *J* = 41.6 Hz, 3H, CH_3_), 0.88 (d, *J* = 28.0 Hz, 3H, CH_3_), 0.99-1.06 (m, 3H, CH_3_), 3.09-3.12 (m, 1H, CH), 3.20 (dd, *J* = 13.2, 8.0 Hz, 1H, CH), 3.37-3.50 (m, 2H, CH+PhCH_2_), 3.61-3.71 (m, 4H, CH_2_), 3.78-3.97 (m, 3H, CH_2_+PhCH_2_), 4.47 (dt, *J* = 17.6, 7.6 Hz, 1H, CH), 4.96-5.03 (m, 1H, CH), 5.34 (q, *J* = 7.6 Hz, 1H, CH), 7.04 (t, *J* = 8.8 Hz, 2H, ArH+Pyrrole-H), 7.17 (d, *J* = 5.6 Hz, 1H, ArH), 7.33 (t, *J* = 6.0 Hz, 2H, ArH), 7.69 (s, 1H, Py-H), 8.60 (br *s*, 1H Py-H). ^13^C NMR (100 MHz, CD_3_OD): 177.0 (C=O), 171.3 (C=O), 171.1 (C=O), 163.4 (d, *J* = 242.6 Hz, ArC), 161.7 (C=O), 161.9 (C=O of isomer), 140.8 (ArC), 137.92 (ArC), 137.87 (ArC of isomer), 136.7 (ArC), 135.83 (ArC), 135.78 (ArC of isomer), 134.05 (d, *J* = 3.0 Hz, ArC), 134.02 (d, *J* = 2.3 Hz, ArC of isomer), 132.42 (ArC), 132.39 (ArC of isomer), 132.34 (ArC), 132.31 (ArC of isomer), 116.7 (d, *J* = 2.9 Hz ArC), 116.4 (ArC), 116.1 (ArC), 103.0 (ArC), 73.99, 73.97 (from isomer), 72.8, 70.9, 71.1 (from isomer) and 69.02, 68.96 (from isomer) (CH-OH of the cholic acid residues and CH-O of piperidine), 62.8 (CH_2_-OH), 62.7 (CH_2_-OH of isomer), 56.5 (CH), 56.4 (CH of isomer), 51.8 (CH), 52.0 (CH of isomer), 47.8, 47.9 (from isomer) and 47.3, 47.4 (from isomer) (C and CH of the cholic acid residues), 43.67 (CH_2_), 43.75 (CH_2_ of isomer), 43.1, 42.8, 42.9 (from isomer), 40.94, 40.89 (from isomer) and 40.4 (CH_2_ and CH of the cholic acid residues), 40.1 (CH_2_), 40.2 (CH_2_ of isomer), 38.4 (PhCH_2_), 38.2 (PhCH_2_ of isomer), 36.7, 36.8 (from isomer), 36.4, 35.79, 35.8 (from isomer), 35.7 (from isomer), 33.5, 33.6 (from isomer), 33.0 and 31.5, 31.6 (from isomer) (CH_2_ and C of the cholic acid residues), 31.1 (CH_2_), 30.99 (CH_2_), 30.94 (CH_2_ of isomer), 29.52, 29.48 (from isomer), 28.7, 28.6 (from isomer), 27.81, 27.76 (from isomer) and 24.1, 24.2 (from isomer) (CH_2_ and CH of the cholic acid residues), 23.14, 23.11 (from isomer), 17.74, 17.68 (from isomer) and 12.99, 12.94 (from isomer) (CH_3_ of the cholic acid residues). MS (ESI) *m/z*: 922.5 [*M*+H]^+^. HRMS (ESI) [M+H]^+^ m/z: 922.4528 (calcd for C_49_H_66_ClFN_5_O_9_, 922.4533).

**(*S*)-1-[(*S*)-2-(5-Chloro-1H-pyrrolo[2,3-c]pyridine-2-carboxamido)-2-(4-fluorobenzyl)-1-oxoethyl]-piperidine-4-yl 2-[(tert-butoxycarbonyl)amino]-4-(tert-butyl)succinate (25).** Following the procedure for preparation of **22**, the esterification reaction between Boc-Asp(OtBu)-OH and **PSN-357** was carried out to give **25** as a white solid (50.8%). m.p. 118-120 °C. ^1^H NMR (400 MHz, CDCl_3_): 1.14-1.33 (m, 1H, CH_2_), 1.41 (t, *J* = 4.0 Hz, 9H, CH_3_), 1.45 (t, *J* = 4.4 Hz, 9H, CH_3_), 1.60-1.91 (m, 3H, CH_2_), 2.73-2.77 (m, 1H, CH_2_), 2.86-2.94 (m, 1H, CH_2_), 3.12-3.15 (m, 2H, PhCH_2_+CH_2_), 3.44-3.49 (m, 1H, CH_2_), 3.57-3.62 (m, 1H, CH_2_), 3.72-3.85 (m, 1H, CH_2_), 4.50-4.61 (m, 1H, PhCH_2_), 4.97-5.07 (m, 1H, CH), 5.32-5.37 (m, 1H, CH), 5.50-5.52 (m, 1H, CH), 6.84 (d, *J* = 7.6 Hz, 1H, NH), 6.95-6.99 (m, 2H, ArH+Pyrrole-H), 7.16-7.20 (m, 2H, ArH), 7.49 (d, *J* = 3.6 Hz, 1H, ArH), 8.03-8.07 (m, 1H, Py-H), 8.58 (d, *J* = 6.4 Hz, 1H, Py-H), 10.38 (d, *J* = 7.2 Hz, 1H, NH), 10.44 (s, 1H, NH). 13C-NMR (100 MHz, CDCl_3_): 170.5 (C=O), 170.3 (C=O), 169.5 (C=O), 162.1 (d, *J* = 244.9 Hz, ArC), 160.1 (C=O), 155.5 (ArC), 140.9 (ArC), 135.2 (ArC), 135.0 (ArC), 134.8 (ArC), 132.4 (ArC), 131.4 (ArC), 131.0 (ArC), 130.9 (ArC), 115.8 (ArC), 115.6 (ArC), 115.4 (ArC), 101.6 (ArC), 81.9 (C), 81.8 (C of isomer), 80.1 (C), 69.5 (CH), 69.6 (CH of isomer), 50.5 (CH), 50.4 (CH of isomer), 50.25 (CH), 50.19 (CH of isomer), 49.5 (CH_2_), 42.6 (CH_2_), 39.2 (CH_2_), 37.8 (PhCH_2_), 30.45 (CH_2_), 30.39 (CH_2_ of isomer), 30.0 (CH_2_), 29.9 (CH_2_ of isomer), 28.33 (CH_3_), 28.30 (CH_3_ of isomer), 28.02 (CH_3_), 28.06 (CH_3_ of isomer). MS (ESI) m/z: 716.2 [*M*+H]^+^.

**(*S*)-N-(1-Carboxymethyl-2-{[(S)*-*2-(5-chloro-1*H*-pyrrolo[2,3-c]pyridine-2-carboxamido)-2-(4-fluorobenzyl)-1-oxoethyl]-piperidin-4-yloxy}-2-oxoethyl)-3*α*,7*α*,12*α*-trihydroxy-5*β*-cholan-24-amide (6).** To a solution of **25** (170 mg, 0.24 mmol) in EtOAc (10 mL) was added dropwise a solution of HCl in EtOAc (4N, 3 mL) at 0 °C. The reaction mixture stirred at room temperature for 2h and then concentrated in vacuo to give **29** as a white solid, which was used for the next reaction without further purification (Crude Yield, 67.2%). Then following the procedure for preparation of **3**, the amidation reaction of cholic acid with **29** was carried out to give **6** as a white solid (15.8%). M.p. 187-189 °C. ^1^H NMR (400 MHz, CD_3_OD): 0.66 (d, *J* = 42.0 Hz, 3H, CH_3_), 0.89 (d, *J* = 30.4 Hz, 3H, CH_3_), 1.01 (dd, *J* = 20.8, 6.4Hz, 3H, CH_3_), 2.83-2.87 (m, 2H, CH_2_), 3.16 (ddd, *J* = 21.2, 13.6, 8.0 Hz, 2H, CH), 3.37-3.42 (m, 1H, CH), 3.50-3.81 (m, 5H, PhCH_2_+CH_2_), 3.90-3.97 (m, 1H, PhCH_2_), 4.69-4.73 (m, 1H, CH), 5.01-5.03 (m, 1H, CH), 5.34 (q, *J* = 8.4 Hz, 1H, CH), 7.04 (t, *J* = 8.4 Hz, 2H, ArH+Pyrrole-H), 7.24 (d, *J* = 5.6 Hz, 1H, ArH), 7.33 (dd, *J* = 8.4, 5.6 Hz, 2H, ArH), 7.81 (s, 1H, Py-H), 8.11 (s, 1H, Py-H), 8.67 (br *s*, 1H, NH). ^13^C NMR (100 MHz, CD_3_OD): 174.7 (C=O), 174.8 (C=O of isomer), 172.0 (C=O), 171.9 (C=O of isomer), 169.68 (C=O), 169.67 (C=O of isomer), 169.31 (C=O), 169.30 (C=O of isomer), 161.5 (d, *J* = 242.4 Hz, ArC), 159.3 (C=O), 159.4 (C=O of isomer), 138.48 (ArC), 138.55 (ArC of isomer), 136.21 (ArC), 136.17 (ArC of isomer), 135.9 (ArC), 135.8 (ArC of isomer), 132.4 (ArC), 132.3 (ArC of isomer), 132.14 (d, *J* = 3.2 Hz, ArC), 132.11 (d, *J* = 3.5 Hz, ArC of isomer), 131.5 (ArC), 130.6 (ArC), 130.5 (ArC), 130.4 (ArC of isomer), 116.0 (ArC), 114.5 (ArC), 114.3 (ArC), 101.7 (ArC), 72.11, 72.15 (from isomer), 70.93, 70.97 (from isomer), 69.2, 69.1 (from isomer) and 67.19, 67.14 (from isomer) (CH-OH of the cholic acid residues and CH-O of piperidine), 50.3 (CH), 50.0 (CH of isomer), 48.70 (CH), 48.73 (CH of isomer), 46.2 and 45.5, 45.6 (from isomer) (C and CH of the cholic acid residues), 41.84 (CH_2_), 41.76 (CH_2_ of isomer), 41.2, 41.3 (from isomer), 41.0, 41.1 (from isomer), 39.1, 39.0 (from isomer)and 38.55, 38.52 (from isomer) (CH_2_ and CH of the cholic acid residues), 38.4 (PhCH_2_), 38.3 (PhCH_2_ of isomer), 36.5, 36.3 (from isomer), 34.96, 34.94 (from isomer), 34.84, 34.89 (from isomer), 34.5, 34.6 (from isomer) (CH_2_ and C of the cholic acid residues), 33.93 (CH_2_), 33.99 (CH_2_ of isomer), 33.86 (CH_2_ of isomer), 31.8, 31.2 and 29.6 (CH_2_ of the cholic acid residues), 29.3(CH_2_), 29.0, 27.6, 27.7 (from isomer), 26.8, 25.9, 26.0 (from isomer) and 22.35, 22.28 (from isomer) (CH_2_ and CH of the cholic acid residues), 21.2, 21.3 (from isomer), 15.75, 15.86 (from isomer) and 11.109, 11.15 (from isomer) (CH_3_ of the cholic acid residues). MS (ESI) *m/z*: 950.0 [*M*+H]^+^. HRMS (ESI) [M+H]^+^ m/z: 950.4477 (calcd for C_50_H_66_ClFN_5_O_10_, 950.4482).

**(S)-Methyl 2-[(S)-2-(tert-butoxycarbonyl)amino-3-methylbutanamido] propionate (31).** N-(tert-butoxycarbonyl)-L-valine (**30**, 0.10 g, 0.46 mmol) was dissolved in 5 mL of anhydrous DMF, to which EDCI (132.5 mg, 0.69 mmol), HOBt (93.3 mg, 0.69 mmol), and DIPEA (0.24 mL, 1.38 mmol) were added at 0 °C. After the mixture was stirred for 10 min at room temperature, L-alanine methyl ester hydrochloride (64.5 mg, 0.46 mmol) was added. The mixture was stirred at room temperature over night and then diluted with water (10 mL) and extracted with EtOAc (10 mL×3). The combined organic layers were washed with brine, dried over Na_2_SO_4_, filtered, and concentrated in vacuo. The crude product was purified by column chromatography over silica gel [petroleum ether-EtOAc (8:1)] to give **31** as a white solid (97.0 mg, 69.8%). m.p. 142-144 °C. ^1^H NMR (400 MHz, CDCl_3_): 0.92 (d, *J* = 6.8 Hz, 3H, CH_3_), 0.97 (d, *J* = 6.8 Hz, 3H, CH_3_), 1.41 (d, *J* = 7.2 Hz, 3H, CH_3_), 1.45 (s, 9H, CH_3_), 2.09-2.17 (m, 1H, CH), 3.75 (s, 3H, CH_3_), 3.90-3.94 (m, 1H, CH), 4.55-4.62 (m, 1H, CH), 5.05 (d, *J* = 6.8 Hz, 1H, NH), 6.38 (d, *J* = 4.4 Hz, 1H, NH). ^13^C-NMR (100 MHz, CDCl_3_): 173.1 (C=O), 171.1 (C=O), 155.8 (C=O), 79.9 ((CH_3_)_3_CO), 59.8 (CH), 52.4 (CH_3_), 48.0 (CH), 31.0 (CH), 28.3 (CH_3_), 19.2 (CH_3_), 18.3 (CH_3_), 17.7 (CH_3_). MS (ESI) *m/z*: 325.2 [*M*+Na]^+^.

**(S)-2-[(S)-2-(tert-Butoxycarbonyl)amino-3-methylbutanamido]propanoic acid (32).** To a solution of **31** (2.5 g, 8.3 mmol) in MeOH/H_2_O (1:1, 20 mL), NaOH (0.5 g, 12.5 mmol mmol) was added. The reaction mixture stirred at room temperature for 3h and then concentrated in vacuo. The residue was redissolved in H_2_O and washed with EtOAc (20mL×3), then the aqueous was adjusted to pH = 2~3 with HCl aqueous (1 M) and the solid was participated. The precipitate was filtered, washed with water, and dried to give **32**, which was almost a pure product, and was used for the next reaction without further purification. Crystallization of the crude product from ethanol gave **32** as a white solid (1.6 g, 66.9%). m.p.152-154 °C. ^1^H NMR (400 MHz, CDCl_3_): 0.95 (d, *J* = 6.8 Hz, 3H, CH_3_), 0.98 (d, *J* = 6.4 Hz, 3H, CH_3_), 1.47 (s, 9H, CH_3_), 1.48 (d, *J* = 7.2 Hz, 3H, CH_3_), 2.08-2.12 (m, 1H, CH), 4.01 (t, *J* = 7.6 Hz, 1H, CH), 4.61 (t, *J* = 6.4 Hz, 1H, CH), 5.30 (d, *J* =9.2 Hz, 1H, NH), 6.88 (s, 1H, NH). ^13^C-NMR (100 MHz, CDCl_3_): 175.6 (C=O), 171.9 (C=O), 156.2 (C=O), 80.3 ((CH_3_)_3_CO), 59.9 (CH), 48.1 (CH), 31.0 (CH), 28.3(CH_3_), 19.1 (CH_3_), 18.0 (CH_3_), 17.9 (CH_3_). MS (ESI) *m/z*: 287.1 [*M*-H]^-^.

**(*S*)-1-[(*S*)-2-(5-Chloro-1H-pyrrolo[2,3-c]pyridine-2-carboxamido)-2-(4-fluorobenzyl)-1-oxoethyl]-piperidine-4-yl 2-{(S)-2-[(tert-butoxycarbonyl)amino]-3-methylbutanamido}propionate (33).** Following the procedure for preparation of **22**, the esterification reaction between **32** and **PSN-357** was carried out to give **33** as a white solid (52%). m.p.151-153 °C. ^1^H NMR (400 MHz, CD_3_OD): ^1^H NMR (400 MHz, CD_3_OD): 0.89-1.00 (m, 6H, CH_3_), 1.39-1.45 (m, 12H, CH_3_), 1.55-1.87 (m, 4H, CH_2_), 2.00-2.04 (m, 1H, CH), 3.10-3.20 (m, 2H, PhCH_2_+CH_2_), 3.40-3.57 (m, 2H, CH_2_), 3.64-3.85 (m, 2H, PhCH_2_+CH_2_), 3.89-3.95 (m, 1H, CH), 4.36-4.42 (m, 1H, CH), 4.92-4.96 (m, 1H, CH), 5.31-5.35 (m, 1H, CH), 7.00-7.06 (m, 2H, ArH+Pyrrole-H), 7.14 (s, 1H, ArH), 7.30-7.35 (m, 2H, ArH), 7.64 (s, 1H, Py-H), 8.55 (s, 1H, Py-H). ^13^C NMR (100 MHz, CD_3_OD): 172.9 (C=O), 172.8 (C=O of isomer), 171.8 (C=O), 171.7 (C=O of isomer), 170.0 (C=O), 169.9 (C=O of isomer), 162.0 (d, *J* = 242.4 Hz, ArC), 160.53 (C=O), 160.46 (C=O of isomer), 156.5 (C=O), 156.4 (C=O of isomer), 139.5 (ArC), 136.5 (ArC), 135.3 (ArC), 134.4 (ArC), 132.74 (ArC), 132.71 (ArC of isomer), 132.65 (ArC), 132.69 (ArC of isomer), 131.0 (d, *J* = 5.0 Hz, ArC), 130.9 (d, *J* = 5.2 Hz, ArC), 115.3 (ArC), 114.9 (d, *J* = 8.4 Hz, ArC), 114.7 (d, *J* =8.3 Hz, ArC), 101.6 (ArC), 79.1 (C), 69.9 (CH), 69.7 (CH of isomer), 59.7 (CH), 60.0 (CH of isomer), 50.6 (CH), 42.5 (CH), 42.6 (CH of isomer), 39.0 (CH_2_), 39.1 (CH_2_ of isomer), 37.0 (CH_2_), 36.9 (CH_2_ of isomer), 30.8 (PhCH_2_), 30.2 (CH_2_), 30.4 (CH_2_ of isomer), 29.6 (CH_2_), 29.8 (CH_2_ of isomer), 27.3 (CH_3_), 18.38 (CH_3_), 18.42 (CH_3_ of isomer), 17.1 (CH_3_), 17.0 (CH_3_ of isomer), 16.0 (CH_3_). MS (ESI) *m/z*: 715.0 [*M*+H]^+^.

**(*S*)-N-{1-Isopropyl-2-[(1-oxo-1-{[(*S*)*-*2-(5-chloro-1*H*-pyrrolo[2,3-c]pyridine-2-carboxamido)-2-(4-fluorobenzyl)-1-oxoethyl]-piperidin-4-yloxy}peopan-2-yl)amino]-2-oxoethyl}-3*α*,7*α*,12*α*-trihydroxy-5*β*-cholan-24-amide (7).** To a solution of **33** (1.0 g, 1.4 mmol) in anhydrous CH_2_Cl_2_ (10 mL) was added TFA (5 mL, 70 mmol), the reaction mixture was stirred at 0 °C for 2 h. Then the reaction mixture was concentrated in vacuo. The residue was redissolved in EtOAc (20 mL) and washed with saturated NaHCO_3_ (20 mL) and brine (20 mL), dried over Na_2_SO_4_, filtered, and concentrated to give crude **34** as a white solid, which was used for next reaction without further purification. Cholic acid (**17**, 330 mg, 0.80 mmol) was dissolved in 5 mL of anhydrous DMF, to which HATU (456 mg, 1.2 mmol), Et_3_N (166 µL, 1.2 mmol) were added at 0 °C. After the mixture was stirred for 10 min at room temperature, crude **34** (450 mg, crude) was added. The mixture was stirred at room temperature over night and then concentrated in vacuo. High degree of purity for **7** as a light yellow solid was obtained using preparative-reverse phase HPLC (150 mg, 10.7% for 2 steps). m.p.120-122 °C. ^1^H NMR (400 MHz, CD_3_OD): 0.65-0.72 (m, 3H, CH_3_), 0.87-1.04 (m, 15H, CH_3_), 3.07-3.22 (m, 2H, CH), 3.37-3.48 (m, 2H, CH+PhCH_2_), 3.55-3.62 (m, 2H, CH_2_), 3.75-3.81 (m, 2H, CH_2_), 3.90-3.96 (m, 1H, PhCH_2_), 4.14-4.27 (m, 1H, CH), 4.31-4.40 (m, 1H, CH), 4.96-5.00 (m, 1H, CH), 5.31-5.35 (m, 1H, CH), 7.01-7.07 (m, 2H, ArH+Pyrrole-H), 7.19 (t, *J* = 4.8 Hz, 1H, ArH), 7.31-7.36 (m, 2H, ArH), 7.71 (s, 1H, Py-H), 8.60 (s, 1H, Py-H). ^13^C NMR (100 MHz, CD_3_OD): 176.80 (C=O), 176.77 (C=O of isomer), 173.7 (C=O), 173.6 (C=O of isomer), 173.2 (C=O), 171.3 (C=O), 171.2 (C=O of isomer), 163.4 (d, *J* = 244.3 Hz, ArC), 161.9 (C=O), 161.8 (C=O of isomer), 140.9 (ArC), 138.0 (ArC), 136.8 (ArC), 135.8 (ArC), 135.9 (ArC of isomer), 134.2 (ArC), 132.4 (d, *J* = 6.0 Hz, ArC), 132.3 (d, *J* = 6.0 Hz, ArC of isomer), 116.7 (ArC), 116.3 (d, *J* = 8.6 Hz, ArC), 116.1 (d, *J* = 8.5 Hz, ArC), 103.1 (ArC), 74.01, 74.04 (from isomer), 72.9, 71.3, 71.1 (from isomer) and 69.1 (CH-OH of the cholic acid residues and CH-O of piperidine), 59.9 (CH), 52.0 (CH), 48.1 (CH), 47.52, 47.50 (from isomer), 43.9 and 43.2 (C and CH of the cholic acid residues), 43.02 (CH), 42.98 (CH of isomer), 41.0, 40.5 and 40.4 (CH_2_ and CH of the cholic acid residues), 38.41 (CH_2_), 38.43 (CH_2_ of isomer), 36.9 (CH_2_), 36.5, 35.9, 35.8, 34.0, 33.3, 32.0, 31.9 (from isomer), 31.7, 31.6 (from isomer) (CH_2_ , CH and C of the cholic acid residues), 31.2 (PhCH_2_), 29.60, 29.57 (from isomer), 28.72, 28.70 (from isomer), 27.9 and 24.2 (CH_2_ , CH and C of the cholic acid residues), 23.2 (CH_3_ of the cholic acid residues), 19.81 (CH_3_), 19.76 (CH_3_ of isomer), 18.9 (CH_3_), 17.8 (CH_3_ of the cholic acid residues), 17.33 (CH_3_), 17.27 (CH_3_ of isomer), 13.01, 13.03 (from isomer)(CH_3_ of the cholic acid residues). MS (ESI) *m/z*: 1005.0 [*M*+H]^+^. HRMS (ESI) [M+Na]^+^ m/z: 1027.5054 (calcd for C_54_H_74_ClFN_6_NaO_9_, 1004.5190).

**(*S*)-Methyl 6-benzyloxycarbonylamino-2-[(*S*)-2-tert-butoxycarbonylamino-3-phenylpropanamido]hexanoate (36).** Following the procedure for preparation of **31**, the amidation reaction of N6-Cbz-L-lysine methyl **35** with Boc-L-phenylalanine ester was carried out to give **36** as a white solid (73%). M.p. 84-86 °C. ^1^H NMR (400 MHz, CDCl_3_): 1.24-1.33 (m, 2H, CH_2_), 1.42 (s, 9H, (CH_3_)_3_C), 1.51-1.56 (m, 1H, CH_2_), 1.62-1.70 (m, 2H, CH_2_), 1.79-1.88(m, 1H, CH_2_), 3.07 (t, *J* = 6.0 Hz, 2H, CH_2_), 3.16-3.22 (m, 2H, CH_2_), 3.72 (s, 3H, CH_3_), 4.35-4.40 (m, 1H, CH), 4.54-4.59 (m, 1H, CH), 4.96 (br s, 1H, NH), 5.08-5.15 (m, 2H, CH_2_), 6.49 (d, *J* = 7.6 Hz, 1H, ArH), 7.20-7.28 (m, 4H, ArH), 7.30-7.38 (m, 6H, ArH). ^13^C NMR (100 MHz, CDCl_3_): 172.2 (C=O), 171.4 (C=O), 156.5 (C=O), 155.5 (C=O), 136.6 (ArC), 129.3 (ArC), 128.6 (ArC), 128.5 (ArC), 128.1 (ArC), 128.0 (ArC), 126.9 (ArC), 80.2 (C), 66.6 (CH_2_), 55.7 (CH), 52.4 (CH), 52.0 (CH_3_), 40.5 (CH_2_), 38.2 (PhCH_2_), 31.9 (CH_2_), 29.2 (CH_2_), 28.3 (CH_3_), 22.2 (CH_2_).MS (ESI) *m/z*: 542.3 [*M*+H]^+^.

**(*S*)-6-Benzyloxycarbonylamino-2-[(*S*)-2-tert-butoxycarbonylamino-3-phenylpropanamido]hexanoic acid (37).** Following the procedure for preparation of **32**, the hydrolysis reaction of **36** with NaOH was carried out to give **37** as a white solid (79%). m.p. 53-55 °C. ^1^H NMR (400 MHz, CDCl_3_): 1.24-1.38 (m, 11H, CH_3_+CH_2_), 1.49-1.51 (m, 2H, CH_2_), 1.73-1.88 (m, 2H, CH_2_), 2.98-3.18 (m, 4H, PhCH_2_+CH_2_), 4.49-4.55 (m, 2H, CH+CH), 5.06-5.17 (m, 2H, PhCH_2_), 5.27-5.37 (m, 1H, NH), 7.04 (s, 1H, NH), 7.17-7.27 (m, 5H, ArH), 7.30-7.35 (m, 5H, ArH). ^13^C-NMR (100 MHz, CDCl_3_): 174.5 (C=O), 171.9 (C=O), 156.8 (C=O), 155.7 (C=O), 136.5 (ArC), 129.3 (ArC), 128.6 (ArC), 128.5 (ArC), 128.1 (ArC), 128.0 (ArC), 126.9 (ArC), 80.4 ((CH_3_)_3_CO), 66.8 (PhCH_2_O), 55.6 (CH), 52.1 (CH), 40.6 (CH_2_), 38.2 (PhCH_2_), 31.5 (CH_2_), 29.2 (CH_2_), 28.2 (CH_2_), 22.1 (CH_2_). MS (ESI) *m/z*: 526.0 [*M*-H]^-^.

**(S)-2-[(S)-2-tert-Butoxycarbonylamino-3-phenylpropanamido]-6-({[(9H-fluoren-9-yl)methoxy]carbonyl}amino)hexanoate (40).** A mixture of **37** (1 g, 1.9 mmol) and 10% Pd/C (0.1 g) in MeOH (10 mL) was stirred at room temperature under H_2_ at atmospheric pressure for 18 h. The reaction mixture was filtered through Celite, and the insoluble substance was washed with MeOH (10 mL × 3). The filtrate was concentrated in vacuo to give **38**, which was used in the next step without further purification. **38** was dissolved in a 1:1 mixture of dioxane and a saturated aqueous NaHCO_3_ solution (20 mL) and cooled to 0 °C. FmocCl (517 mg, 2.0 mmol) was slowly added, and the mixture was stirred at 0 °C for 5h and then concentrated in vacuo. The residue was redissolved in H_2_O and then adjusted to pH = 2~3 with HCl aqueous (1 M) and the solid was participated. The precipitate was filtered, washed with water, dried over Na_2_SO_4_, filtered, and concentrated in vacuo to afford **39** as a crude white solid.

To a mixture of crude **39** (380 mg, 0.6 mmol) and **PSN-357** (266 mg, 0.6 mmol) in anhydrous CH_2_Cl_2_ (30 mL) under ice bath, T_3_P (50 wt.% soln. in ethyl acetate, 0.95 mL, 3.1 mmol) was added dropwise. The mixture was stirred at room temperature over night under a nitrogen atmosphere and then concentrated in vacuo. The residue was redissolved in EtOAc (30 mL) and washed with brine (30 mL×3), dried over Na_2_SO_4_, filtered, and concentrated in vacuo. High degree of purity for **40** as a white solid was obtained using preparative-reverse phase HPLC (380 mg, 19.2% for 3 steps). m.p.124-126 °C. ^1^H NMR (400 MHz, CD_3_OD): 1.32-1.37 (m, 11H, CH_3_+CH_2_), 1.48-1.85 (m, 8H, CH_2_), 2.77-2.86 (m, 1H, PhCH_2_), 3.03-3.19 (m, 5H, PhCH_2_+CH_2_), 3.41-3.77 (m, 4H, CH_2_+PhCH_2_), 4.15- 4.20 (m, 1H, CH), 4.31-4.38 (m, 4H, CH+CH_2_), 4.92-4.95 (m, 1H, CH), 5.25-5.33 (m, 1H, CH), 6.97-7.03 (m, 2H, ArH+Pyrrole-H), 7.12-7.31 (m, 10H, ArH), 7.34-7.39 (m, 2H, ArH), 7.60-7.66 (m, 3H, ArH), 7.74-7.79 (m, 2H, ArH+Py-H), 8.55 (d, *J* = 7.6 Hz, 1H, Py-H ). ^13^C NMR (100 MHz, CD_3_OD): 173.2 (C=O), 173.1 (C=O of isomer), 171.1 (C=O), 169.9 (C=O), 162.0 (d, *J* = 243.0 Hz, ArC), 160.5 (C=O), 160.4 (C=O of isomer), 157.5 (C=O), 156.2 (C=O), 153.2 (ArC), 148.7 (ArC), 143.94 (ArC), 143.92 (ArC of isomer), 141.18 (ArC), 141.17 (ArC of isomer), 139.5 (ArC), 139.4 (ArC of isomer), 137.2 (ArC), 137.1 (ArC of isomer), 136.51 (ArC), 136.49 (ArC of isomer), 135.3 (ArC), 134.4 (ArC), 132.7 (ArC), 131.0 (ArC), 130.9 (ArC), 129.00 (ArC), 128.96 (ArC of isomer), 128.01 (ArC), 127.98 (ArC of isomer), 127.3 (ArC), 127.4 (ArC of isomer), 126.74 (ArC), 126.72 (ArC of isomer), 126.3 (ArC), 124.8 (ArC), 119.54 (ArC), 119.51 (ArC of isomer), 115.3 (ArC), 114.9 (d, *J* = 5.5 Hz, ArC), 114.7 (d, *J* = 5.5 Hz, ArC), 101.6 (ArC), 79.2 (C), 69.7 (CH), 69.9 (CH of isomer), 66.24 (CH_2_), 66.16 (CH_2_ of isomer), 55.71 (CH), 55.67 (CH of isomer), 52.5 (CH), 50.6 (CH), 50.5 (CH of isomer), 42.4 (CH), 42.5 (CH of isomer), 39.96 (CH_2_), 39.92 (CH_2_ of isomer), 39.03 (CH_2_), 38.95 (CH_2_ of isomer), 37.9 (CH_2_), 37.8 (CH_2_ of isomer), 36.90 (PhCH_2_), 36.97 (PhCH_2_ of isomer), 30.7 (PhCH_2_)), 30.3 (CH_2_), 29.6 (CH_2_), 29.0 (CH_2_), 27.3 (CH_3_), 27.1 (CH_2_), 22.5 (CH_2_). MS (ESI) *m/z*: 1042.4 [*M*+H]^+^.

**(S)-N-(1-Benzyl-2-{[1-oxo-1-{[(S)*-*2-(5-chloro-1*H*-pyrrolo[2,3-c]pyridine-2-carboxamido)-2-(4-fluorobenzyl)-1-oxoethyl]-piperidin-4-yloxy}-6-({[(9H-fluoren-9-yl)methoxy]carbonyl}amino)hexan-2-yl]amino}-2-oxoethyl)-3*α*, 7*α*, 12*α*-trihydroxy-5*β*-cholan-24-amide (42).** Following the procedure for preparation of **34**, deprotection of **40** with TFA afforded **41** as a white solid. Then following the procedure for preparation of **7**, the amidation reaction of cholic acid with **41** was carried out to give **42** as a white solid (48.5%). m.p.149-151 °C. ^1^H NMR (400 MHz, CD_3_OD): 0.60-0.68 (m, 3H, CH_3_), 0.89-0.99 (m, 9H, CH_3_+CH_2_+CH), 2.71-2.91 (m, 2H, CH), 3.05-3.19 (m, 4H, CH+CH_2_+PhCH_2_), 3.39-3.77 (m, 7H, CH_2_+PhCH_2_), 3.86-3.94 (m, 1H, PhCH_2_), 4.16-4.22 (m, 1H, CH), 4.29-4.40 (m, 2H, CH_2_), 4.65-4.71 (m, 1H, CH), 4.95-4.99 (m, 1H, CH), 5.26-5.36 (m, 1H, CH), 7.00-7.03 (m, 2H, ArH+Pyrrole-H), 7.14-7.40 (m, 12H, ArH), 7.62-7.81 (m, 5H, ArH+Py-H), 8.57 (d, *J* = 7.6 Hz, 1H, Py-H). ^13^C NMR (100 MHz, CD_3_OD): 175.3 (C=O), 172.5 (C=O), 171.1 (C=O), 169.9 (C=O), 162.0 (d, *J* = 241.4 Hz, ArC), 160.5 (C=O), 160.4 (C=O of isomer), 157.5 (C=O), 143.93 (ArC), 143.96 (ArC of isomer), 141.2 (ArC), 140.0 (ArC), 139.5 (ArC), 137.9 (ArC), 137.0 (ArC), 137.1 (ArC of isomer), 136.54 (ArC), 136.52 (ArC of isomer), 135.4 (ArC), 134.45 (ArC), 134.44 (ArC of isomer), 132.7 (ArC), 130.9 (ArC), 131.0 (ArC of isomer), 128.92 (ArC), 128.97 (ArC of isomer), 128.5 (ArC), 128.01 (ArC), 128.05 (ArC of isomer), 127.4 (ArC), 126.8 (ArC), 126.76 (ArC), 126.73 (ArC of isomer), 126.4 (ArC), 124.8 (ArC), 120.6 (ArC), 119.51 (ArC), 119.55 (ArC of isomer), 119.3 (ArC), 115.33 (ArC), 115.31 (ArC of isomer), 115.0 (d, *J* = 3.2 Hz, ArC), 114.8 (d, *J* = 2.2 Hz, ArC), 106.8 (ArC), 101.64 (ArC), 101.62 (ArC of isomer), 72.6, 71.5, 69.8 and 67.6 (CH-OH of the cholic acid residues and CH-O of piperidine), 66.2 (CH_2_), 66.3 (CH_2_ of isomer), 54.3 (CH), 52.6 (CH), 50.6 (CH), 48.4 (CH), 46.6, 46.04, 46.02 (from isomer), 42.4, 42.5 (from isomer), 41.8 and 41.6, 41.5 (from isomer) (C , CH and CH_2_ of the cholic acid residues), 39.93 (CH_2_), 39.94 (CH_2_ of isomer), 39.6 and 39.1 (CH and CH_2_ of the cholic acid residues), 37.4 (CH_2_), 37.0 (PhCH_2_), 35.4, 35.1, 34.5, 32.5 and 31.8 (C , CH and CH_2_ of the cholic acid residues), 30.7 (PhCH_2_), 30.3 (CH_2_), 29.8 (CH_2_), 28.98 (CH_2_), 28.93 (CH_2_ of isomer), 28.2, 27.25, 27.21 (from isomer), 26.5, 22.8 and 22.5 (CH and CH_2_ of the cholic acid residues), 21.8, 16.3 and 11.64, 11.61 (from isomer) (CH_3_ of the cholic acid residues). MS (ESI) *m/z*: 666.6 [*M*/2+H]^+^.

**(S)-N-{1-Benzyl-2-[(1-oxo-1-{[(S)*-*2-(5-chloro-1*H*-pyrrolo[2,3-c]pyridine-2-carboxamido)-2-(4-fluorobenzyl)-1-oxoethyl]-piperidin-4-yloxy}-6-aminohexan-2-yl)amino]-2-oxoethyl}-3*α*,7*α*,12*α*-trihydroxy-5*β*-cholan-24-amide (8).** To a solution of **42** (260 mg, 0.20 mmol) in CH_3_CN (5 mL) was added piperidine (0.6 mL, 6.1 mmol) dropwise at 0 °C. The reaction mixture stirred at room temperature for 1h and then concentrated in vacuo to give a white solid. High degree of purity for **8** as a white solid was obtained using preparative-reverse phase HPLC (100 mg, 45%). M.p.188-190 °C. ^1^H NMR (400 MHz, CD_3_OD): 0.64 (d, *J* = 20.8 Hz, 3H, CH_3_), 0.90-1.01 (m, 9H, CH_3_+CH_2_+CH), 2.86-2.92 (m, 3H, PhCH_2_+CH_2_), 3.09-3.17 (m, 3H, PhCH_2_+CH_2_), 3.37-3.92 (m, 8H, CH_2_+PhCH_2_), 4.30-4.42 (m, 1H, CH), 4.55-4.65 (m, 1H, CH), 5.27-5.35 (m, 1H, CH), 7.01 (t, *J* = 7.2 Hz, 2H, ArH+Pyrrole-H), 7.15-7.30 (m, 8H, ArH), 7.66 (s, 1H, Py-H), 8.57 (s, 1H, Py-H). ^13^C NMR (100 MHz, CD_3_OD): 176.91 (C=O), 176.97 (C=O of isomer), 174.1 (C=O), 174.3 (C=O of isomer), 172.36 (C=O), 172.33 (C=O of isomer), 171.3 (C=O), 163.4 (d, *J* = 242.8 Hz, ArC), 161.88 (C=O), 161.95 (C=O of isomer), 140.9 (ArC), 138.43 (ArC), 138.49 (ArC of isomer), 137.94 (ArC), 137.92 (ArC of isomer), 136.8 (ArC), 135.93 (ArC), 135.88 (ArC of isomer), 134.1 (ArC), 132.5 (d, *J* = 7.5 Hz, ArC), 132.4 (d, *J* = 6.8 Hz, ArC of isomer), 130.3 (ArC), 130.2 (ArC of isomer), 129.4 (ArC), 129.5 (ArC of isomer), 127.83 (ArC), 127.79 (ArC of isomer), 116.7 (ArC), 116.4 (d, *J* = 4.5 Hz, ArC), 116.1 (d, *J* = 4.2 Hz, ArC), 103.1 (ArC), 74.0, 72.9, 71.40, 71.37 (from isomer) and 69.1 (CH-OH of the cholic acid residues and CH-O of piperidine), 56.0 (CH), 56.1 (CH of isomer), 53.7 (CH), 53.6 (CH of isomer), 52.0 (CH), 48.0, 47.4, 47.5 (from isomer), 43.9, 43.2 and 43.00, 43.04 (from isomer) (C , CH and CH_2_ of the cholic acid residues), 41.0 (CH_2_), 40.6, 40.5 and 38.7 (CH and CH_2_ of the cholic acid residues), 38.4 (CH_2_), 36.9 (PhCH_2_), 36.5, 35.9, 33.9, 33.33, 33.29 (from isomer), 31.8, 31.7 (from isomer) and 31.2 (C , CH and CH_2_ of the cholic acid residues), 30.7 (PhCH_2_), 29.6 (CH_2_), 28.7 (CH_2_), 27.9, 24.2 and 23.78, 23.82 (from isomer) (CH and CH_2_ of the cholic acid residues), 23.2, 17.7 and 13.02, 12.99 (from isomer) (CH_3_ of the cholic acid residues). MS (ESI) *m/z*: 1110.0 [*M*+H]^+^. HR-MS (ESI, M+H) m/z: calcd for C_61_H_82_ClFN_7_O_9_ 1110.5847, found 1110.5841.

**(*E*)-3-(2-{[4-(tert-butoxycarbonyl)phenyl] diazenyl}phenyl)propanoic acid (46).** To a solution of o-nitrocinnamic acid (**43**, 10.0 g, 52.0 mmol) in H_2_O (500 mL), NaOH (4.0 g, 0.10 mol) and 10% Pd/C (0.4 g) were added under a nitrogen atmosphere. The reaction mixture stirred at room temperature under H_2_ at atmospheric pressure for 18h and then filtered through Celite. The insoluble substance was washed with H_2_O (100 mL×3) and the filtrate was concentrated in vacuo to give **44** as a white solid (80%), which was used in the next step without further purification. **44** was dissolved in CH_2_Cl_2_/H_2_O (3:2, 500 mL), to which oxone (55.3 g, 90.0 mmol) was slowly added. After being vigorously stirred at 0 °C for 2h, the reaction mixture was diluted with water (200 mL) and extracted with CH_2_Cl_2_ (200 mL×3). The combined organic layers were washed with brine (200 mL), dried over Na_2_SO_4_, filtered, and concentrated in vacuo to afford **45** as a crude orange solid (7.5 g, 92.5%), which was used to the next step reaction without further purification. Then the crude orange solid **45** (7.5 g, 42.0 mmol) was dissolved in AcOH (75.0 mL), tert-Butyl 4-aminobenzoate (8.1 g, 42.0 mmol) was slowly added. The mixture was heated to 80^o^C and stirred for 16 hours and then concentrated in vacuo to afford the crude orange solid. The crude product was purified by column chromatography over silica gel [petroleum ether-EtOAc (10:1)] to give **46** as an orange solid (3.5 g, 17.4% for 3 steps). m.p. 119-121 °C。^1^H NMR (400 MHz, CDCl_3_): 1.63 (s, 9H, CH_3_), 2.79 (t, *J* = 8.0 Hz, 2H, CH_2_), 3.49 (t, *J* = 7.6 Hz, 2H, CH_2_), 7.32-7.36 (m, 1H, ArH), 7.41-7.47 (m, 2H, ArH), 7.72 (d, *J* = 7.6 Hz, 1H, ArH), 7.93 (dt, *J* = 8.4, 2.0 Hz, 2H, ArH), 8.15 (dt, *J* = 8.4, 2.0 Hz, 2H, ArH). ^13^C NMR (100 MHz, CDCl_3_): 179.1 (C=O), 165.2 (C=O), 155.0 (ArC), 150.1 (ArC), 140.7 (ArC), 133.8 (ArC), 132.0 (ArC), 130.7 (ArC), 130.5 (ArC), 127.5 (ArC), 122.7 (ArC), 115.6 (ArC), 81.5 (C), 36.1 (CH_2_), 28.2 (CH_2_), 26.9 (CH_3_). MS (ESI) *m/z*: 355.2 [*M*+H]^+^.

**(*E*)-1-[(S)-2-(5-Chloro-1H-pyrrolo[2,3-c]pyridine-2-carboxamido)-2-(4-fluorobenzyl)-1-oxoethyl]-piperidine-4-yl 3-(2-{[4-(tert-butoxycarbonyl)phenyl] diazenyl}phenyl)propionate (47).** Following the procedure for preparation of **22**, the esterification reaction between **46** and **PSN-357** was carried out to give **47** as an orange solid (82.9%). m.p. 123-125 °C. ^1^H NMR (400 MHz, CD_3_OD): 1.13-1.40 (m, 2H, CH_2_), 1.44-1.51 (m, 1H, CH_2_), 1.61 (d, *J* = 8.4 Hz, 9H, CH_3_), 1.68-1.78 (m, 1H, CH_2_), 2.67-2.74 (m, 2H, CH_2_), 3.01-3.15 (m, 2H, CH_2_), 3.17-3.25 (m, 1H, PhCH_2_), 3.39-3.52 (m, 4H, CH_2_), 3.56-3.68 (m, 1H, PhCH_2_), 4.79-4.83 (m, 1H, CH), 5.24 (t, *J* = 7.6 Hz, 1H, CH), 6.94-7.02 (m, 2H, ArH+Pyrrole-H), 7.13 (s, 1H, ArH), 7.24-7.48 (m, 5H, ArH), 7.62-7.72 (m, 2H, ArH), 7.93 (dd, *J* = 19.6, 8.4 Hz, 2H, ArH), 8.11 (dd, *J* = 14.8, 8.0 Hz, 2H, ArH+Py-H), 8.54 (d, *J* = 4.0 Hz, 1H, Py-H). ^13^C NMR (100 MHz, CD_3_OD): 173.65 (C=O), 173.62 (C=O of isomer), 171.3 (C=O), 171.2 (C=O of isomer), 166.6 (C=O), 163.4 (d, *J* = 246.0 Hz, ArC), 161.86 (C=O), 161.89 (C=O of isomer), 156.63 (ArC), 156.57 (ArC of isomer), 151.45 (ArC), 151.42 (ArC of isomer), 142.4 (ArC), 142.3 (ArC of isomer), 140.9 (ArC), 137.9 (ArC), 136.7 (ArC), 135.8 (ArC), 135.09 (ArC), 135.01 (ArC of isomer), 134.1 (ArC), 133.3 (ArC), 133.2 (ArC of isomer), 132.4 (ArC), 132.3 (ArC of isomer), 131.9 (ArC), 131.53 (ArC), 131.50 (ArC of isomer), 128.53 (ArC), 128.49 (ArC of isomer), 123.8 (ArC), 123.7 (ArC of isomer), 116.7 (ArC), 116.4 (ArC), 116.3 (d, *J* = 7.2 Hz, ArC), 116.1 (d, *J* = 5.5 Hz, ArC), 103.0 (ArC), 82.81 (C), 82.78 (C of isomer), 70.2 (CH), 70.4 (CH of isomer), 51.9 (CH), 43.9 (CH_2_), 43.8 (CH_2_ of isomer), 40.5 (CH_2_), 40.3 (CH_2_ of isomer), 38.3 (CH_2_), 37.6 (CH_2_), 31.7 (CH_2_), 31.8 (CH_2_ of isomer), 31.2 (PhCH_2_), 31.1(PhCH_2_ of isomer),28.4 (CH_3_), 28.2 (CH_2_). MS (ESI) *m/z*: 781.2 [*M*+H]^+^.

**(E)-1-[(S)-2-(5-Chloro-1H-pyrrolo[2,3-c]pyridine-2-carboxamido)-2-(4-fluorobenzyl)-1-oxoethyl]-piperidine-4-yl 3-(2-{[4-(carboxy)phenyl] diazenyl}phenyl)propionate (48).** Following the procedure for preparation of **34**, deprotection of **47** with TFA afforded **48** as a white solid (79.5%). M.p. 127-129 °C. ^1^H NMR (400 MHz, CD_3_OD): 1.06-1.42 (m, 2H, CH_2_), 1.44-1.77 (m, 2H, CH_2_), 2.67-2.74 (m, 2H, CH_2_), 3.01-3.17 (m, 2H, CH_2_), 3.20-3.25 (m, 1H, PhCH_2_), 3.40-3.50 (m, 4H, CH_2_), 3.56-3.69 (m, 1H, CH_2_), 4.76-4.81 (m, 1H, CH), 5.25 (t, *J* = 7.2 Hz, 1H, CH), 6.94-7.02 (m, 2H, ArH+Pyrrole-H), 7.12 (d, *J* = 2.0 Hz, 1H, ArH), 7.24-7.48 (m, 5H, ArH), 7.62-7.72 (m, 2H, ArH), 7.93 (dd, *J* = 20.4, 8.0 Hz, 2H, ArH), 8.18 (dd, *J* = 14.0, 4.8 Hz, 2H, ArH+Py-H), 8.54 (d, *J* = 2.8 Hz, 1H, Py-H). ^13^C NMR (100 MHz, CD_3_OD): 173.65 (C=O), 173.64 (C=O of isomer), 171.3 (C=O), 171.2 (C=O of isomer), 169.3（C=O）, 163.4 (d, *J* = 241.9 Hz, ArC), 161.89 (C=O), 161.87 (C=O of isomer), 156.7 (ArC), 156.6 (C=O of isomer), 151.45 (ArC), 151.41 (ArC of isomer), 142.35 (ArC), 142.26 (ArC of isomer), 140.9 (ArC), 137.9 (ArC), 136.8 (ArC), 135.8 (ArC), 134.1 (ArC), 133.24 (ArC), 133.18 (ArC of isomer), 132.4 (ArC), 132.3 (ArC of isomer), 131.91 (ArC), 131.89 (ArC of isomer), 128.52 (ArC), 128.49 (ArC of isomer), 123.8 (ArC), 123.7 (ArC of isomer), 116.7 (ArC), 116.4 (ArC), 116.3 (d, *J* = 6.0 Hz, ArC), 116.1 (d, *J* = 5.8 Hz, ArC), 103.0 (ArC), 70.2 (CH), 70.4 (CH of isomer), 52.0 (CH), 43.89 (CH_2_), 43.82 (CH_2_ of isomer), 40.5 (CH_2_), 40.3 (CH_2_ of isomer), 38.35 (CH_2_), 38.32 (CH_2_ of isomer), 37.6 (PhCH_2_), 31.8 (CH_2_), 31.7 (CH_2_ of isomer), 31.2 (PhCH_2_), 31.1 (PhCH_2_ of isomer), 28.2 (CH_2_). MS (ESI) *m/z*: 725.1 [*M*+H]^+^.

**N-{2-[(tert-butoxycarbonyl)amino]ethyl}-3*α*, 7*α*, 12*α*-trihydroxy-5*β*-cholan-24-amide (49).** Cholic acid (**17**, 2.0 g, 4.9 mmol) was dissolved in 5 mL of anhydrous DMF (20 mL), to which DEPC (879.0 mg, 5.4 mmol), Et_3_N (0.51 g, 5.0 mmol) were added at 0 °C. After the mixture was stirred for 45 min at 0 °C, N-boc-ethylenediamine (784 mg, 4.9 mmol) was added. The mixture was stirred at room temperature over night and then filtered. The filtrate was concentrated in vacuo. The crude product was purified by column chromatography over silica gel [EtOAc-THF (10:1)] to give **49** as a white solid (2.0 g, 74%). M.p. 210-212 °C. ^1^H NMR (400 MHz, *d_6_*-DMSO): 0.54 (s, 3H, CH_3_), 0.76 (s, 3H, CH_3_), 0.87 (d, *J* = 5.6 Hz, 3H, CH_3_), 1.33 (s, 9H, CH_3_), 2.90-2.92 (m, 2H, CH_2_), 2.97-3.00 (m, 2H, CH_2_), 3.11-3.17 (m, 1H, CH), 3.57 (s, 1H, CH), 3.74 (s, 1H, OH), 3.98-4.01 (m, 1H, CH), 4.07 (s, 1H, OH), 4.30 (s, 1H, OH), 6.73 (t, *J* = 5.6 Hz, 1H, NH), 7.73 (t, *J* = 5.2 Hz, 1H, NH). ^13^C NMR (100 MHz, *d_6_*-DMSO): 172.6 (C=O), 79.2 ((CH_3_)_3_CO), 70.9, 70.4 and 66.2 (CH-OH of the cholic acid residues), 46.0, 45.7, 41.5, 41.3, 39.5, 35.3, 34.9, 34.8, 34.3, 32.0, 30.8, 30.4, 28.5, 27.7, 27.3, 26.2 and 22.7 (CH_2_, CH and C of the cholic acid residues), 22.6, 16.8 and 12.2 (CH_3_ of the cholic acid residues). MS (ESI) *m/z*: 551.2 [*M*+H]^+^.

**N-(2-aminoethyl)-3*α*, 7*α*, 12*α*-trihydroxy-5*β*-cholan-24-amide (50).** To a solution of **49** (200.0 mg, 0.36 mmol) in MeOH (20 mL) was added dropwise a solution of HCl in MeOH **(**6N, 20 mL**)** at 0 °C. The reaction mixture stirred at room temperature for 2 and then concentrated in vacuo to give a white solid, which was almost a pure product, and was used for the next reaction without further purification. Crystallization of the crude product from ethanol gave **50** as a white solid (170 mg, 97%). M.p. 145-147 °C. ^1^H NMR (400 MHz, *d_6_*-DMSO): 0.54 (s, 3H, CH_3_), 0.76 (s, 3H, CH_3_), 0.88 (d, J = 6.0 Hz, 3H, CH_3_), 2.76-2.80 (m, 2H, CH_2_), 3.12-3.18 (m, 1H, CH), 3.21-3.26 (m, 2H, CH_2_), 3.56 (s, 1H, CH), 3.74 (s, 1H, CH), 4.23 (s, 6H, OH), 8.10-8.14 (m, 3H, NH+NH_2_). ^13^C NMR (100 MHz, *d_6_*-DMSO): 173.8 (C=O), 71.4, 70.9 and 66.7 (CH-OH of the cholic acid residues), 46.5, 46.2, 42.0, 41.8, 39.0, 36.8, 35.7, 35.6, 35.3, 34.8, 32.8, 31.8, 30.8, 29.0, 27.8, 26.7 and 23.3(CH_2_, CH and C of the cholic acid residues), 23.1, 17.6 and 12.8 (CH_3_ of the cholic acid residues). MS (ESI) *m/z*: 473.3 [*M*+Na]^+^.

**(S)-N-{(*E*)-[2-(4-{[2-(3-oxo-3-{[(S)*-*2-(5-chloro-1*H*-pyrrolo[2,3-c]pyridine-2-carboxamido)-2-(4-fluorobenzyl)-1-oxoethyl]-piperidin-4-yloxy}propyl)phenyl]diazenyl}benzamido)ethyl]}-3*α*, 7*α*, 12*α*-trihydroxy-5*β*-cholan-24-amide (9).** Following the procedure for preparation of **3**, the amidation reaction of **48** with **50** was carried out to give **9** as an orange solid (25 mg, 10.4%). m.p. 172-174 °C. ^1^H NMR (400 MHz, CD_3_OD): 0.54 (d, *J* = 12.4 Hz, 3H, CH_3_), 0.83 (d, *J* = 5.6 Hz, 3H, CH_3_), 0.97 (t, *J* = 5.2 Hz, 3H, CH_3_), 2.68-2.75 (m, 2H, CH_2_), 3.03-3.18 (m, 2H, CH), 3.35-3.66 (m, 10H, CH+PhCH_2_+CH_2_), 3.68-3.71 (m, 1H, PhCH_2_), 3.86 (d, *J* = 3.5 Hz, 1H, PhCH_2_), 4.63 (s, 1H, CH_2_), 4.83-4.86 (m, 1H, CH), 5.24-5.31 (m, 1H, CH), 6.95-7.04 (m, 2H, ArH+Pyrrole-H), 7.15 (d, *J* = 6.0 Hz, 1H, ArH), 7.26-7.50 (m, 5H, ArH), 7.65-7.74 (m, 2H, ArH), 7.92-8.05 (m, 4H, ArH+Py-H), 8.56 (d, *J* = 8.0 Hz, 1H, Py-H). ^13^C NMR (100 MHz, CD_3_OD): 176.2 (C=O), 176.1(C=O of isomer), 172.2 (C=O), 169.94 (C=O), 169.89 (C=O of isomer), 167.9 (C=O), 162.0 (d, *J* = 242.8 Hz, ArC), 160.5 (C=O), 154.4 (ArC), 154.5 (ArC of isomer), 149.9 (ArC), 149.85 (ArC of isomer), 140.9 (ArC), 140.81 (ArC of isomer), 139.5 (ArC), 136.5 (ArC), 136.11 (ArC), 136.07 (ArC of isomer), 135.32 (ArC), 135.30 (ArC of isomer), 134.5 (ArC), 132.7 (ArC), 132.6 (ArC), 131.8 (ArC), 131.0 (ArC), 130.9 (ArC), 130.5 (ArC), 128.21 (ArC), 128.19 (ArC of isomer), 127.1 (ArC), 122.6 (ArC), 122.5 (ArC), 115.3 (ArC), 115.0 (ArC), 114.9 (ArC), 114.7 (d, *J* = 6.0 Hz, ArC), 101.7 (ArC), 72.6, 71.4, 69.0, 68.8 (from isomer) and 67.6 (CH-OH of the cholic acid residues and CH-O of piperidine), 50.59 (CH), 50.56 (CH of isomer), 46.74, 46.71 (from isomer) and 46.0 (C and CH of the cholic acid residues), 42.51 (CH_2_), 42.42 (CH of isomer), 41.7, 41.5, 40.0, 39.5 and 39.03, 39.07 (from isomer), 38.96 (from isomer) (CH and CH_2_ of the cholic acid residues), 38.56 (CH_2_), 38.51 (CH_2_ of isomer), 36.9 (PhCH_2_), 36.4, 36.3 (from isomer), 35.30, 35.27 (from isomer), 35.0, 34.4, 34.3, 32.8 and 31.7 (C , CH and CH_2_ of the cholic acid residues), 30.4 (CH_2_), 30.3 (CH_2_ of isomer), 29.75, 29.84 (from isomer), 29.69 (from isomer), 28.1 and 27.2 (CH and CH_2_ of the cholic acid residues), 26.9 (CH_2_), 26.8 (CH_2_ of isomer), 26.4 and 22.8 (CH_2_ of the cholic acid residues), 21.7, 16.3, 11.6 (CH_3_ of the cholic acid residues). MS (ESI) *m/z*: 1157.6 [*M*+H]^+^. HRMS (ESI) [M+H]^+^ m/z: 1157.5637 (calcd for C_64_H_79_ClFN_8_O_9_, 1157.5643).

**Copy of ^1^H NMR of 12**

**
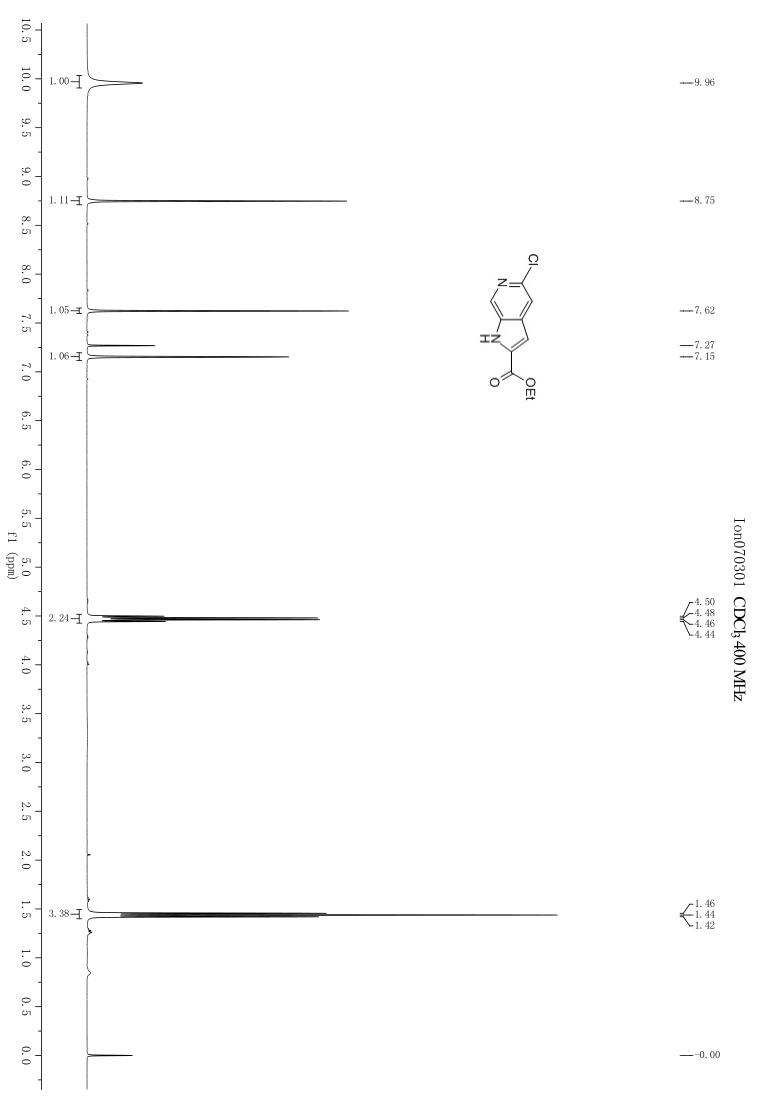
**

# Copy of ^13^C NMR of 12

#
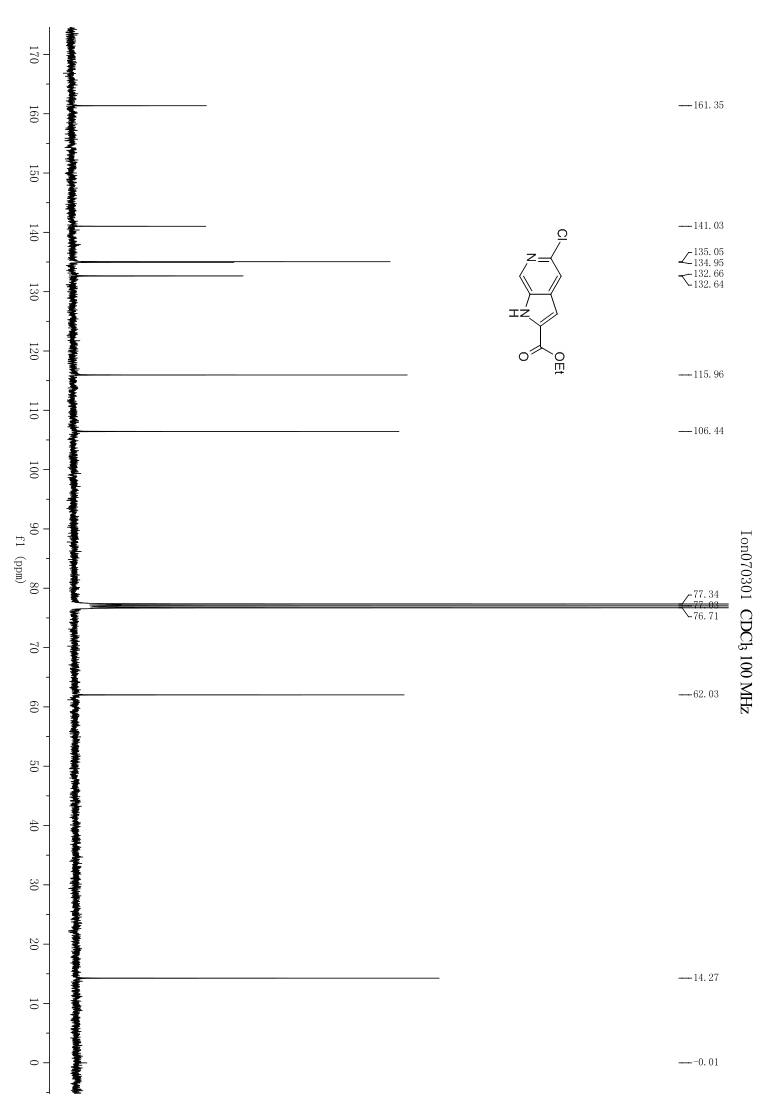


# Copy of ^1^H NMR of 13

#
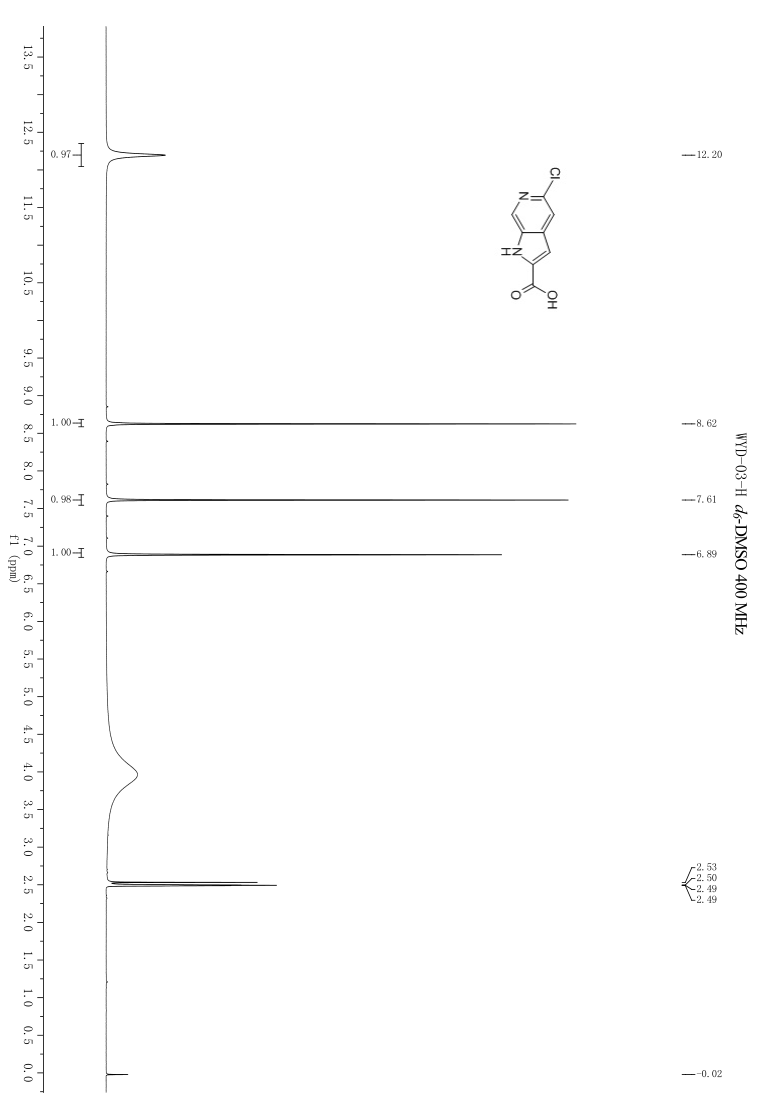


# Copy of ^13^C NMR of 13

#
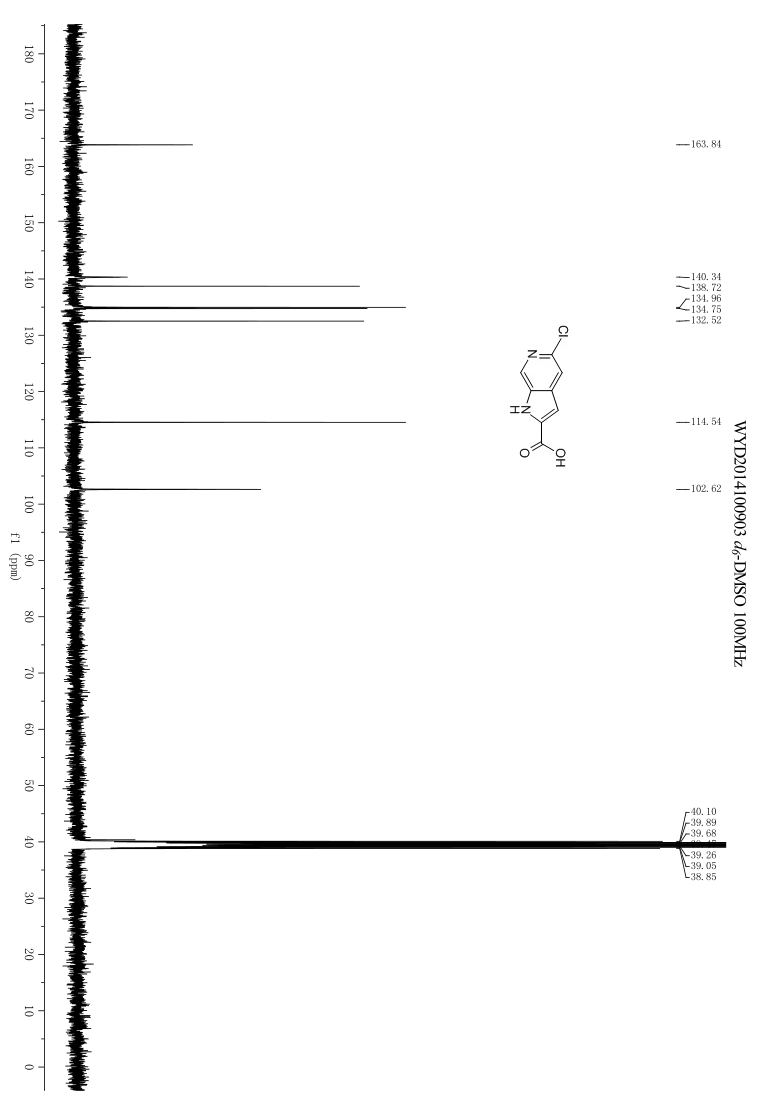


# Copy of ^1^H NMR of 15

#
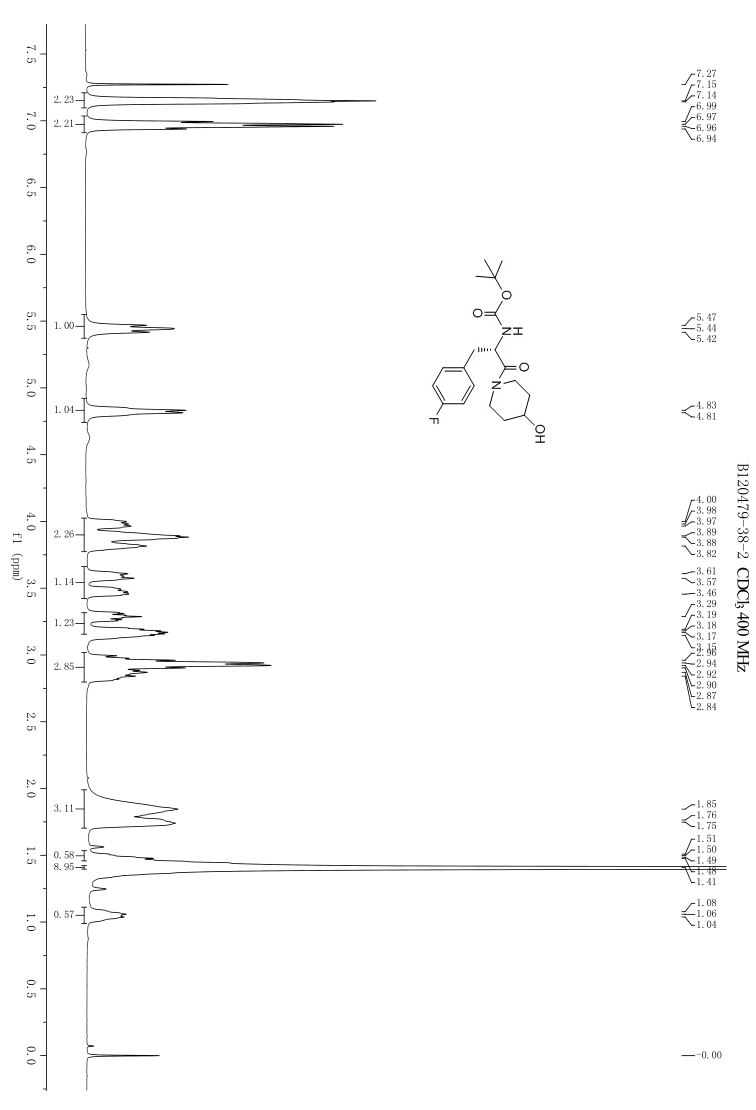


# Copy of ^13^C NMR of 15

#
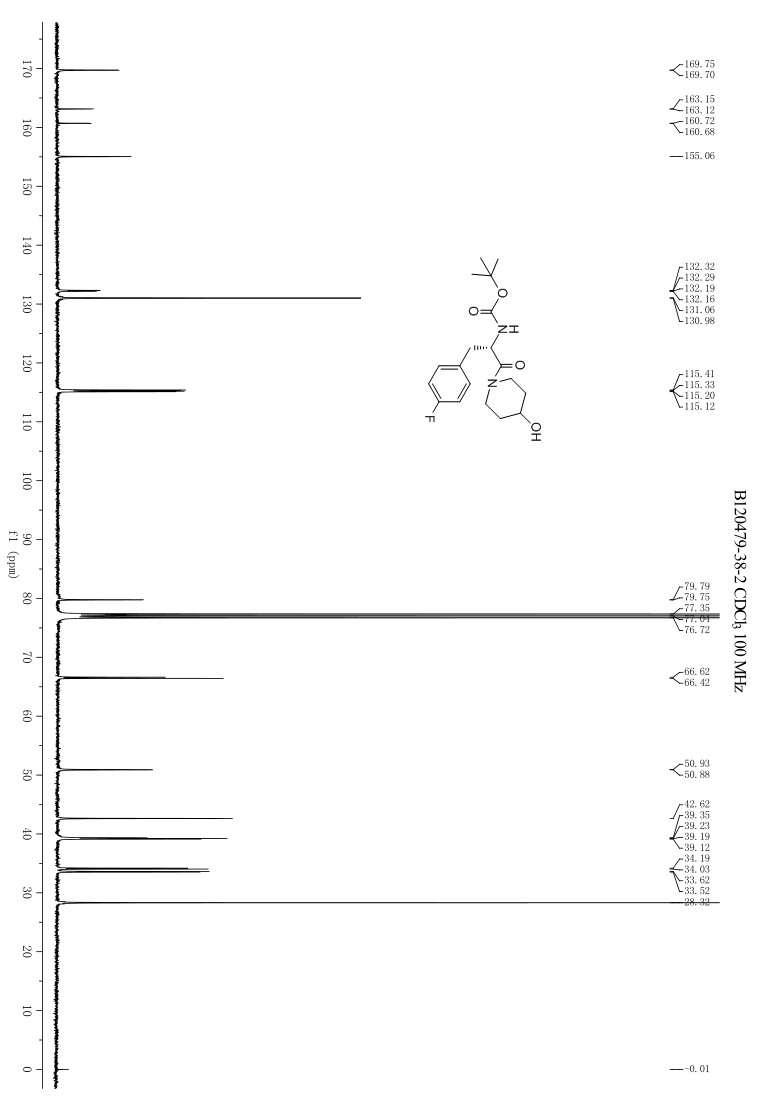


# Copy of ^1^H NMR of 16

#
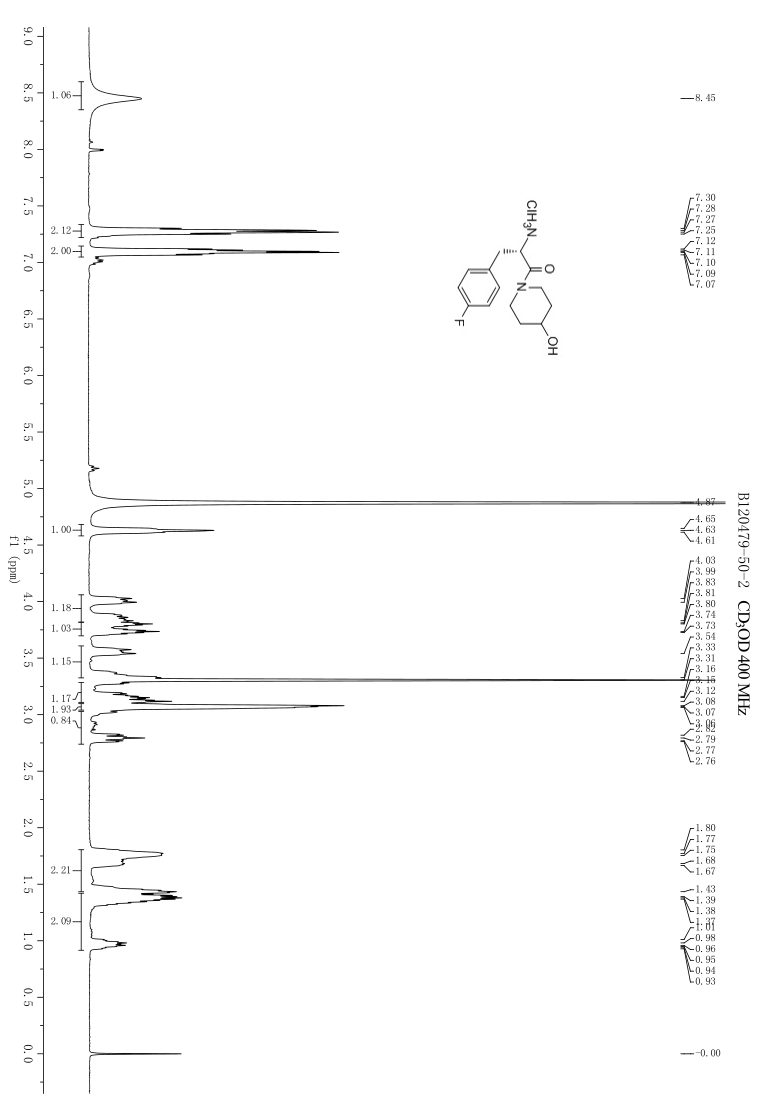


# Copy of ^13^C NMR of 16

#
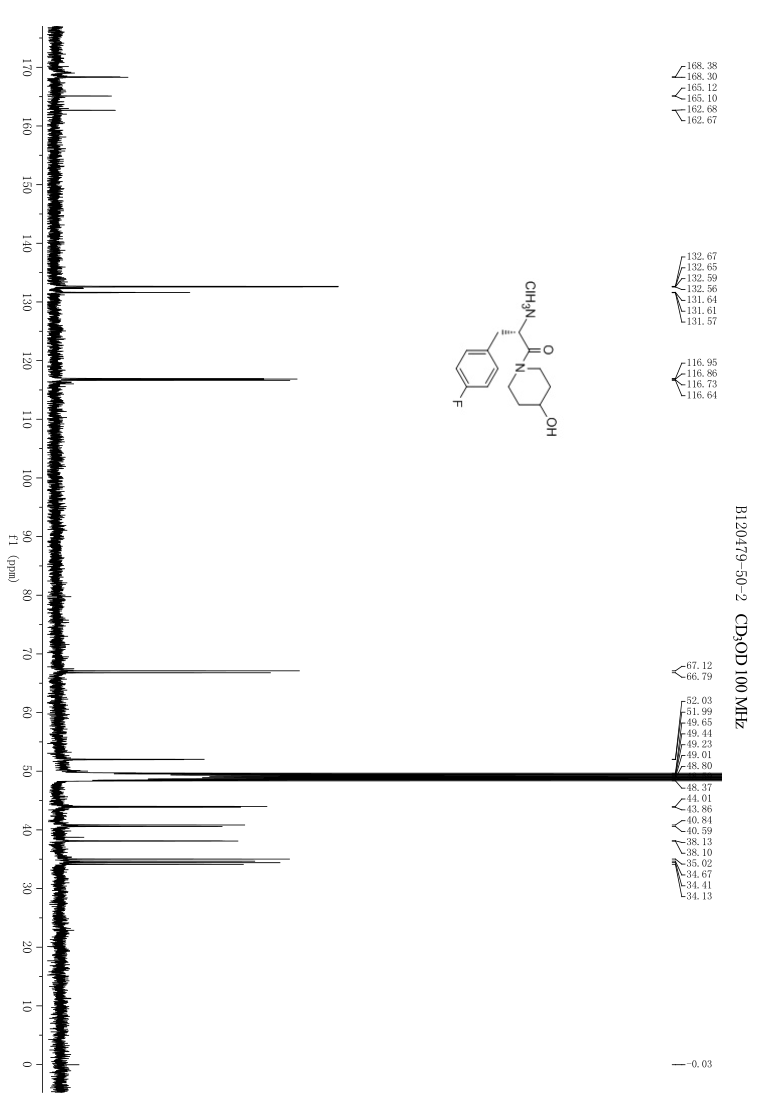


# Copy of ^1^H NMR of PSN-357


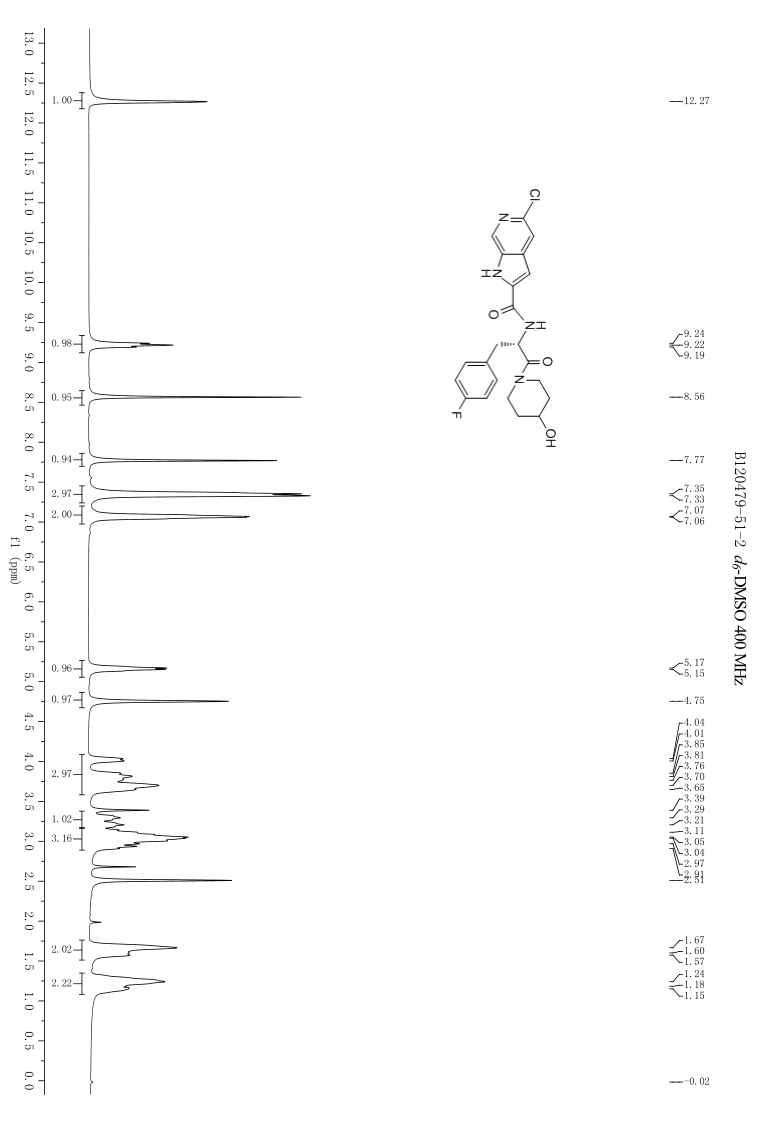


**Copy of ^13^C NMR of PSN-357**

**
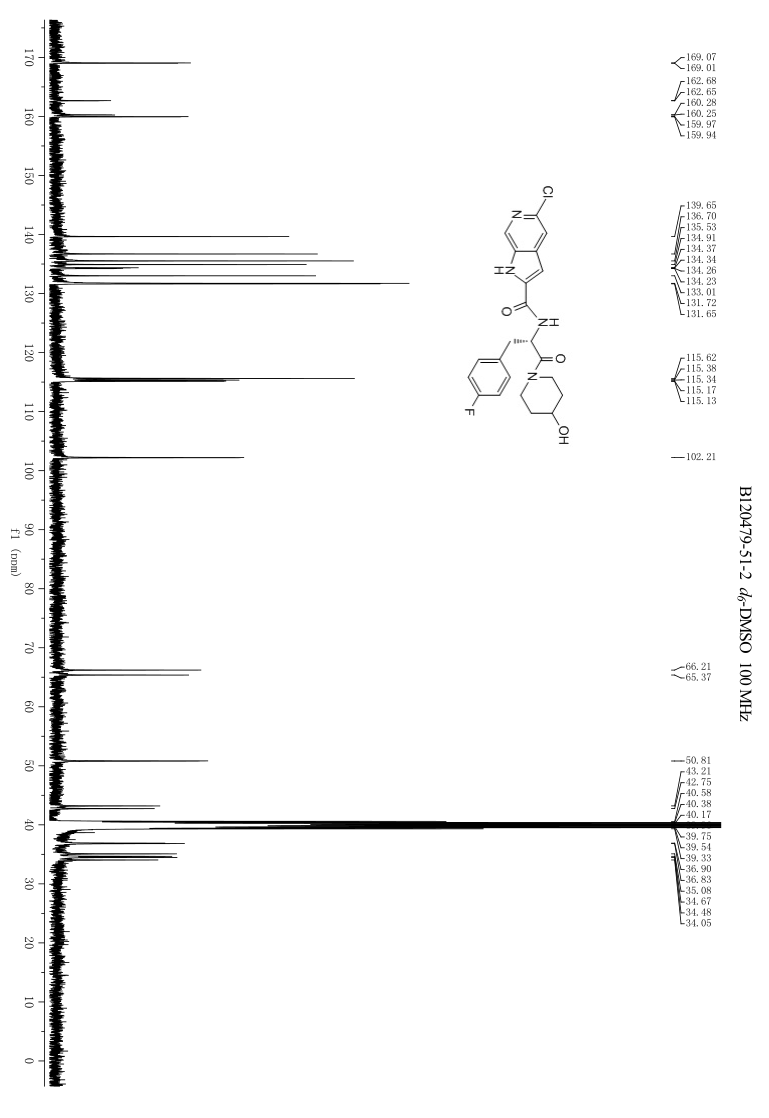
**

**Copy of ^1^H NMR of 18**

**
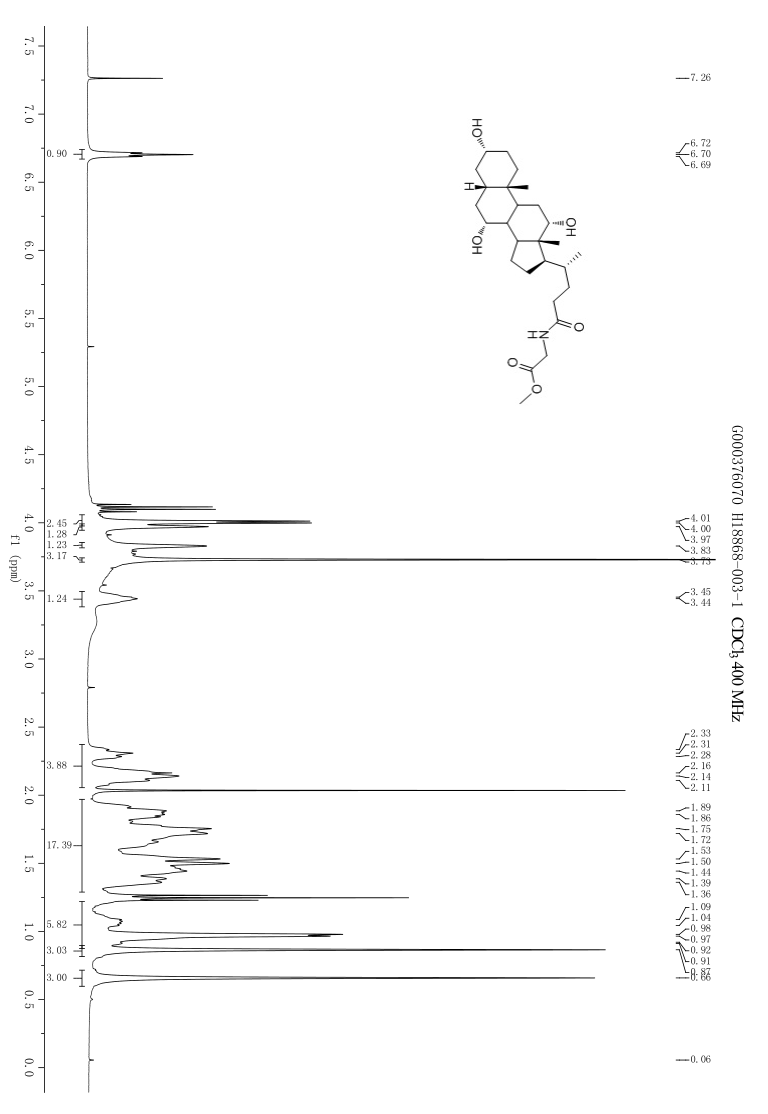
**

Copy of ^13^C NMR of 18

# **
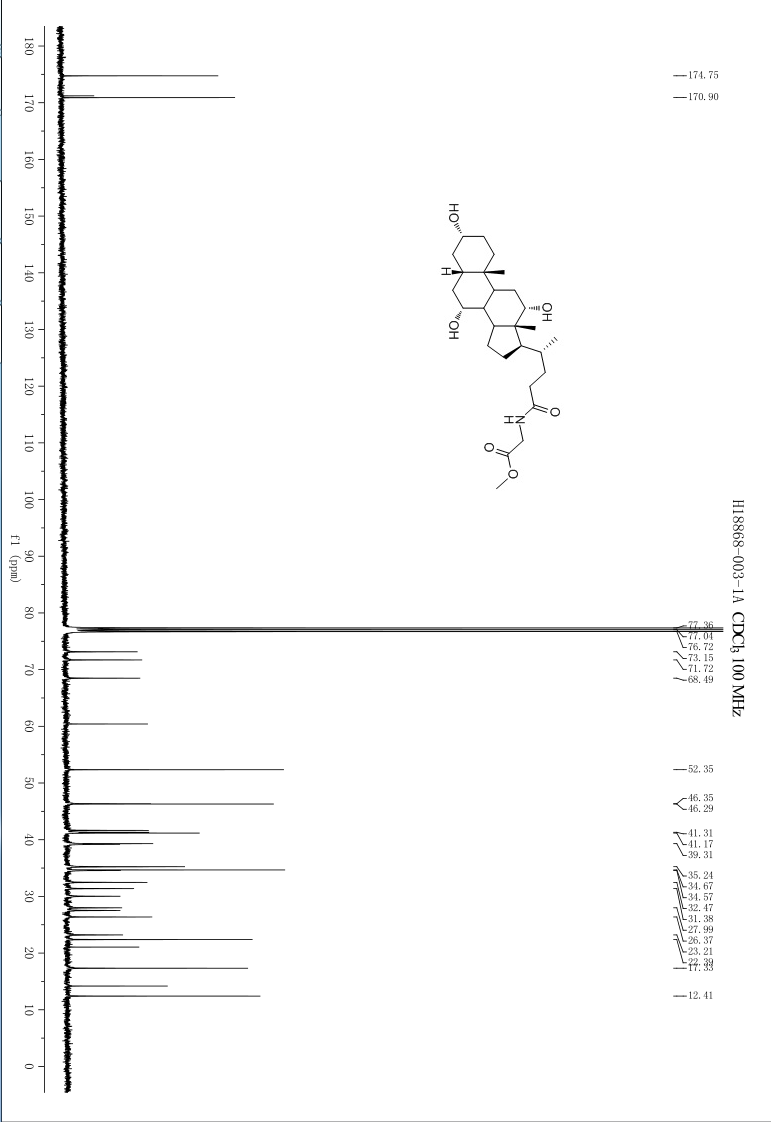
**

# Copy of ^1^H NMR of 19

#
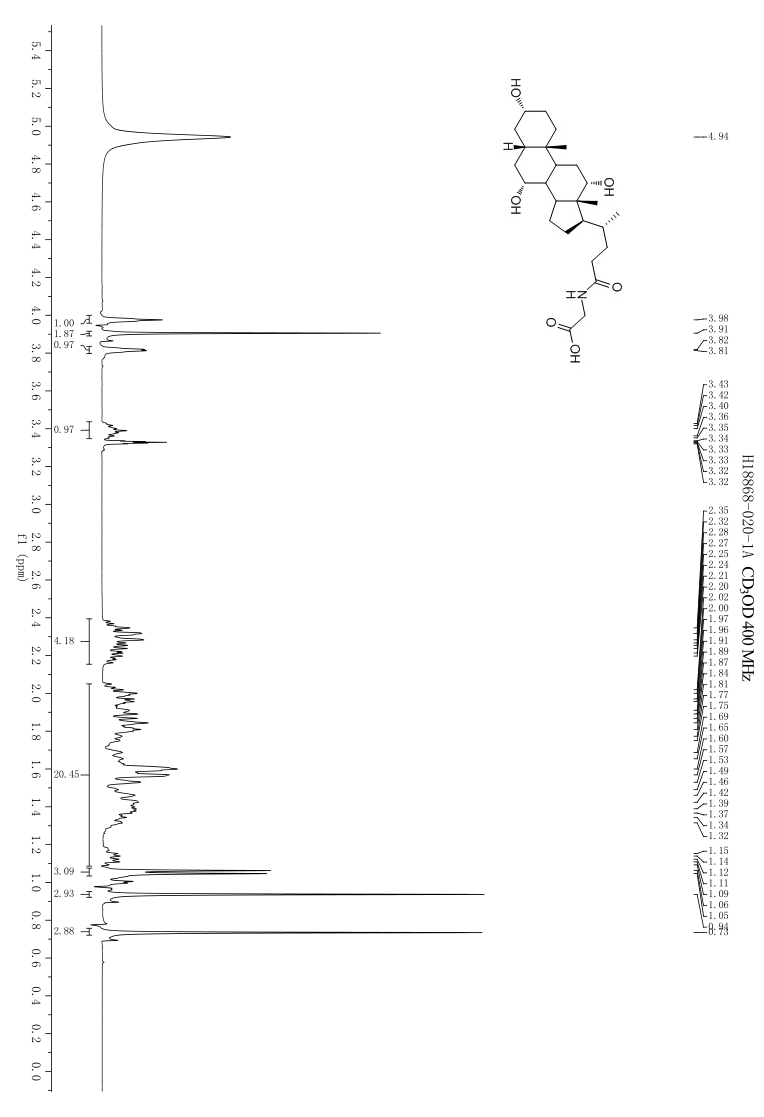


# Copy of ^13^C NMR of 19

#
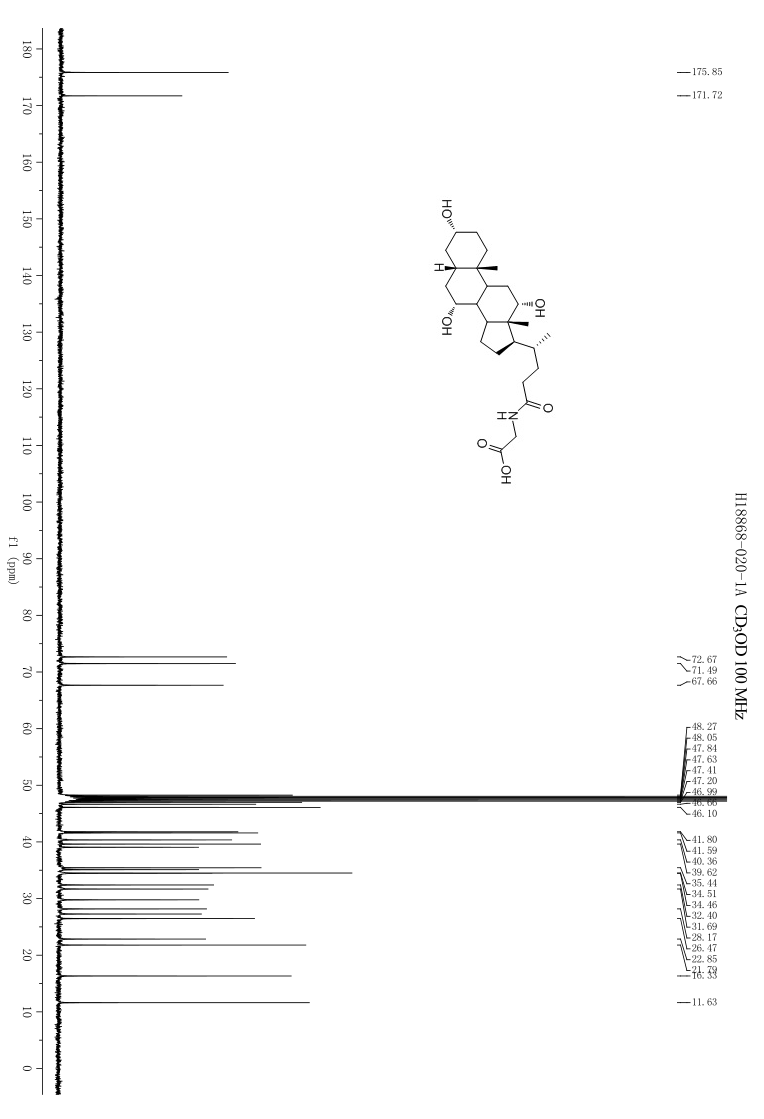


# Copy of ^1^H NMR of 1

#
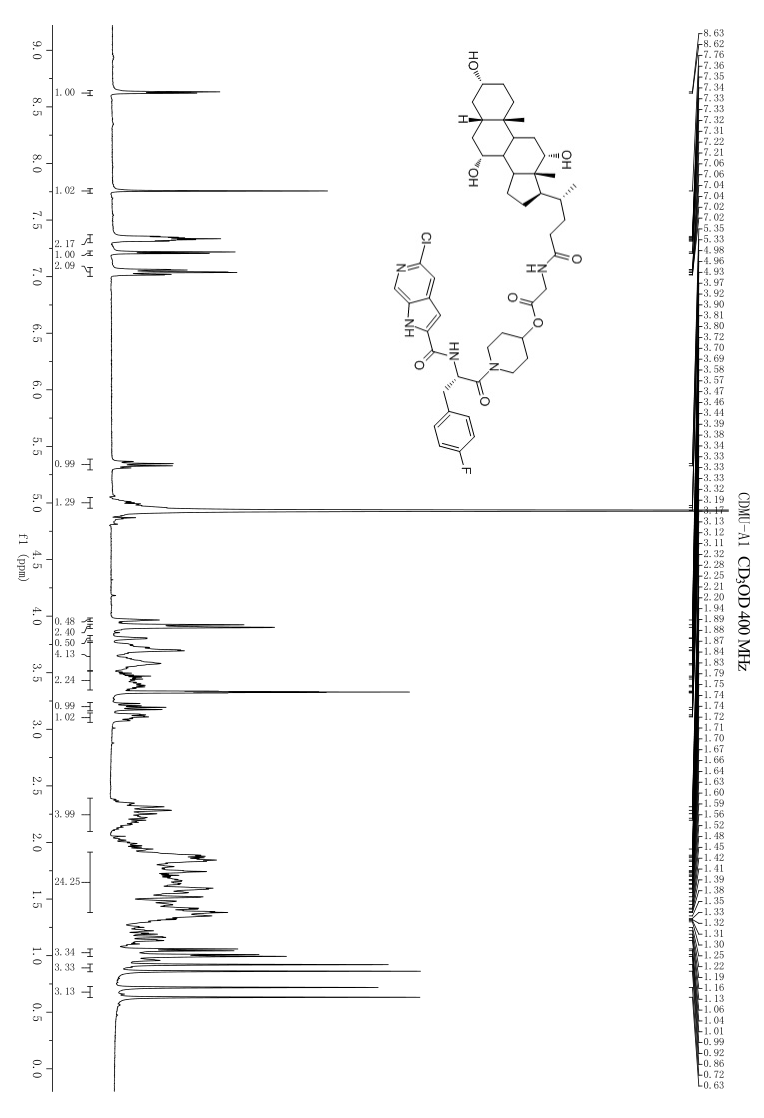


# Copy of ^13^C NMR of 1

#
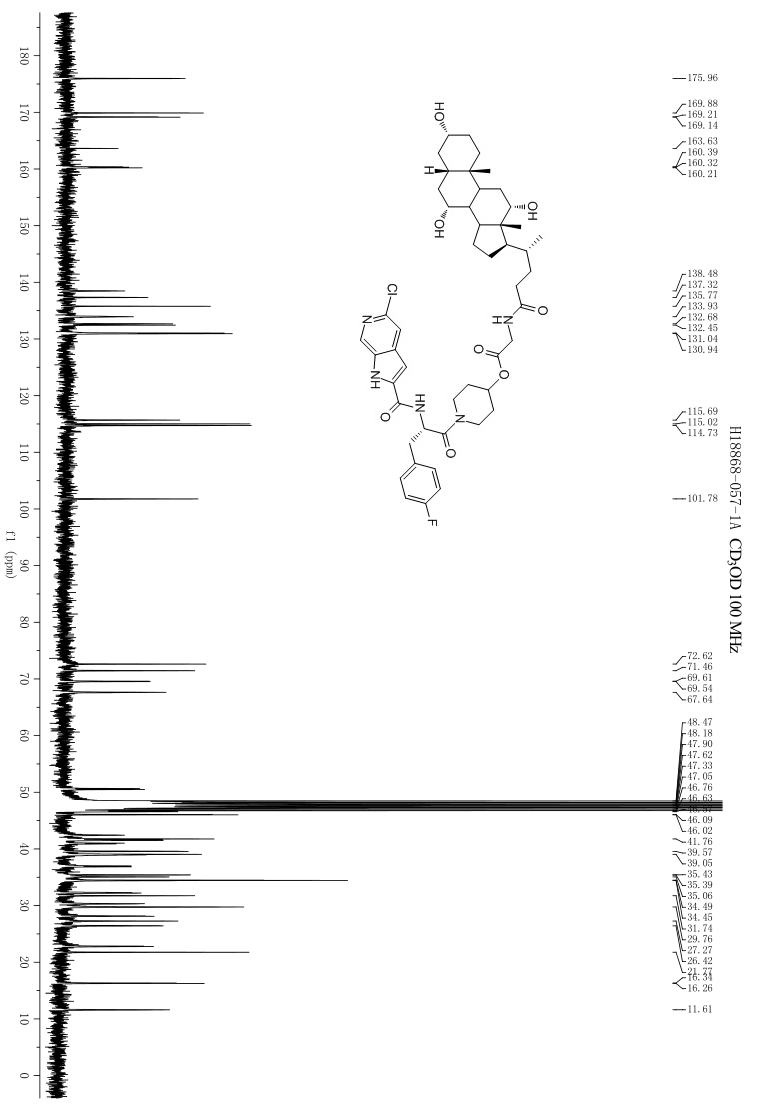


# Copy of ^1^H NMR of 20

#
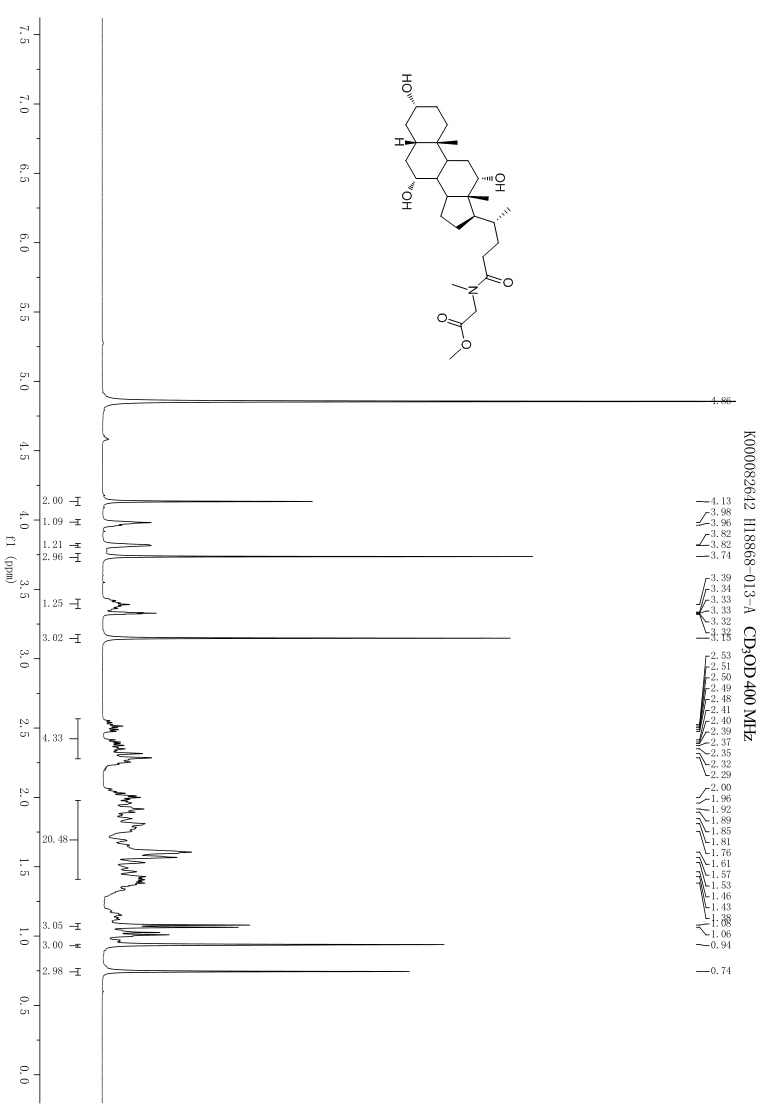


# Copy of ^13^C NMR of 20

#
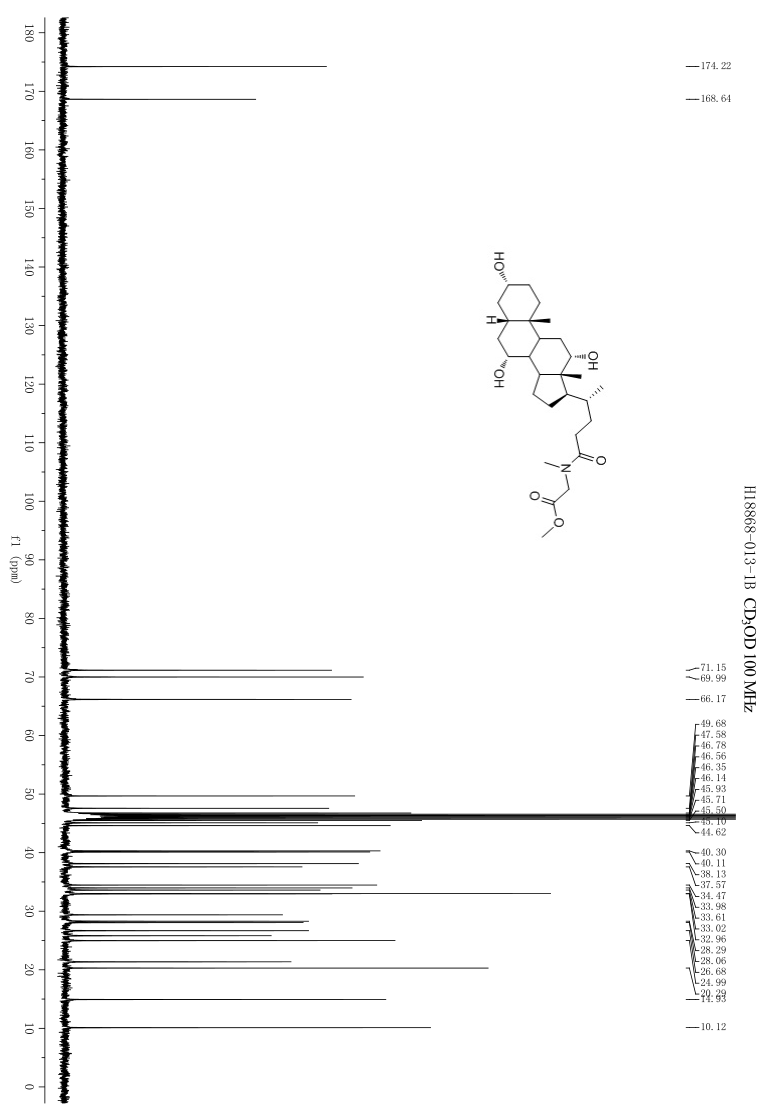


# Copy of ^1^H NMR of 21

#
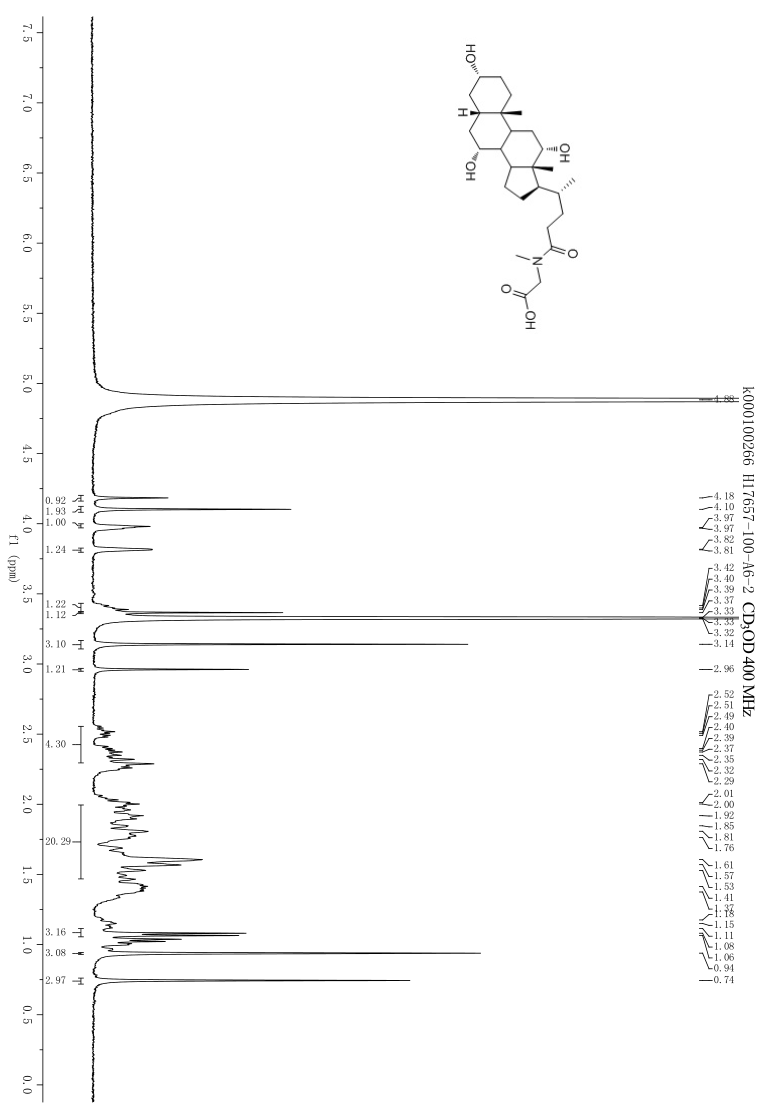


# Copy of ^13^C NMR of 21

#
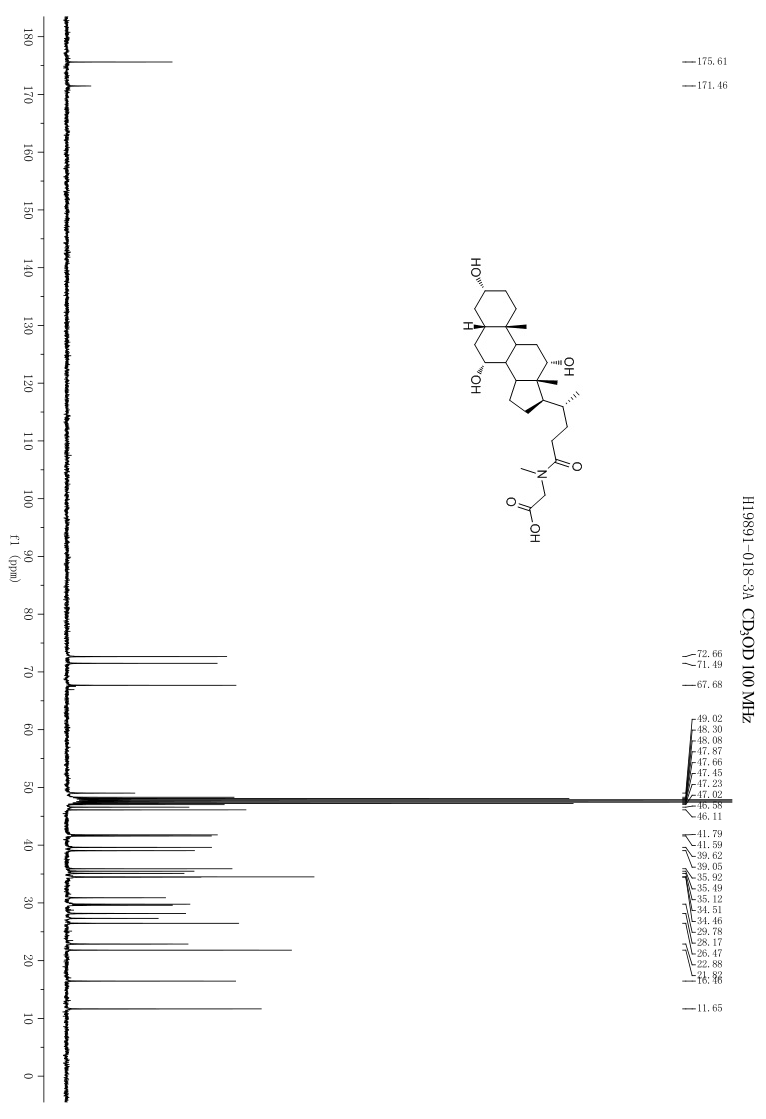


# Copy of ^1^H NMR of 2

#
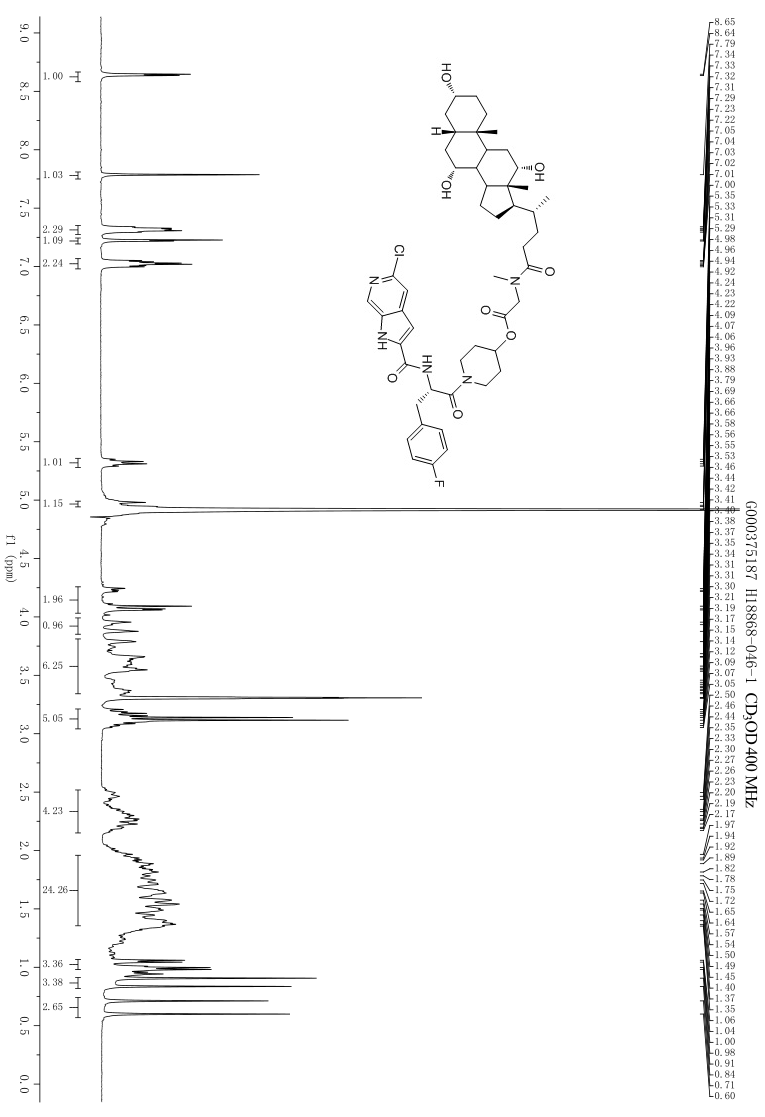


# Copy of ^13^C NMR of 2

#
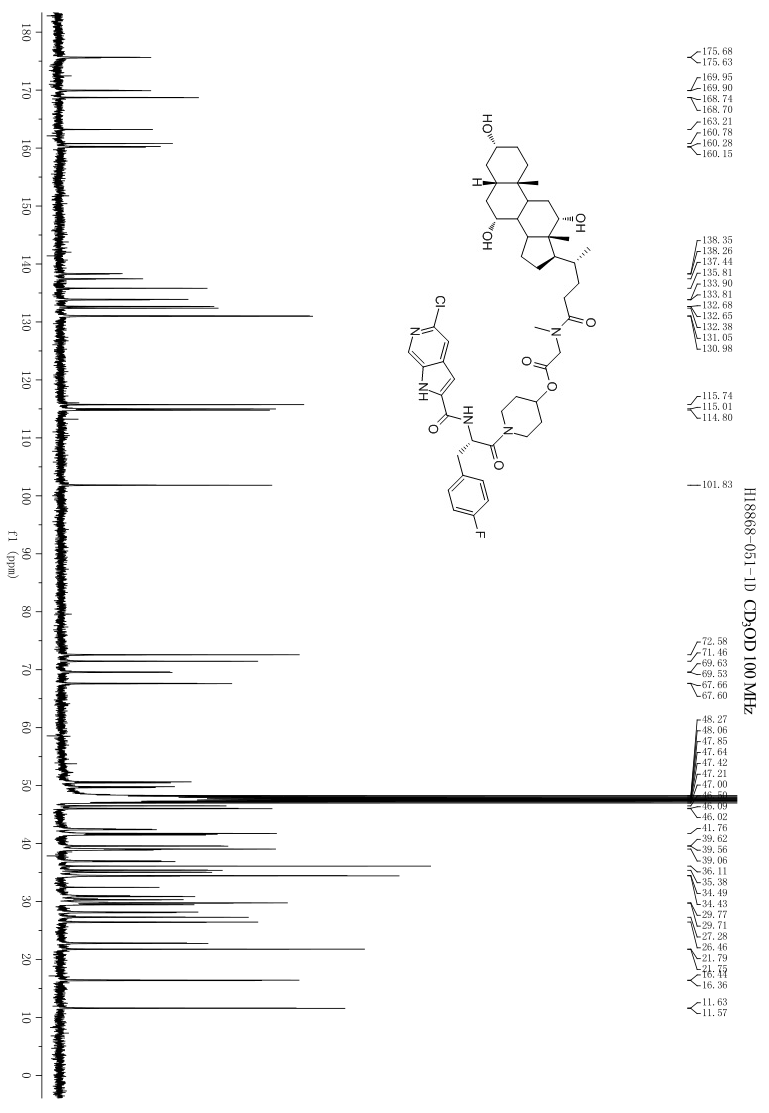


# Copy of ^1^H NMR of 23

#
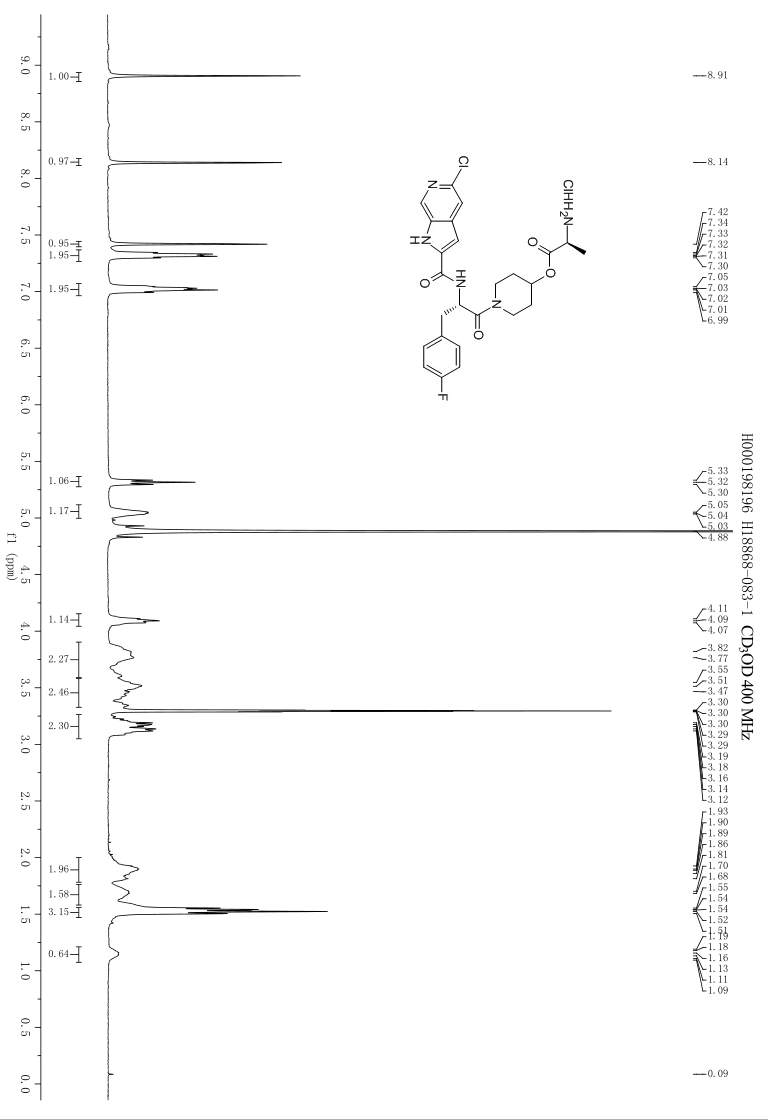


# Copy of ^13^C NMR of 23

#
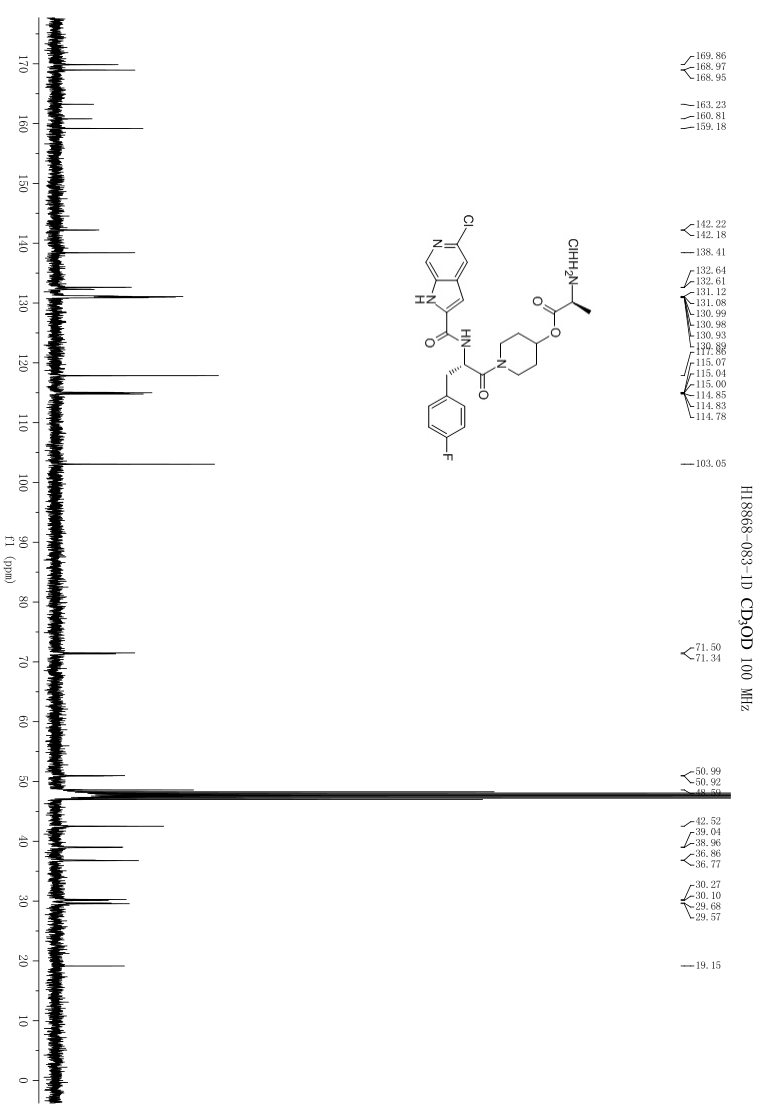


# Copy of ^1^H NMR of 3

#
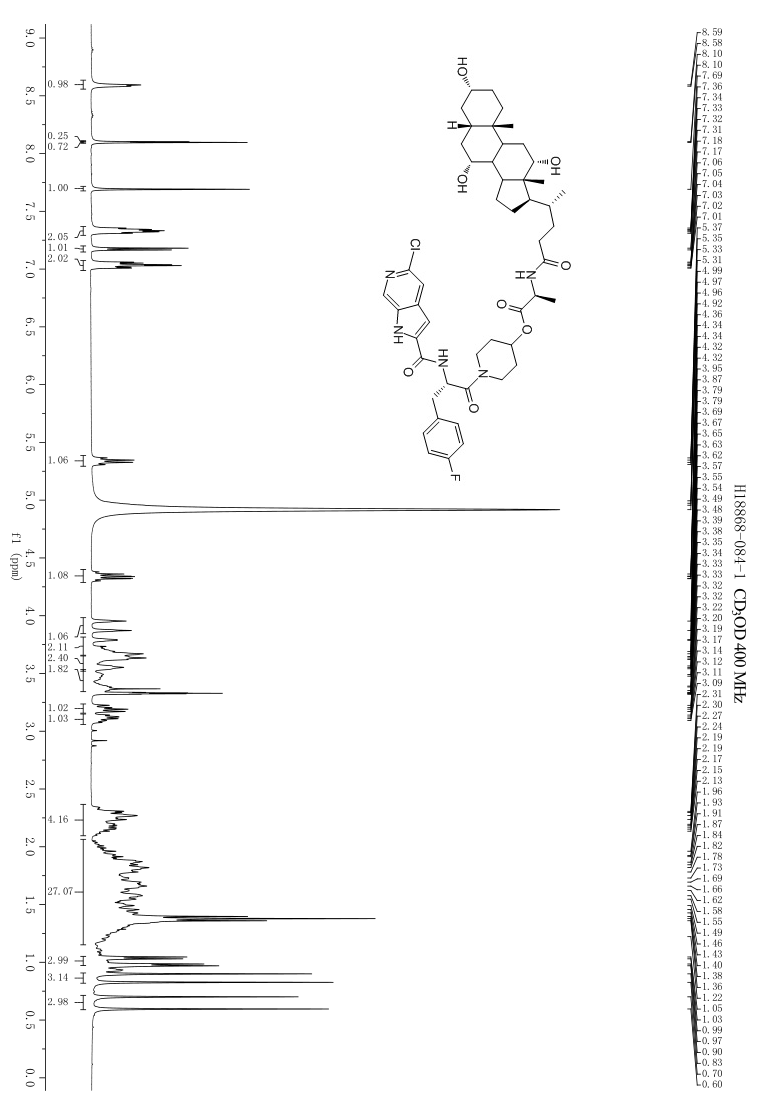


# Copy of ^13^C NMR of 3

#
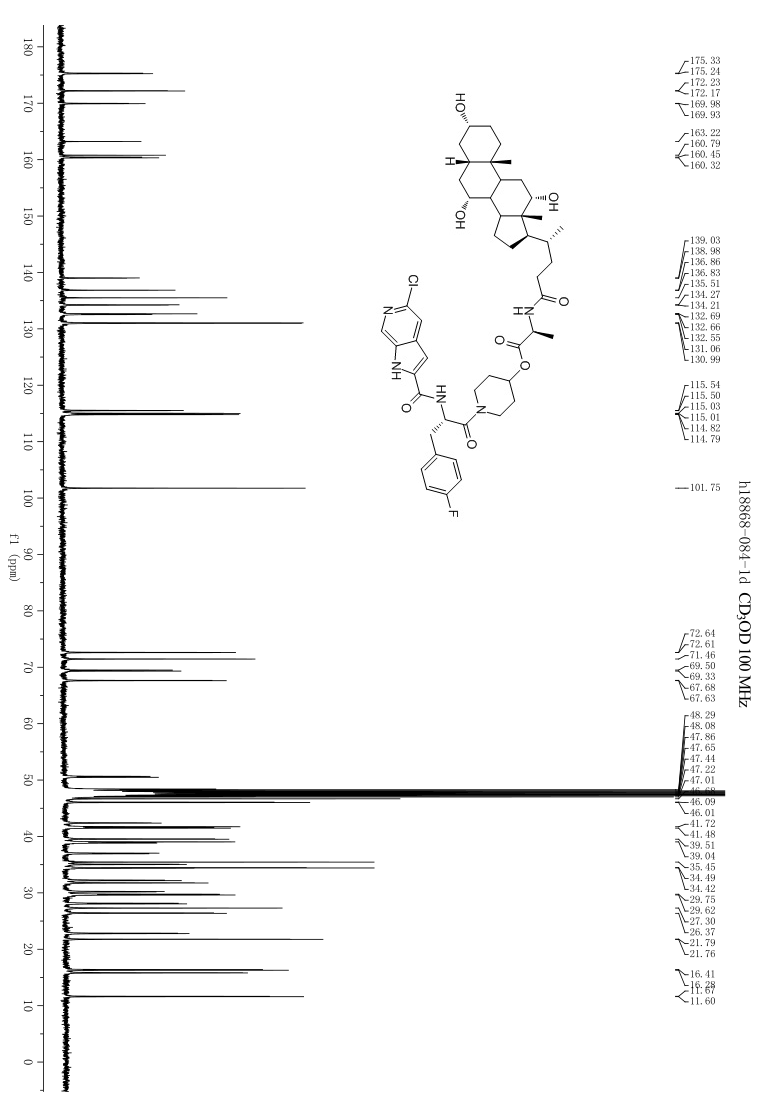


# Copy of ^1^H NMR of 25

#
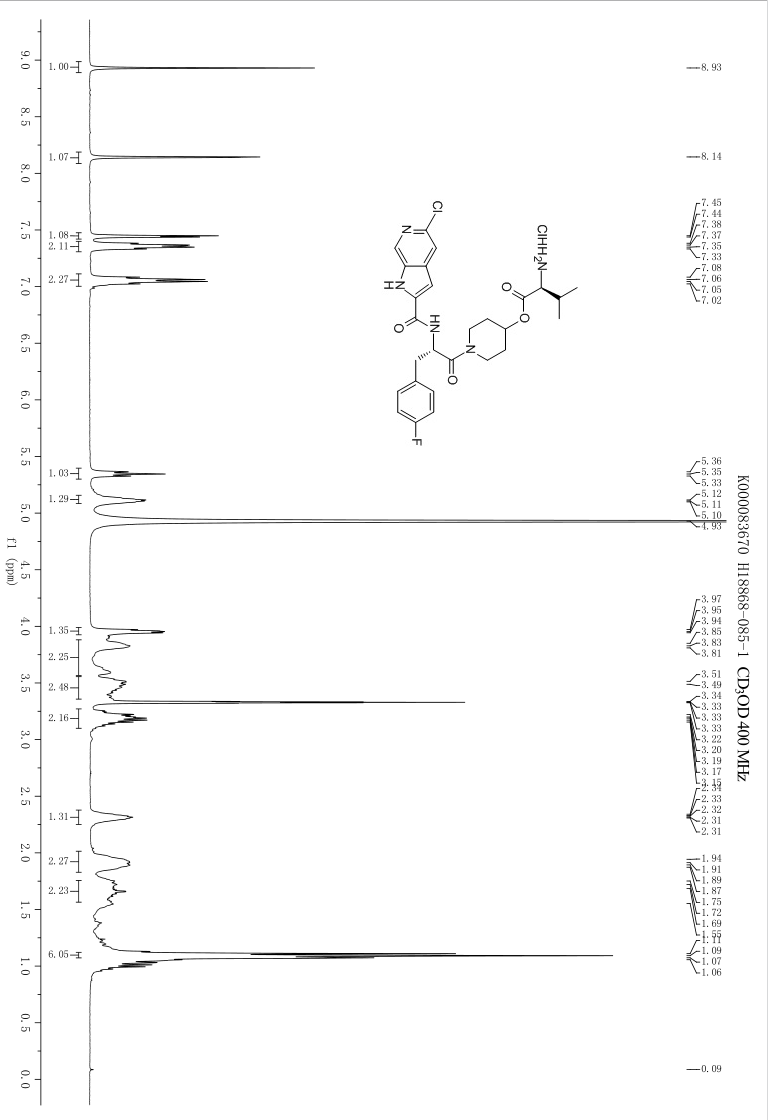


# Copy of ^13^C NMR of 25

#
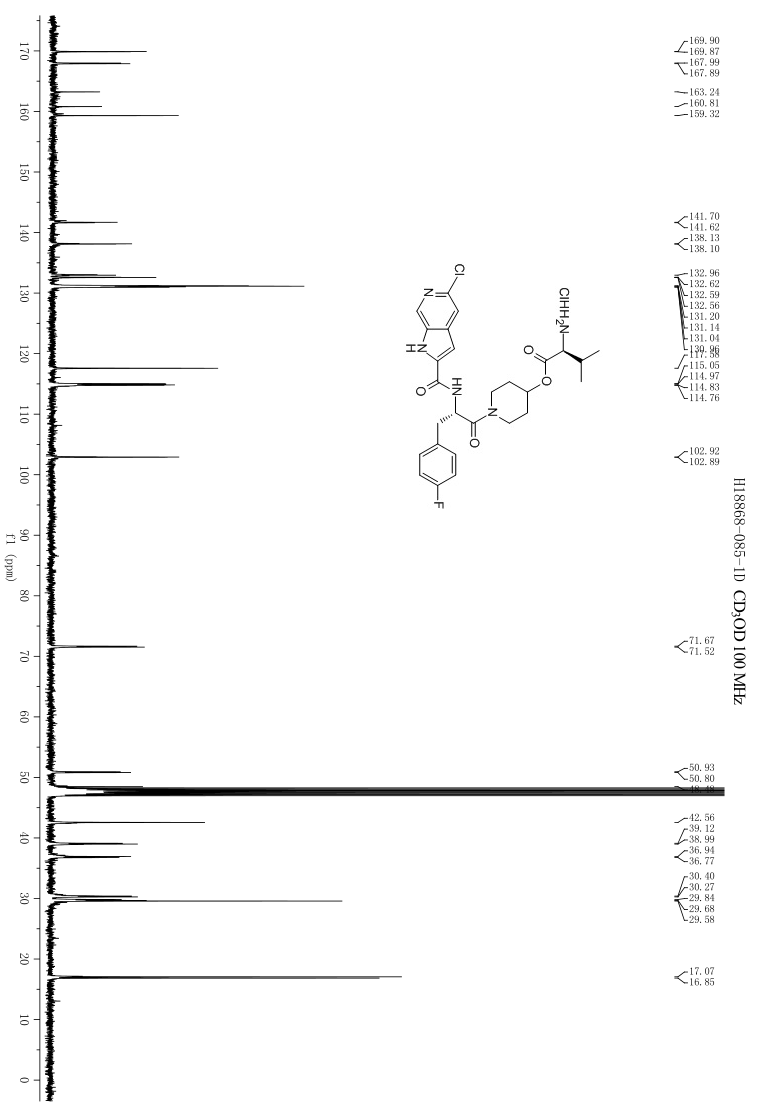


# Copy of ^1^H NMR of 4

#
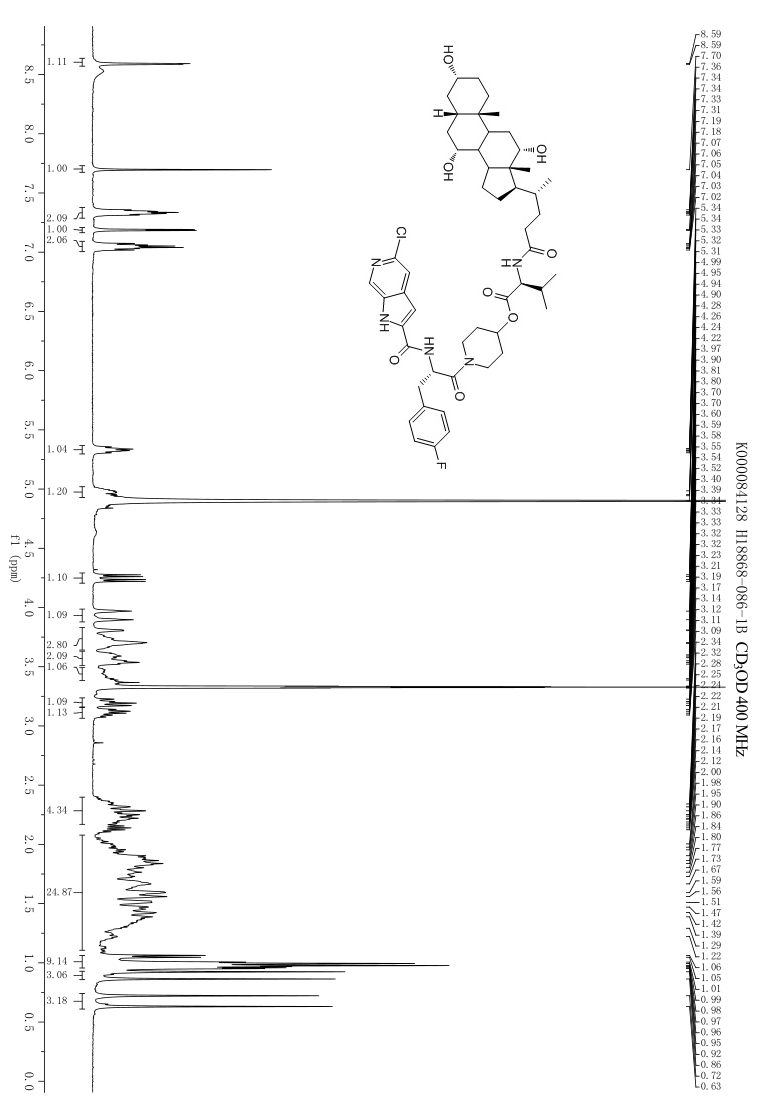


# Copy of ^13^C NMR of 4

#
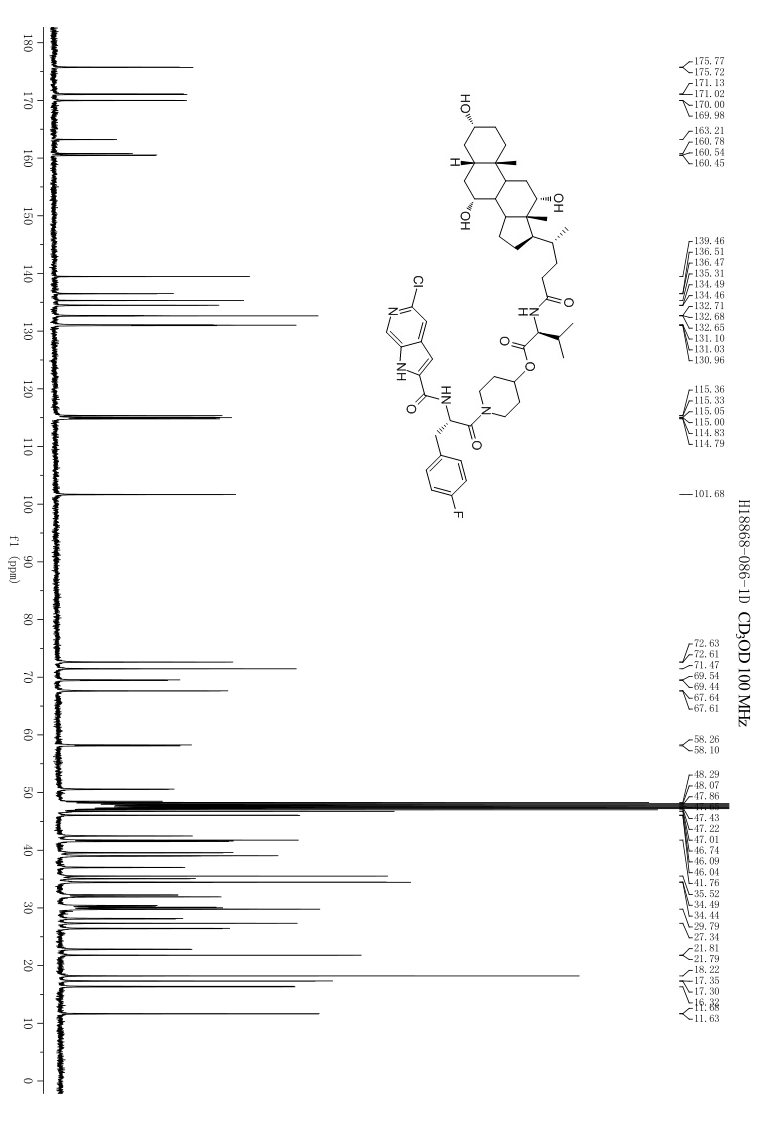


# Copy of ^1^H NMR of 27

#
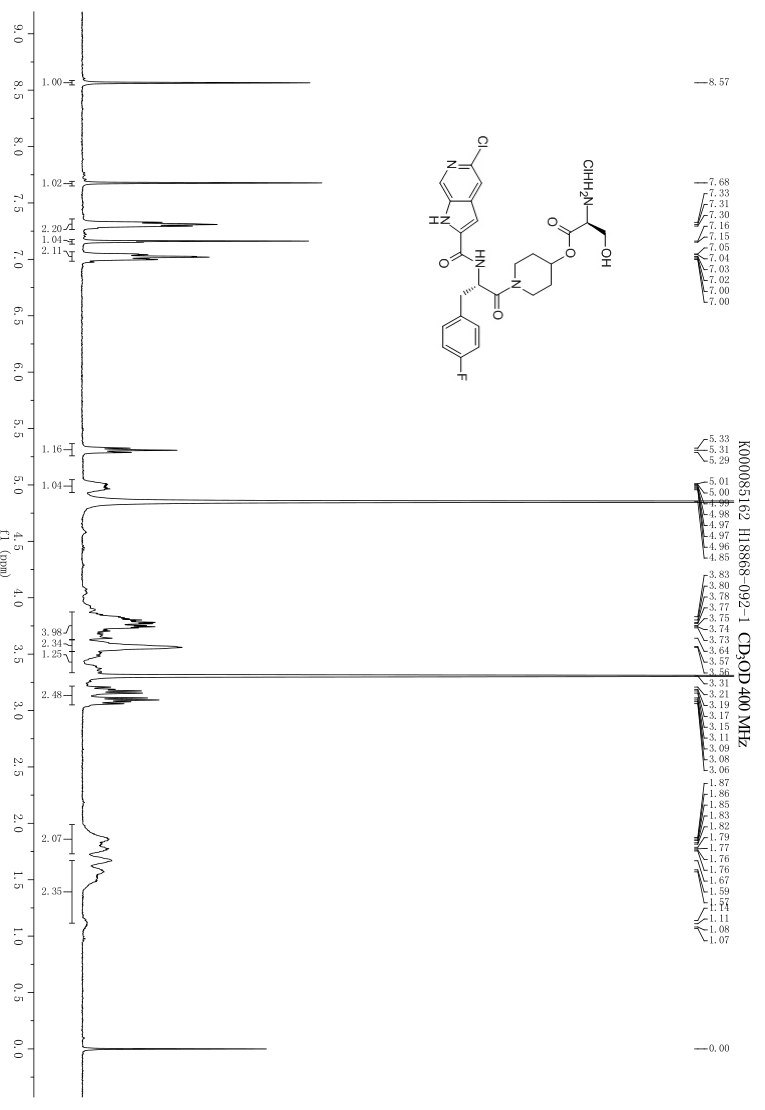


# Copy of ^13^C NMR of 27

#
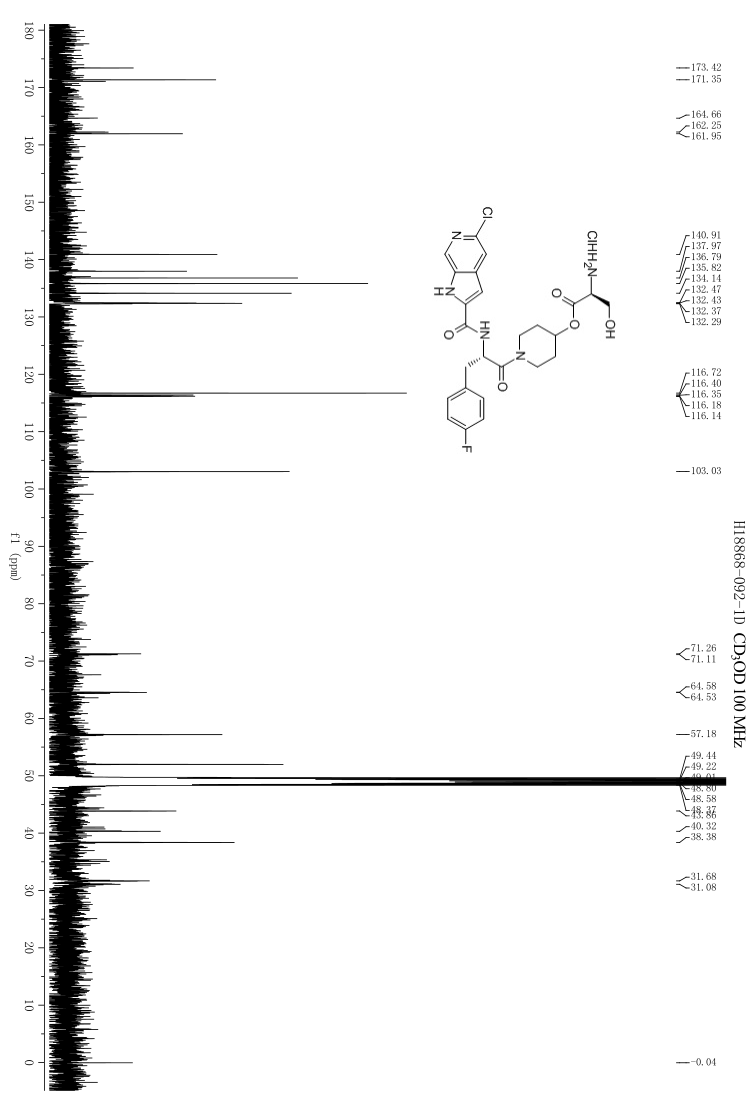


# Copy of ^1^H NMR of 5

#
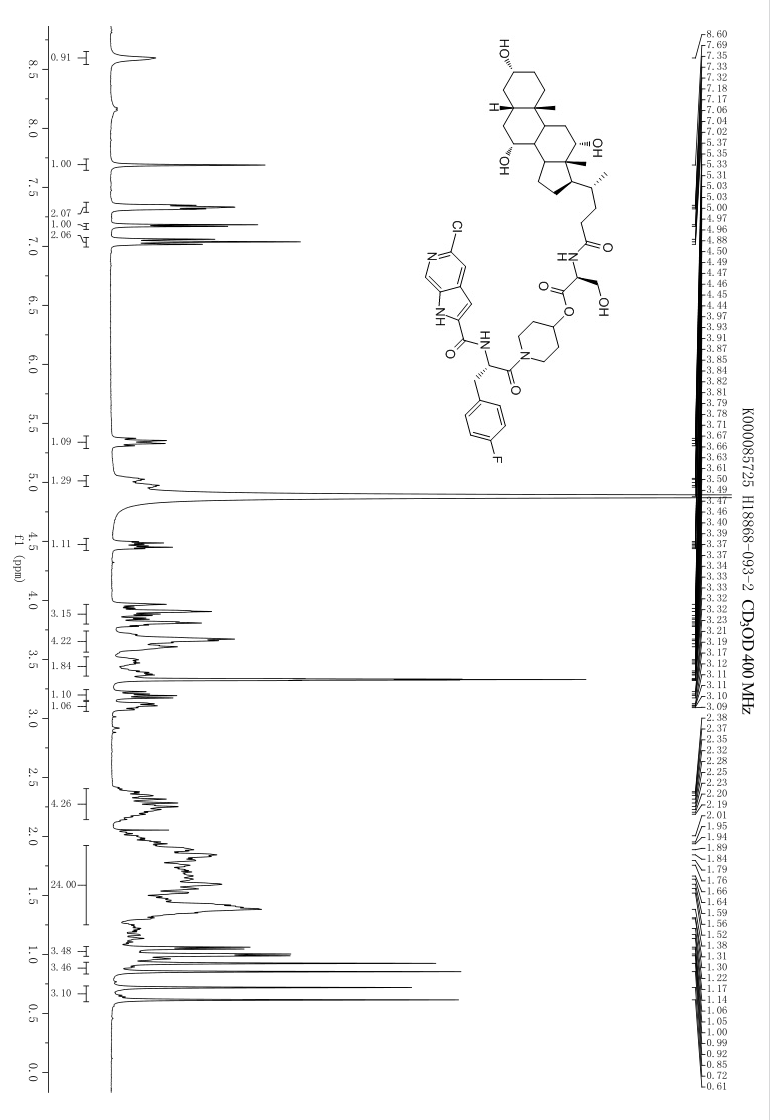


# Copy of ^13^C NMR of 5

#
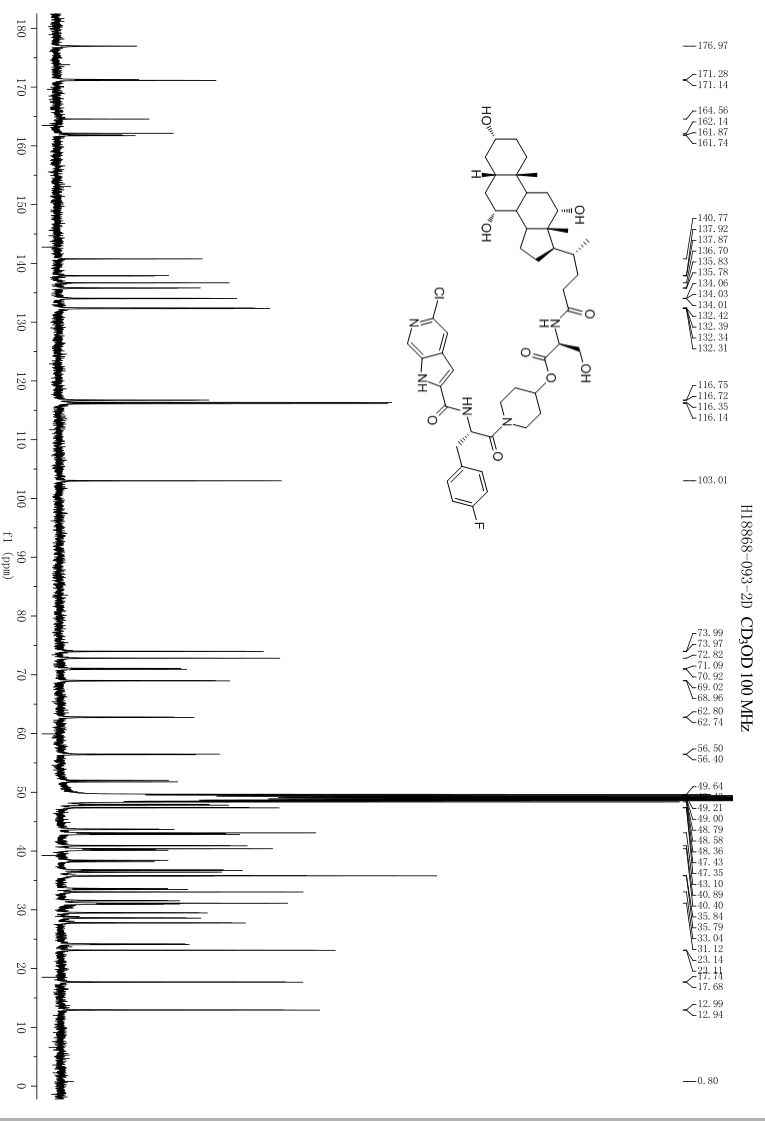


# Copy of ^1^H NMR of 28

#
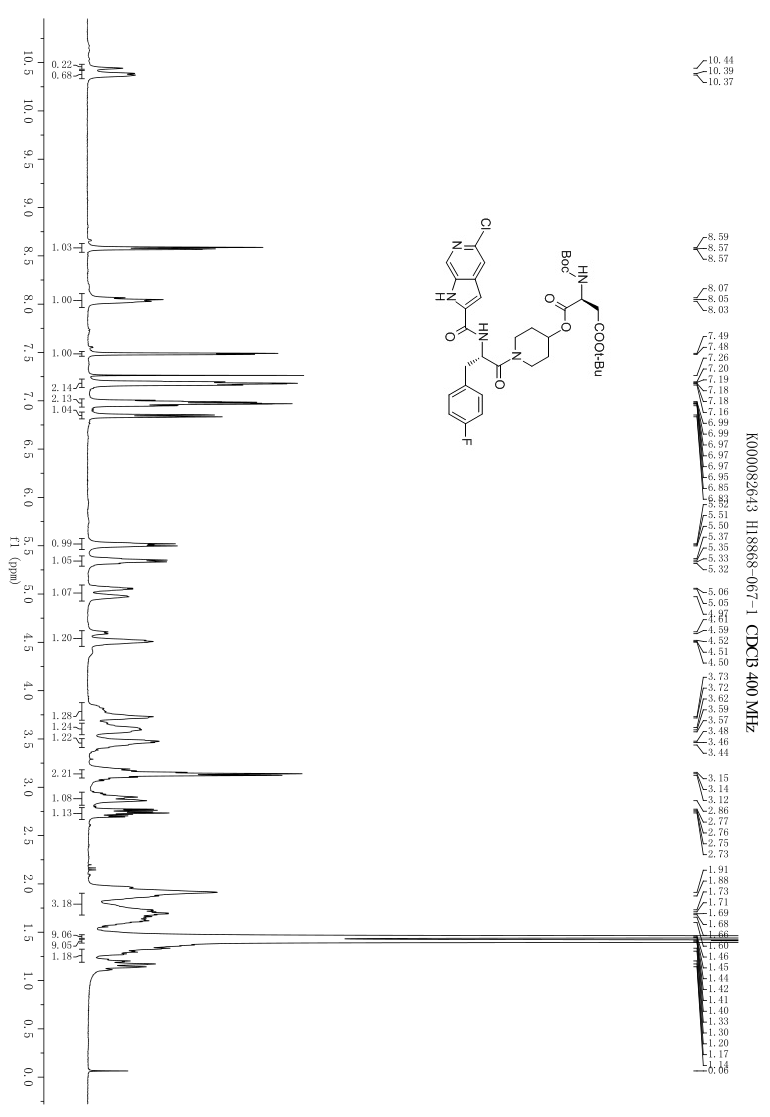


# Copy of ^13^C NMR of 28

#
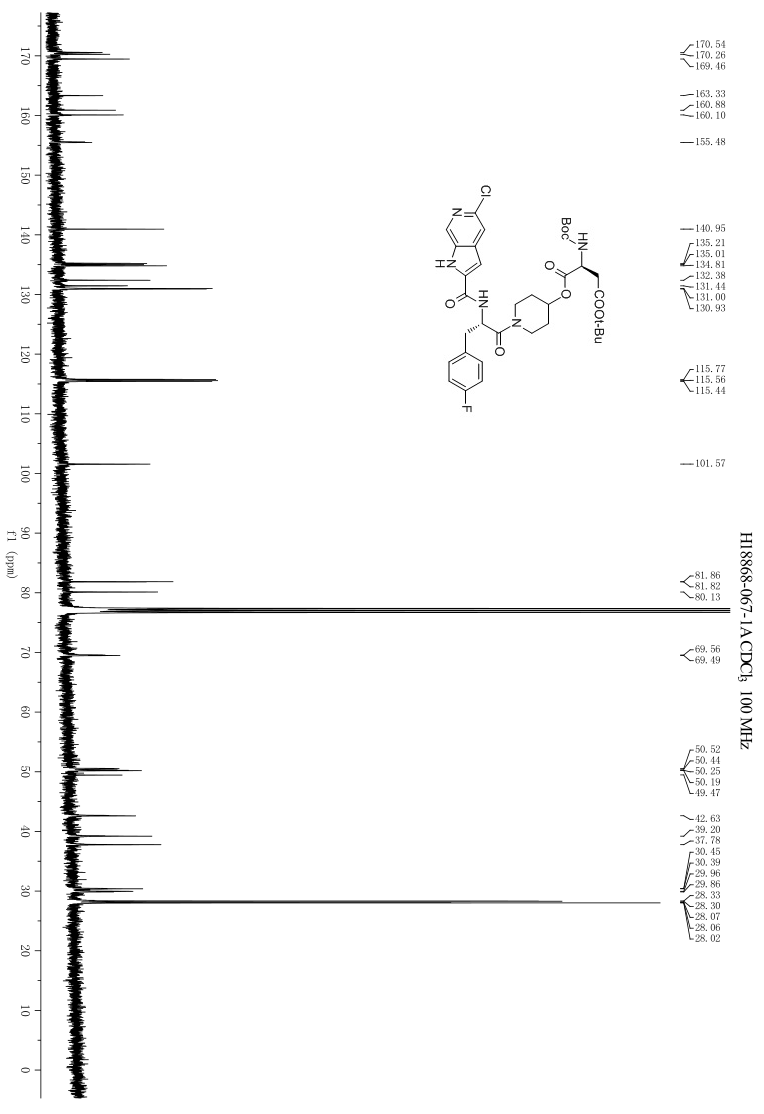


# Copy of ^1^H NMR of 6

#
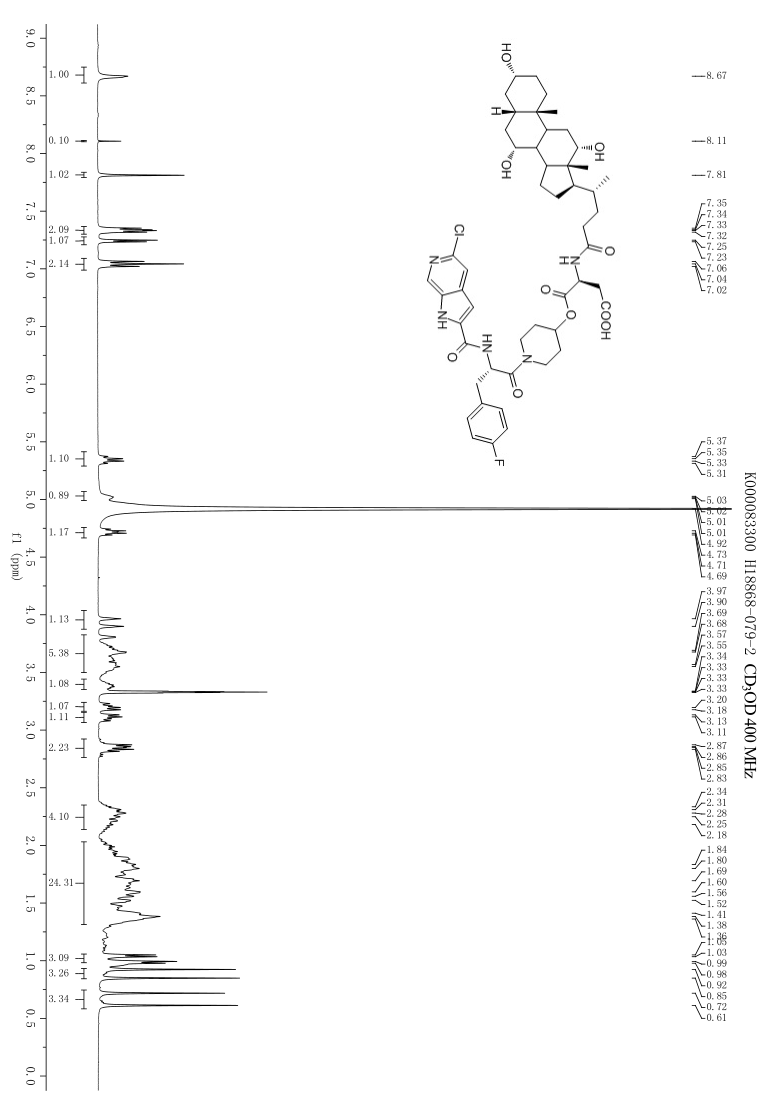


# Copy of ^13^C NMR of 6

#
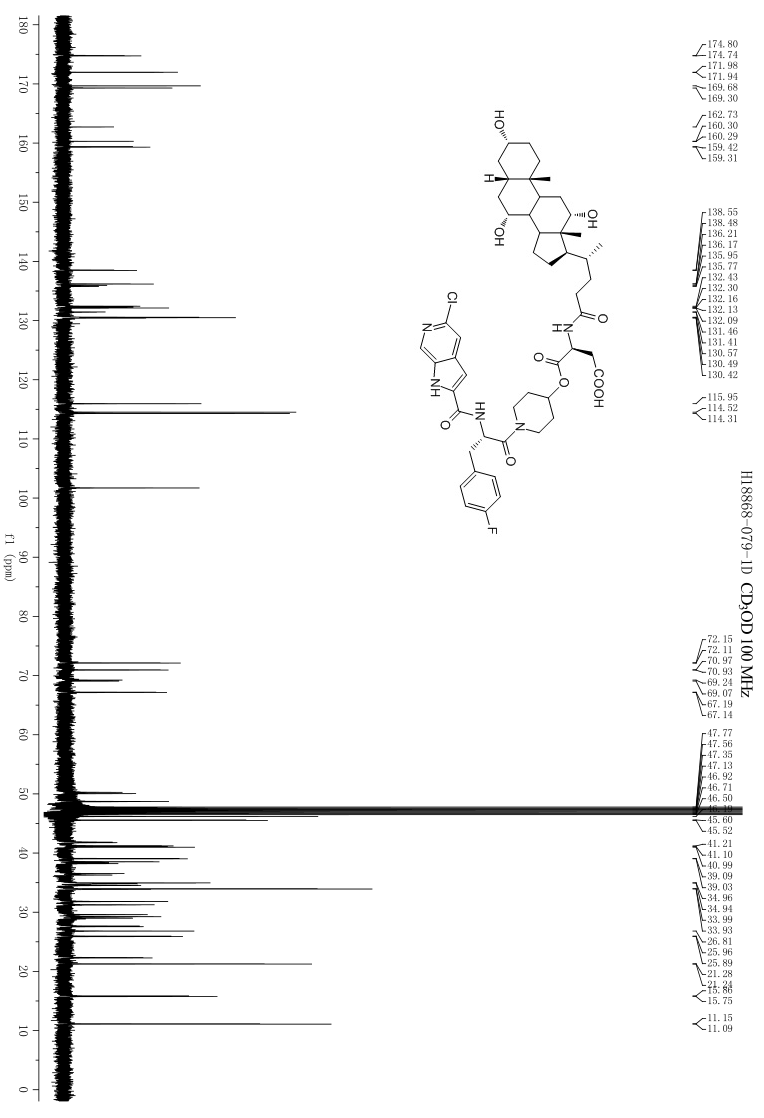


# Copy of ^1^H NMR of 31

#
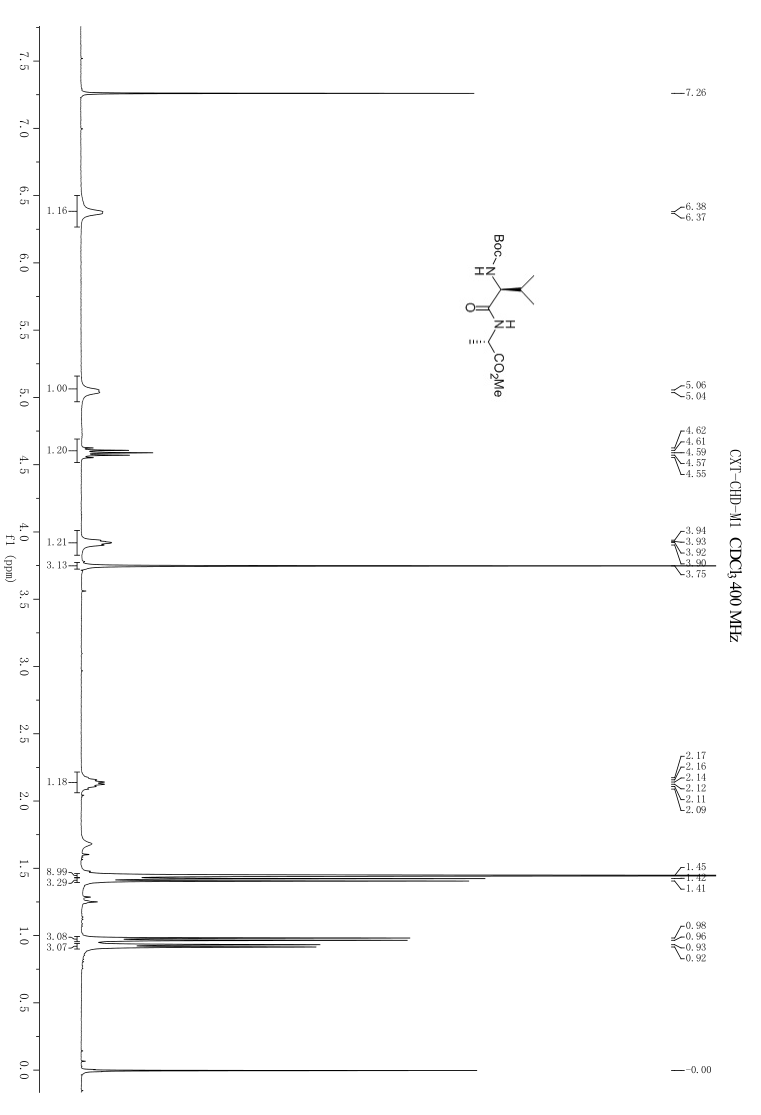


# Copy of ^13^C NMR of 31

#
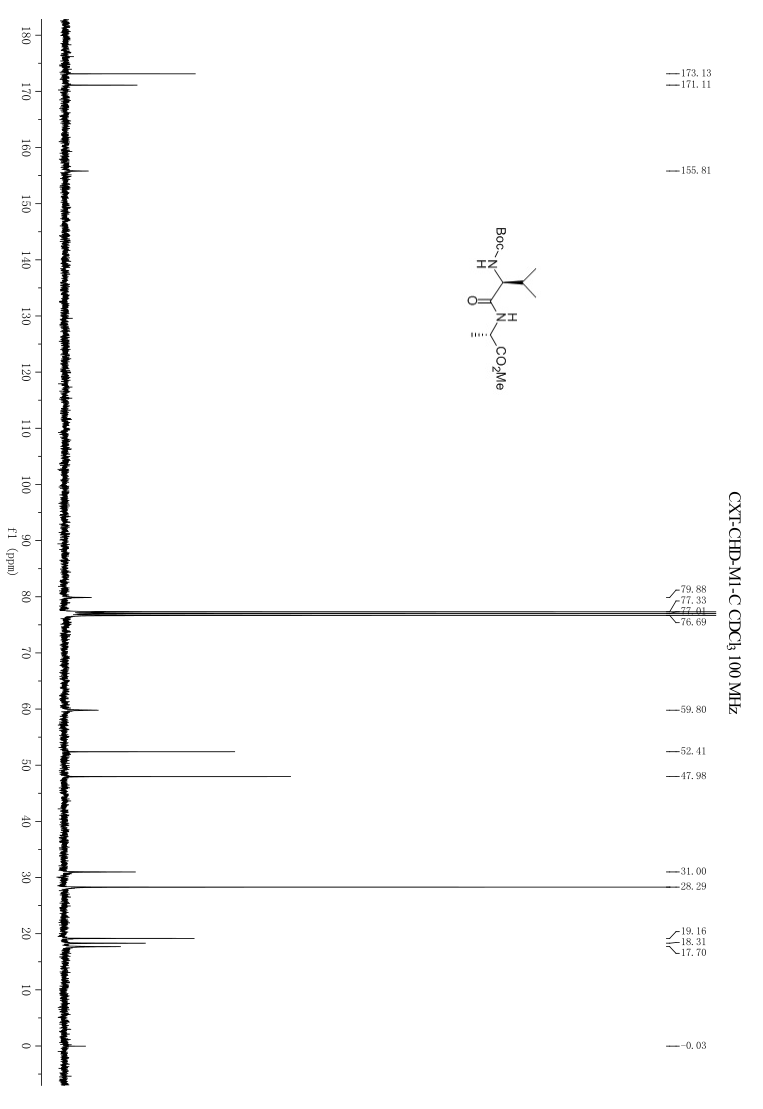


# Copy of ^1^H NMR of 32

#
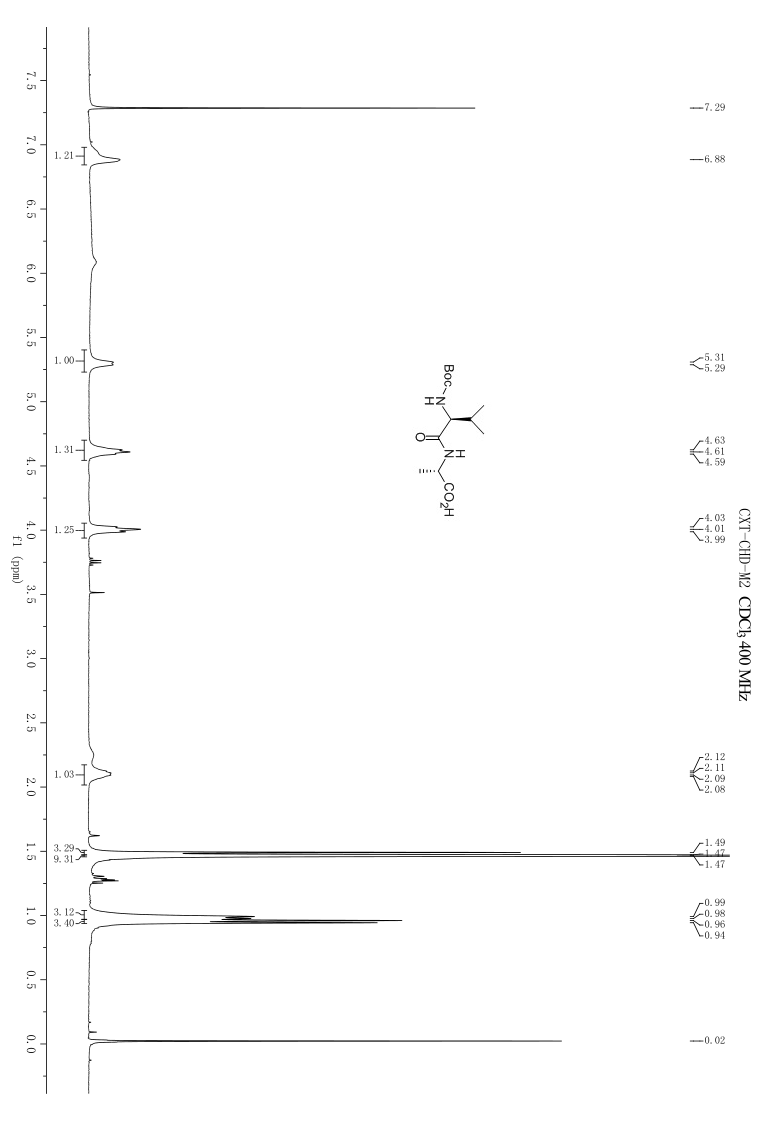


# Copy of ^13^C NMR of 32

#
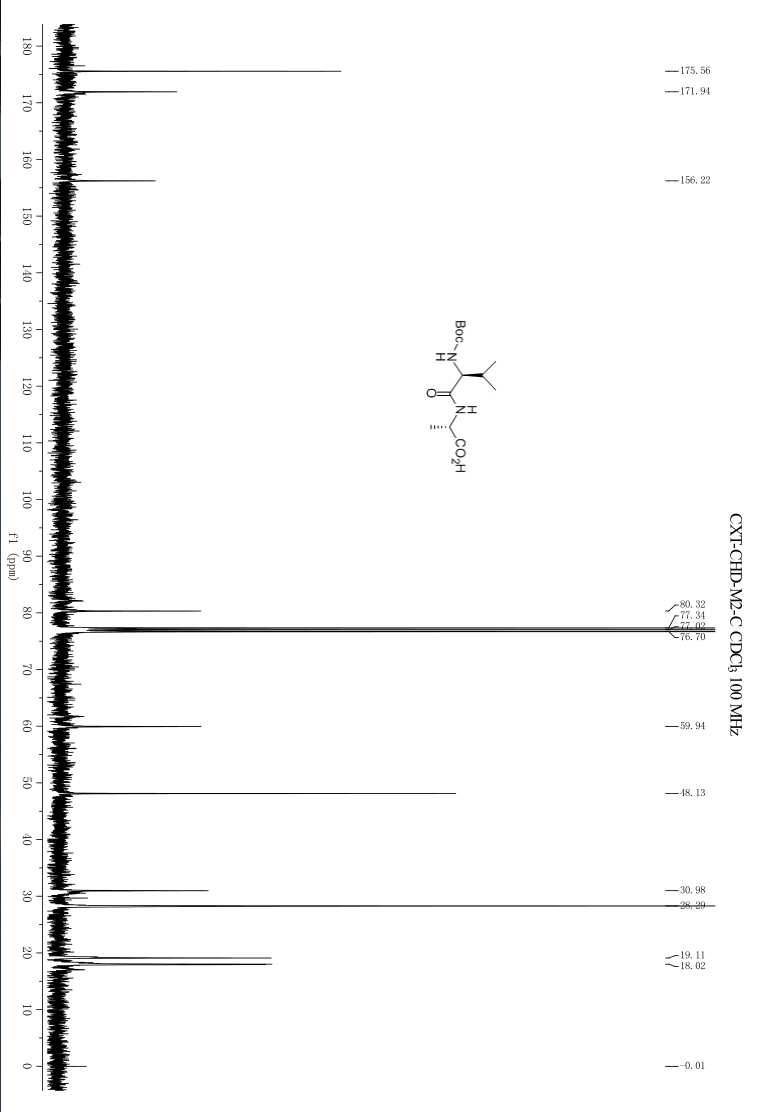


# Copy of ^1^H NMR of 33

#
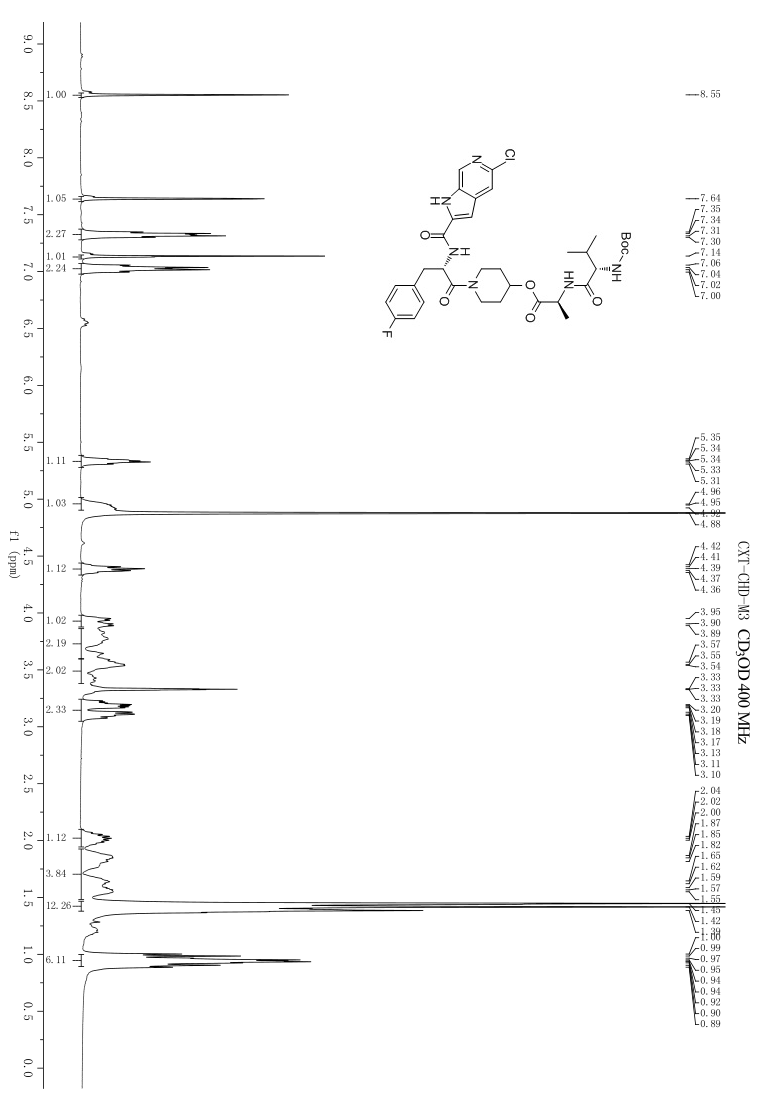


# Copy of ^13^C NMR of 33


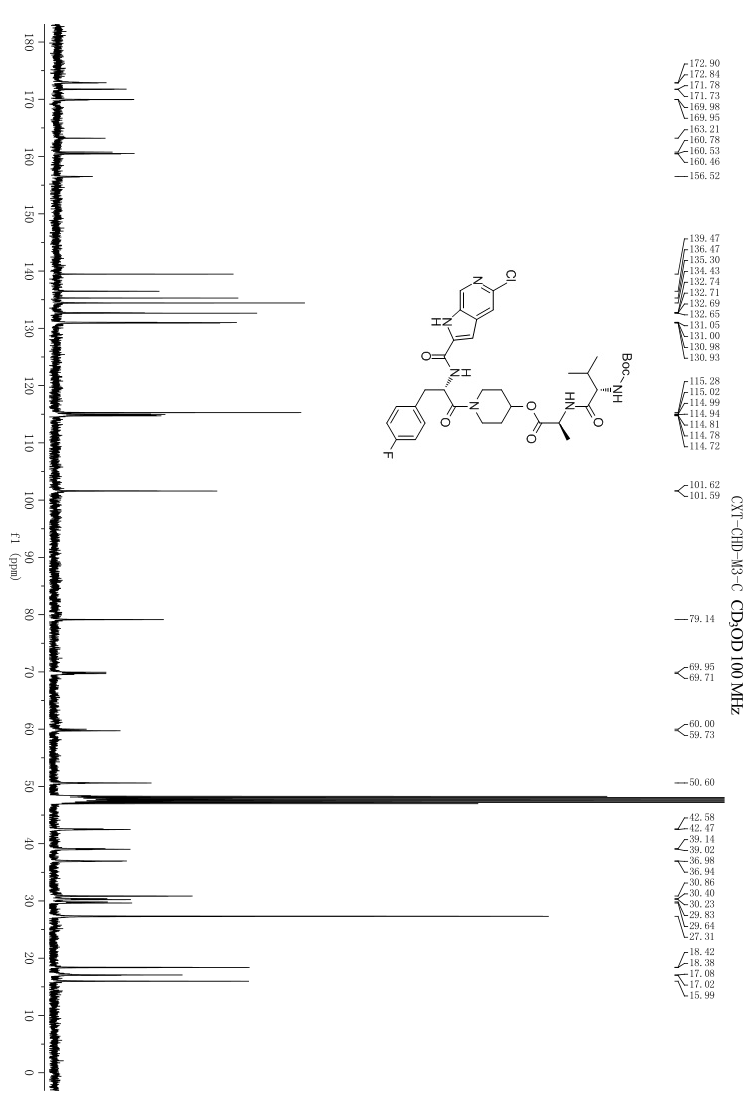


# Copy of ^1^H NMR of 7

#
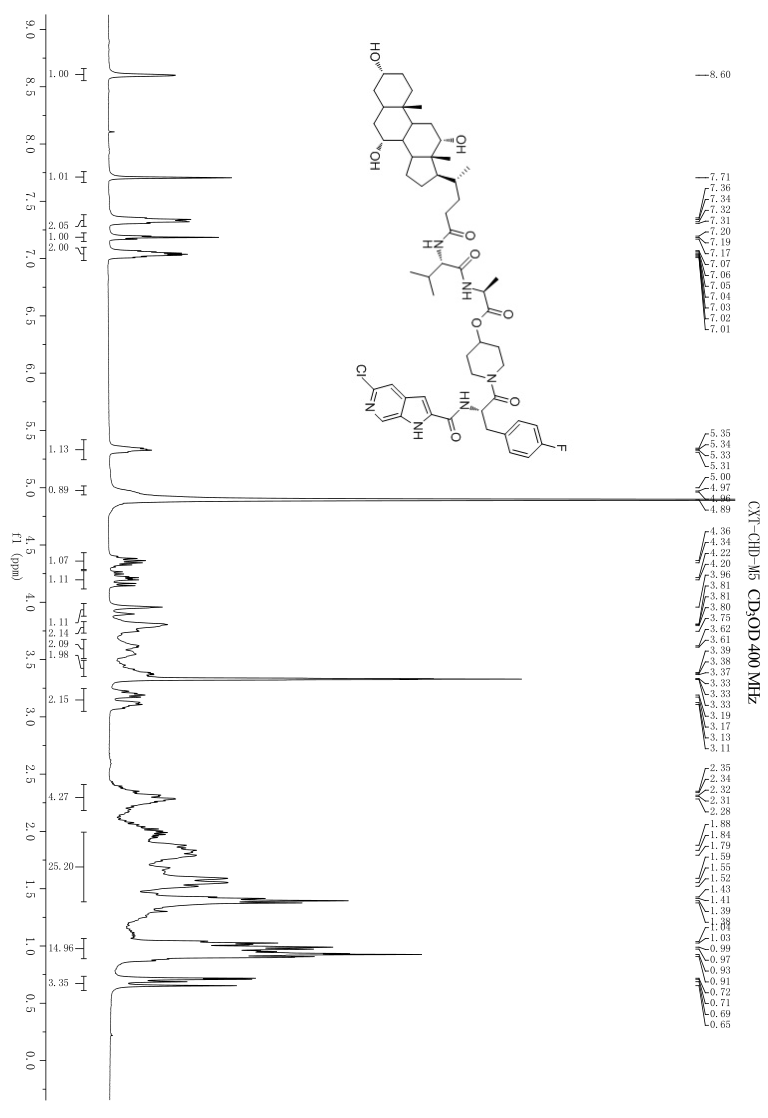


# Copy of ^13^C NMR of 7

#
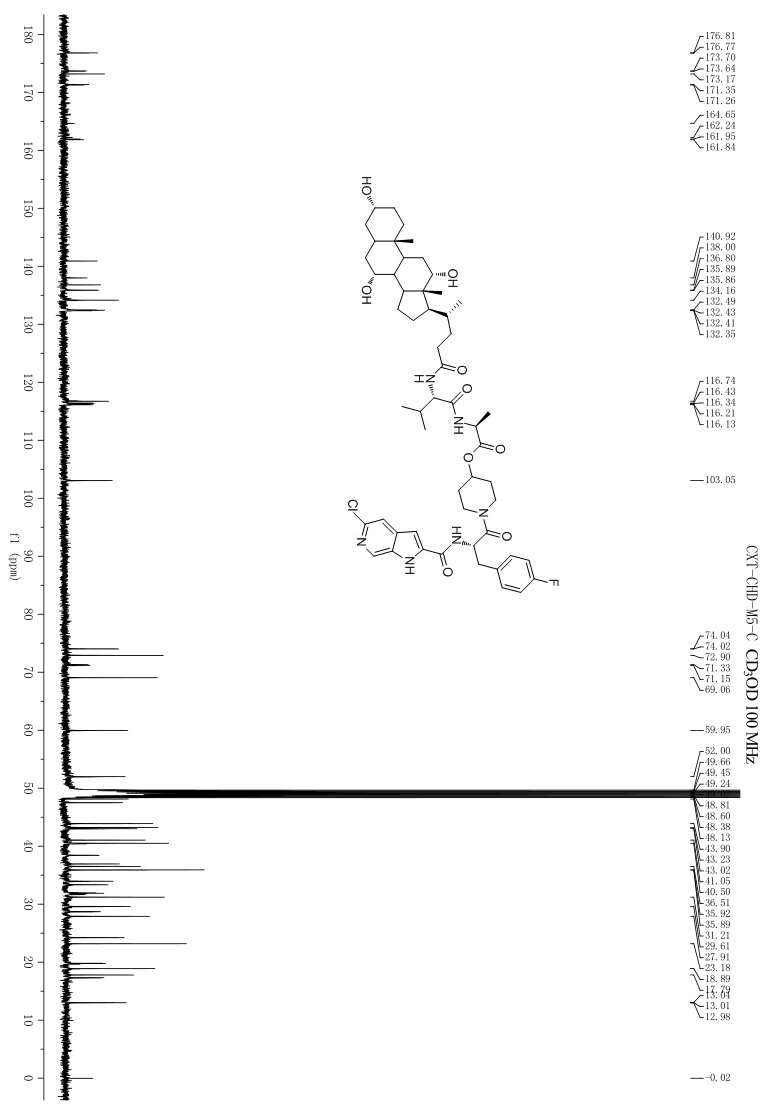


# Copy of ^1^H NMR of 36

#
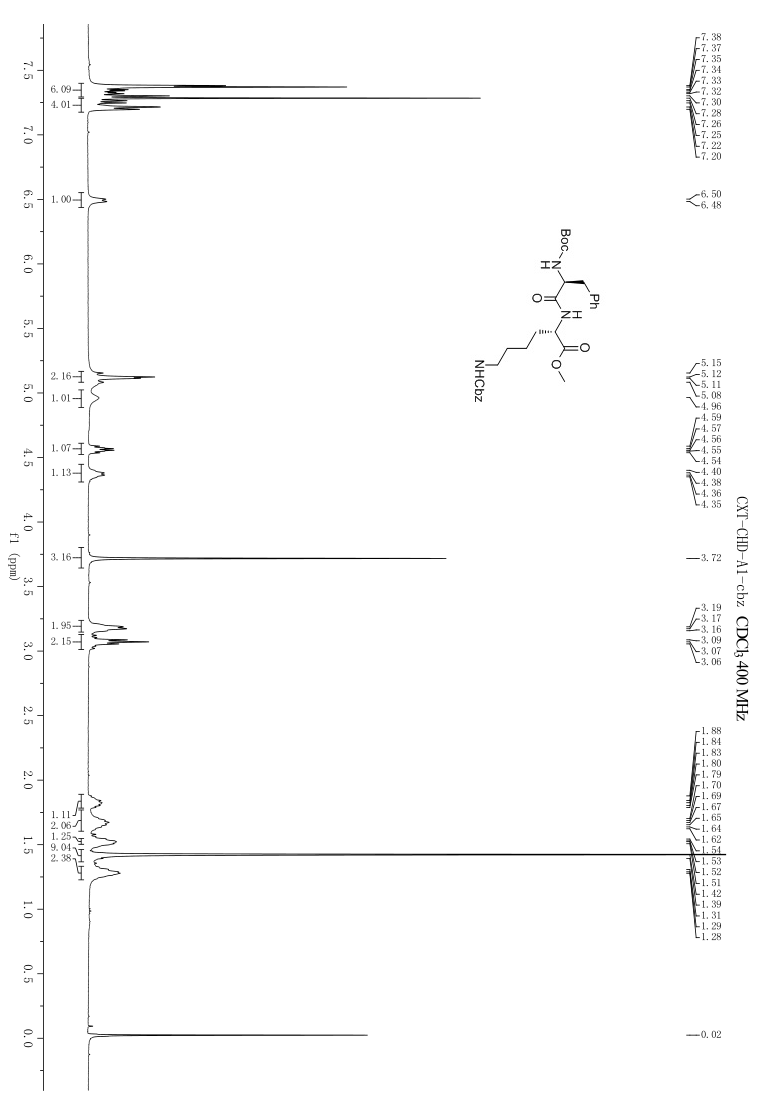


# Copy of ^13^C NMR of 36

#
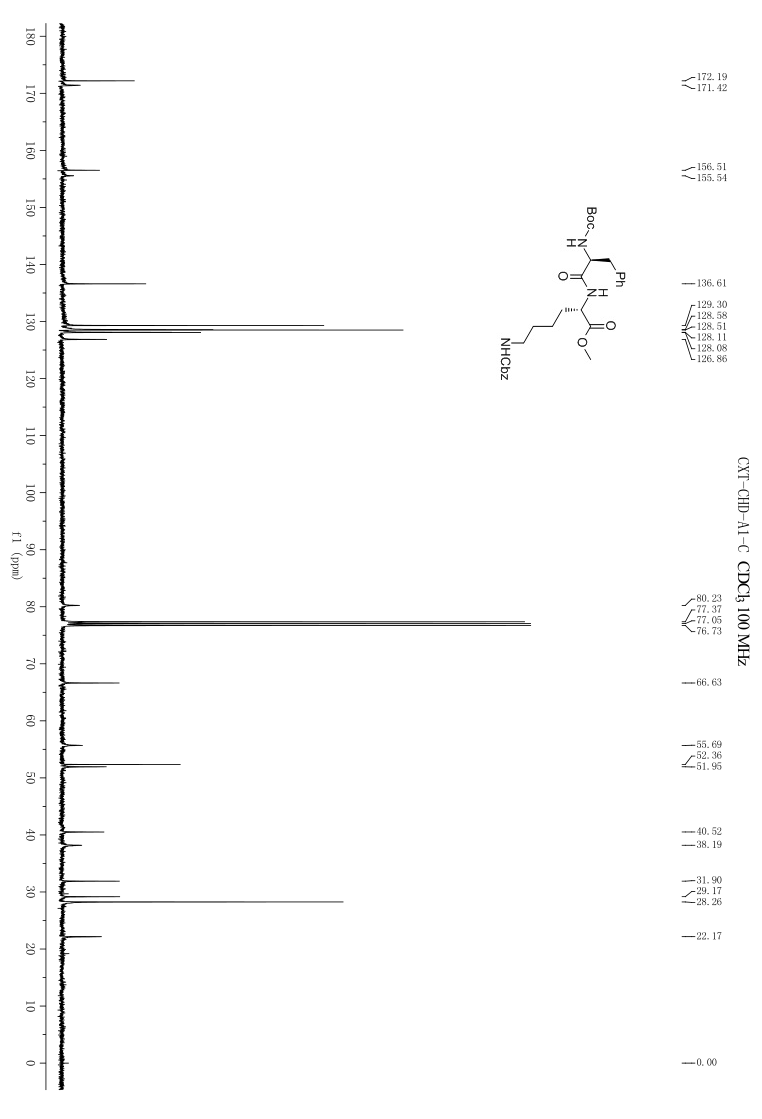


# Copy of ^1^H NMR of 37

#
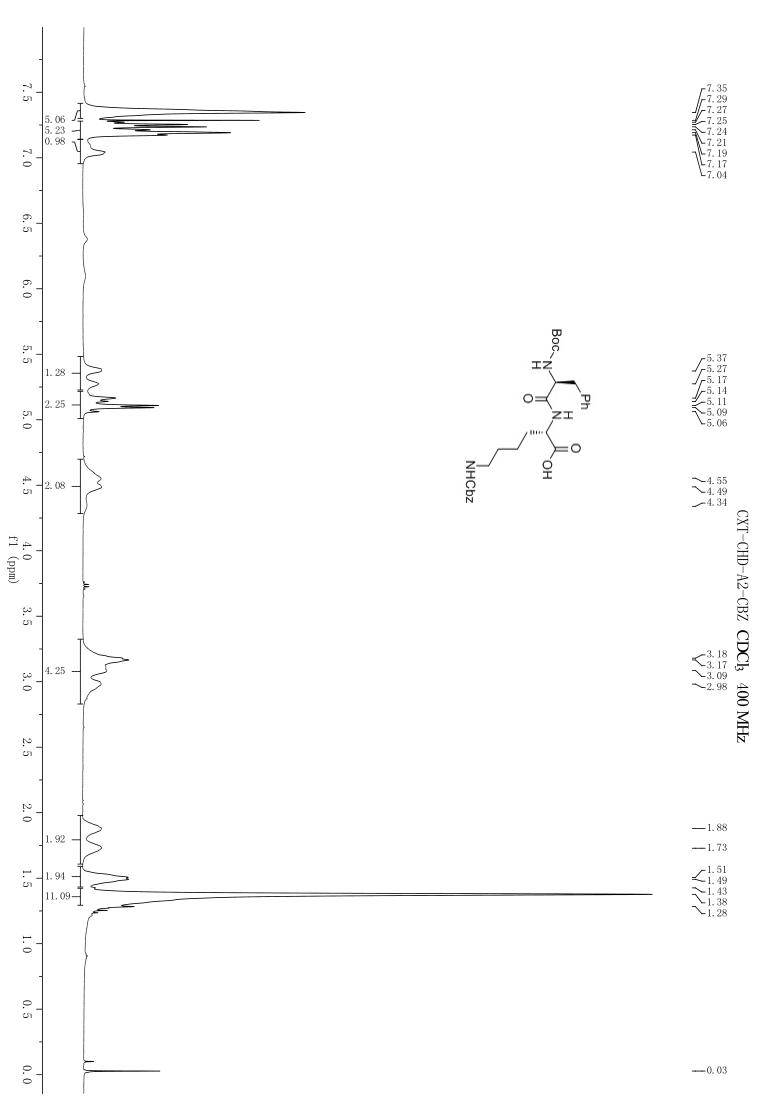


# Copy of ^13^C NMR of 37

#
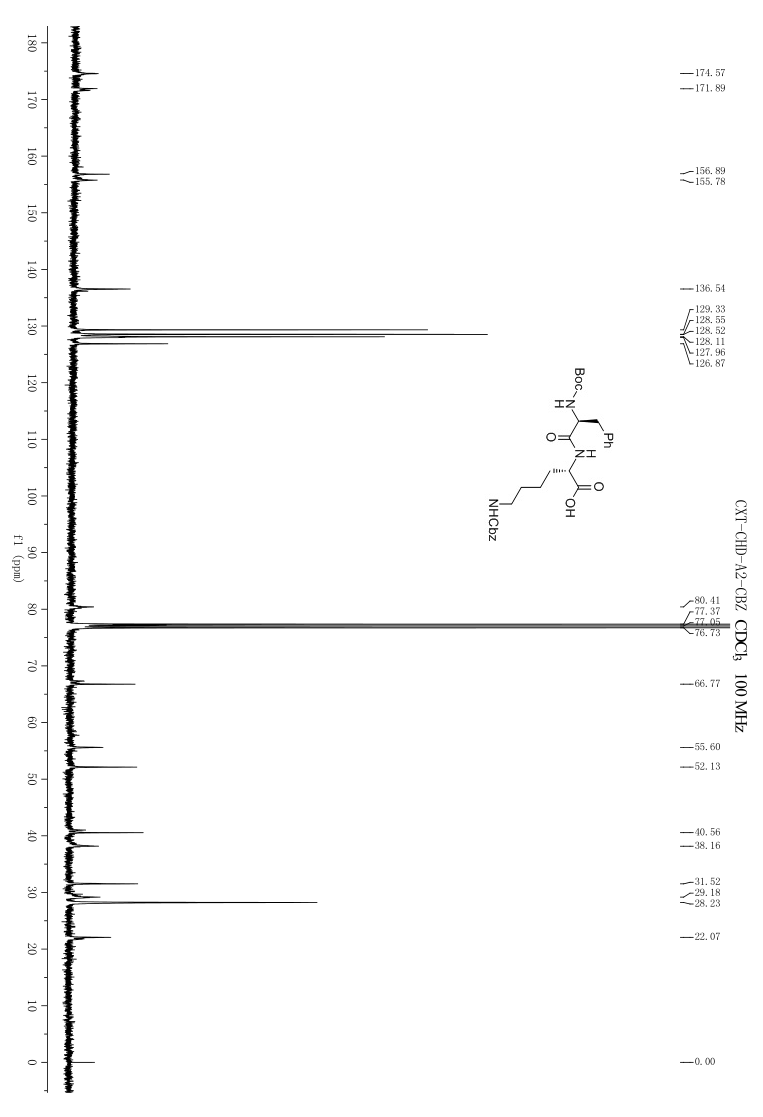


# Copy of ^1^H NMR of 40


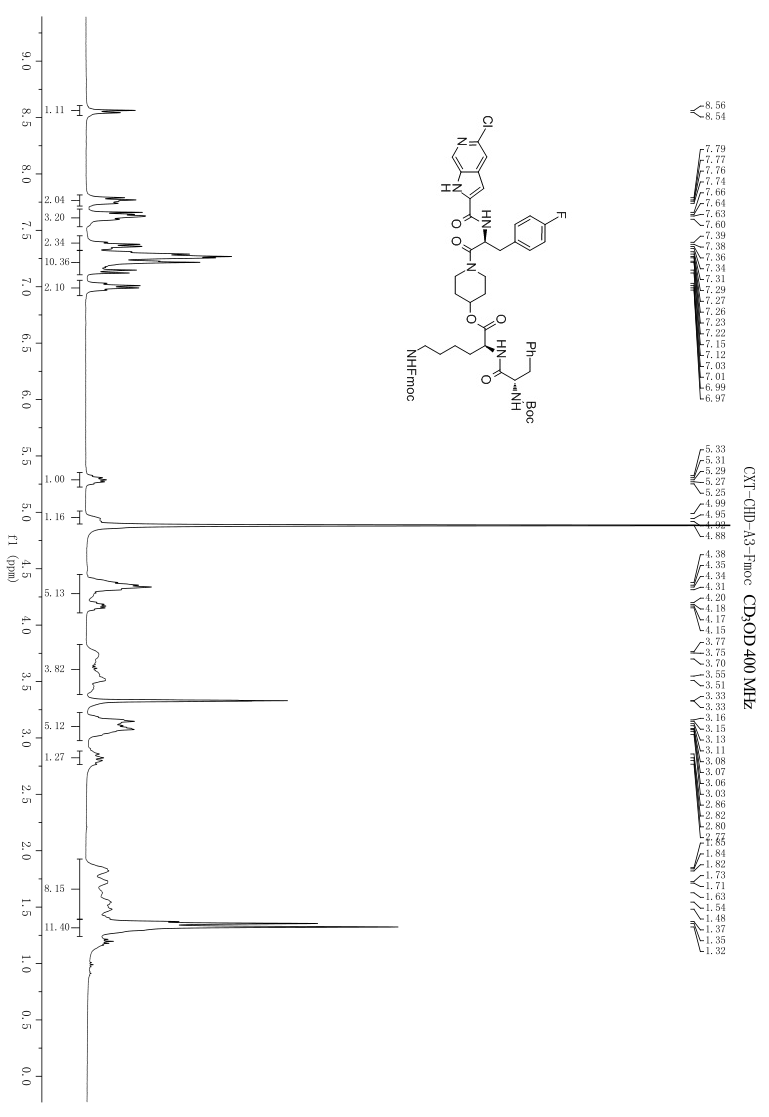


# Copy of ^13^C NMR of 40

#
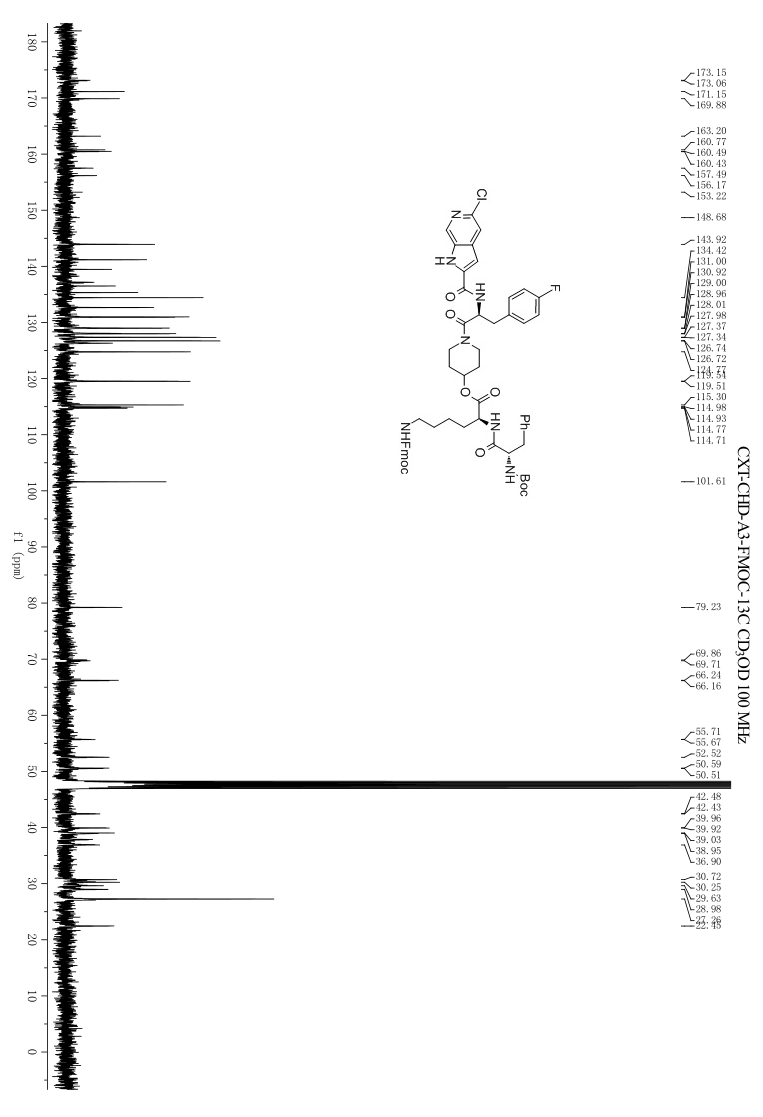


# Copy of ^1^H NMR of 42

#
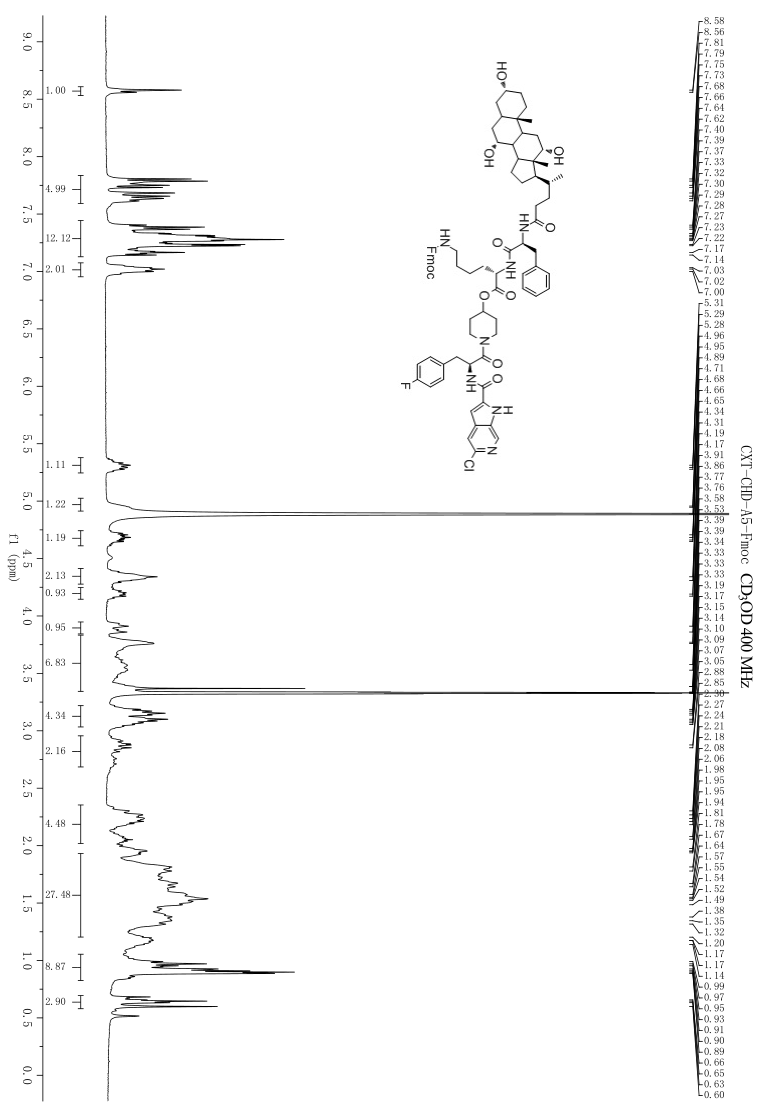


# Copy of ^13^C NMR of 42

#
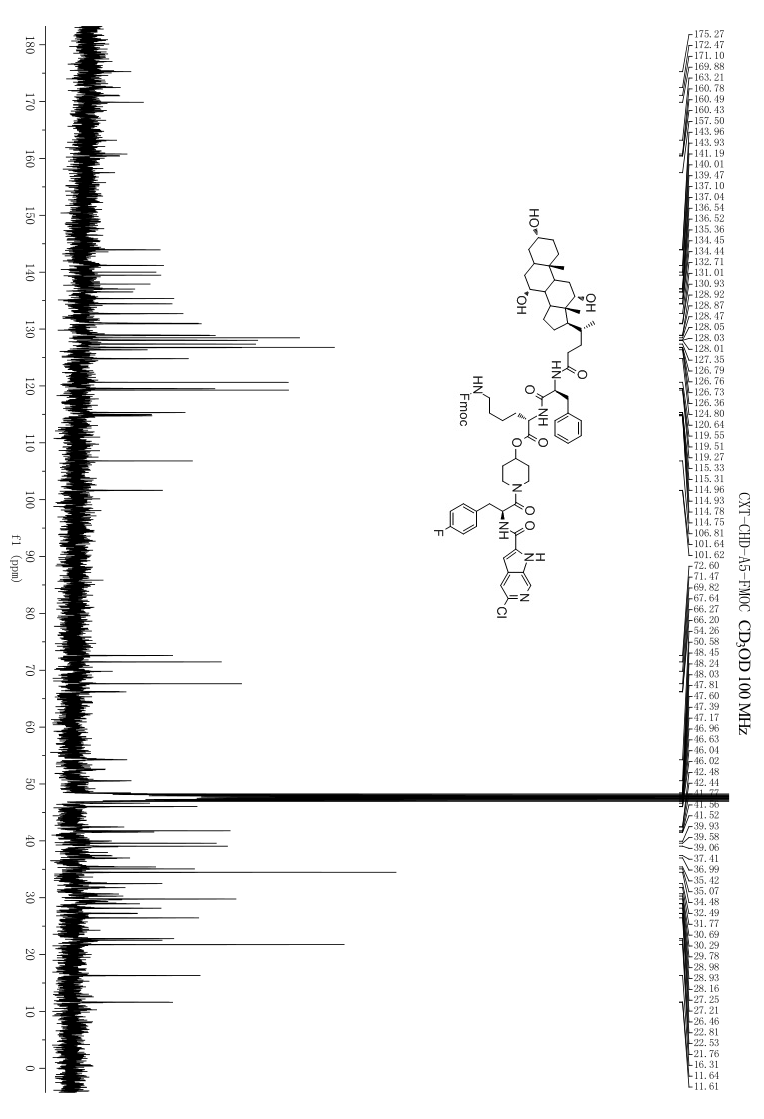


# Copy of ^1^H NMR of 8

#
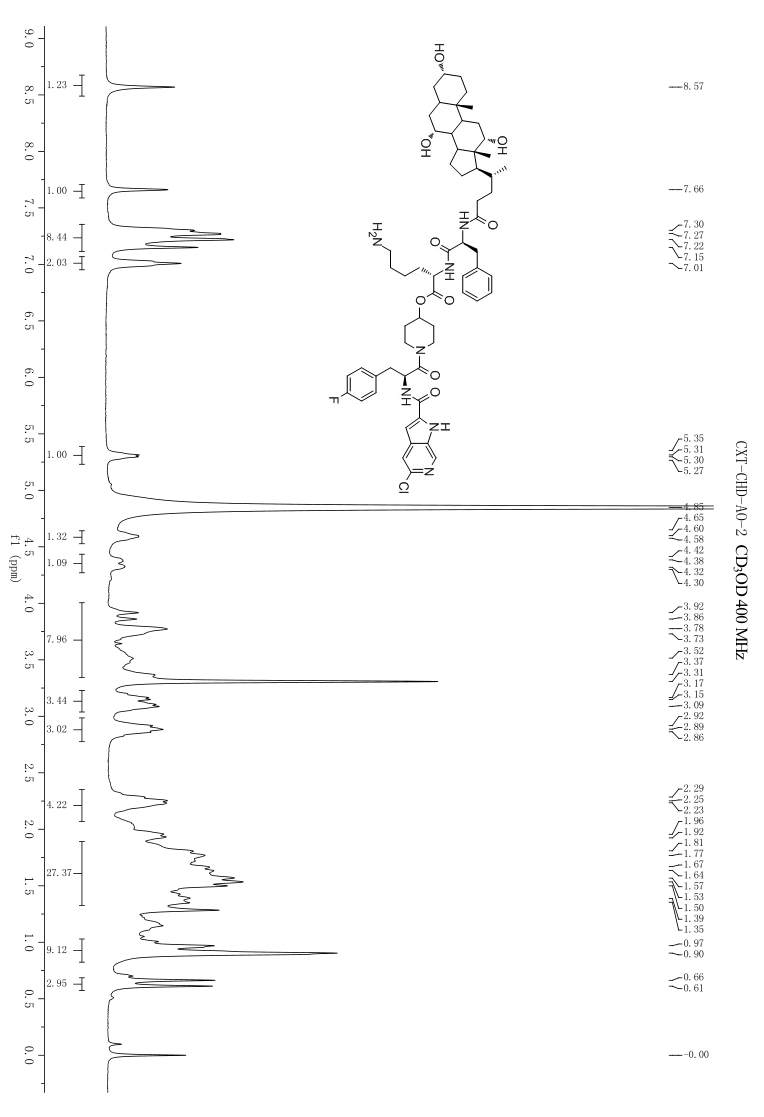


# Copy of ^13^C NMR of 8

#
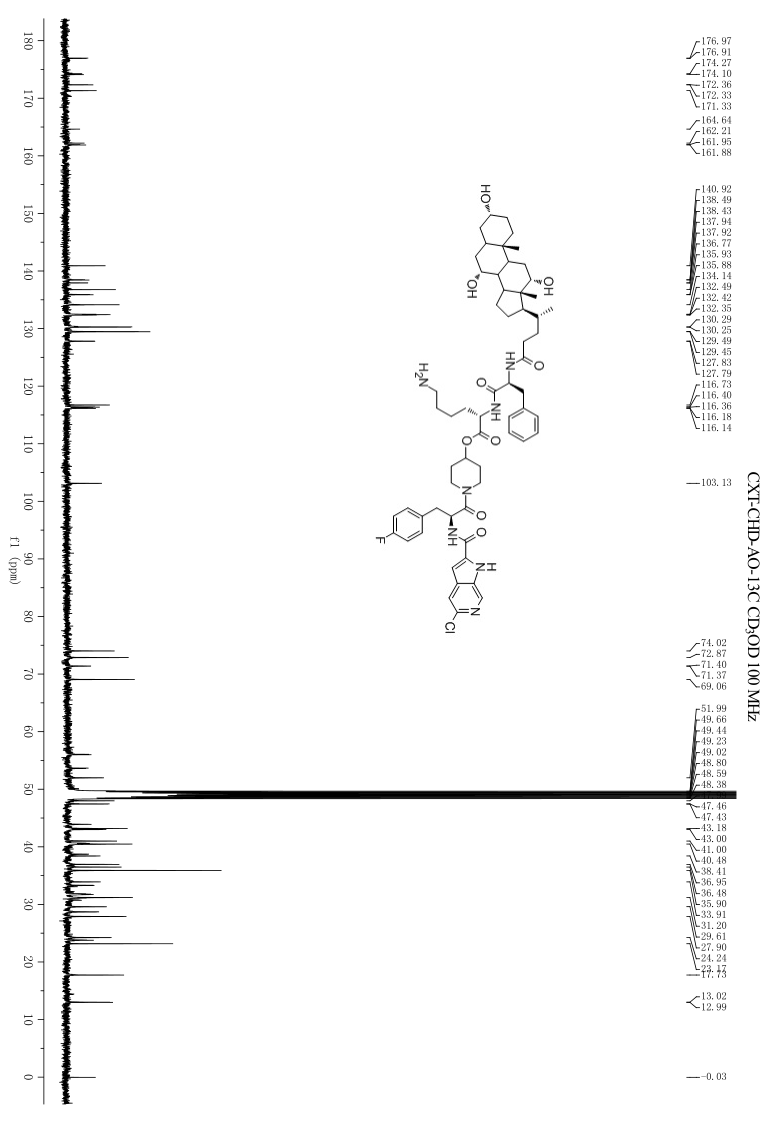


# Copy of ^1^H NMR of 46

**
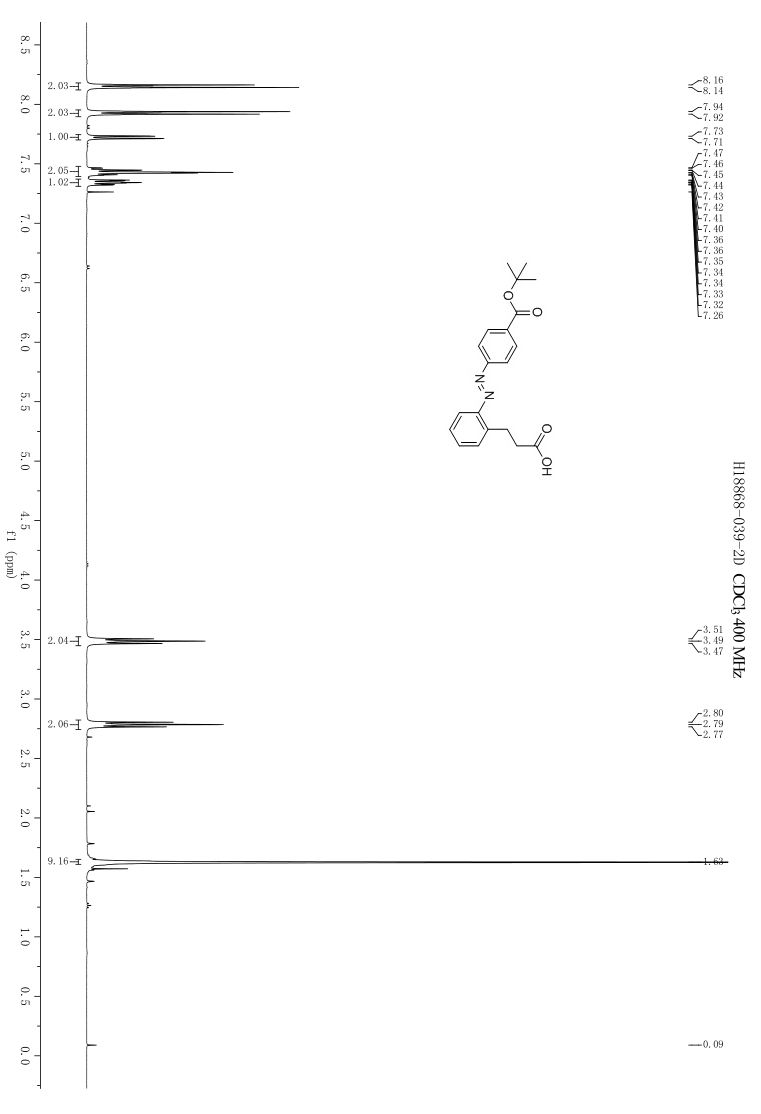
**

#
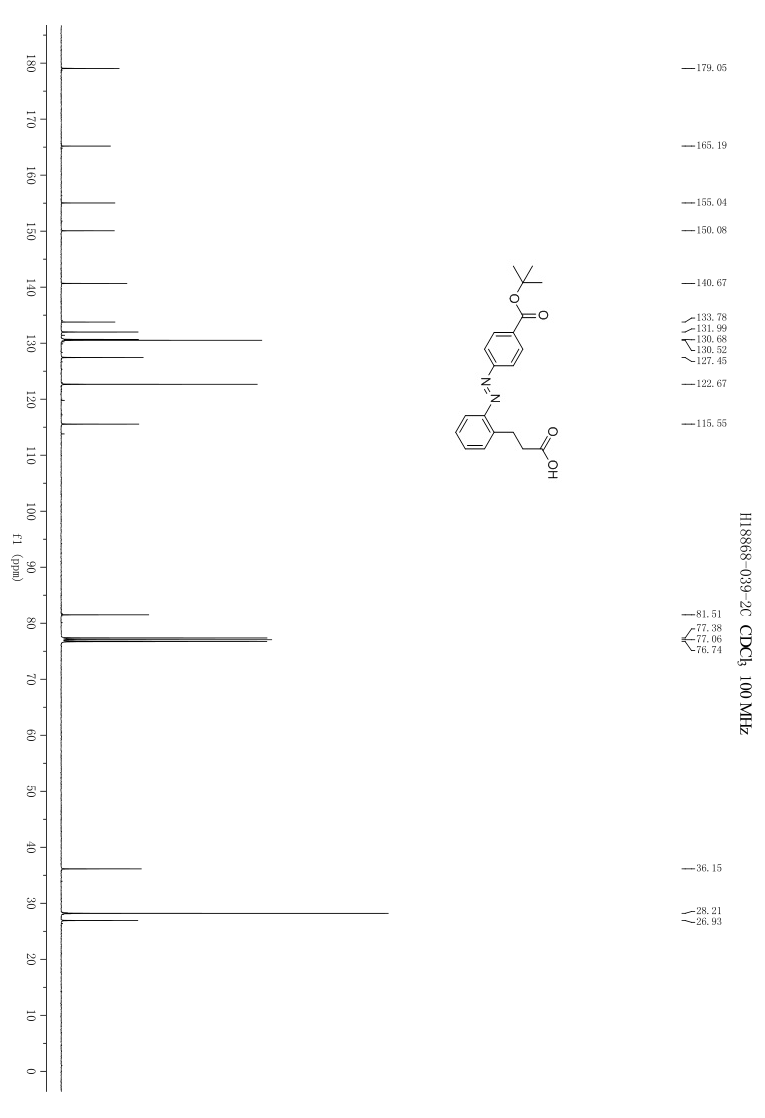
Copy of ^13^C NMR of 46

# Copy of ^1^H NMR of 47


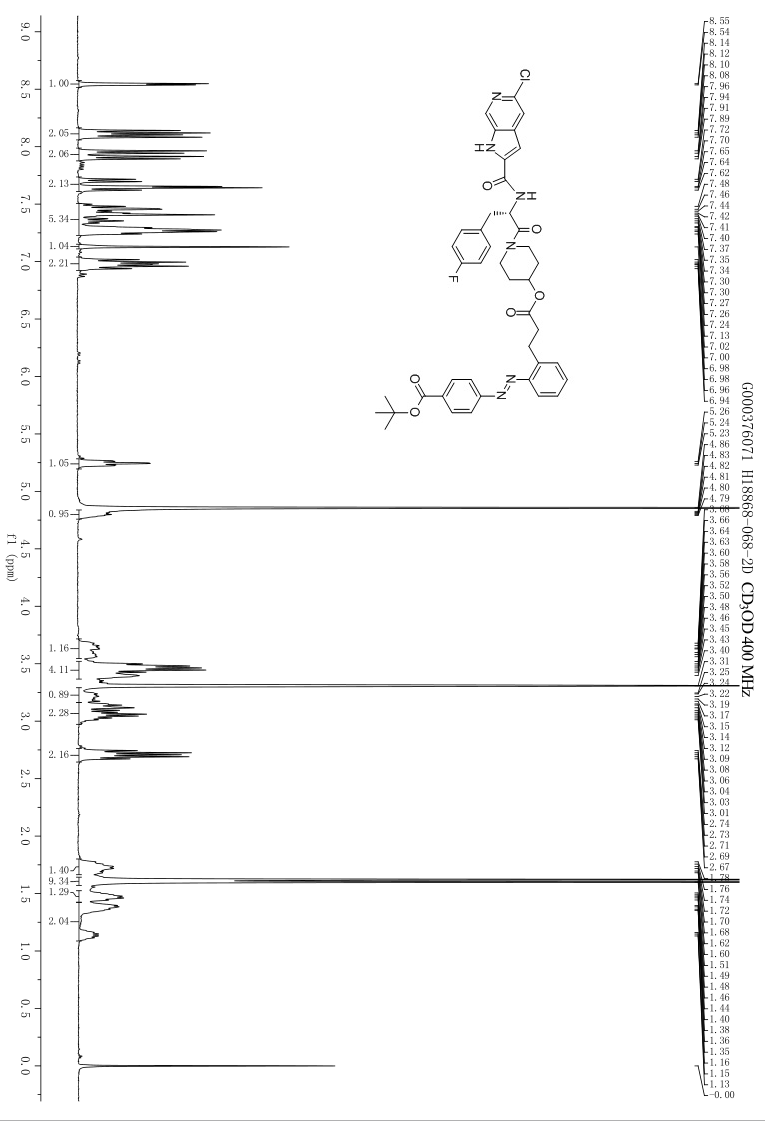


#
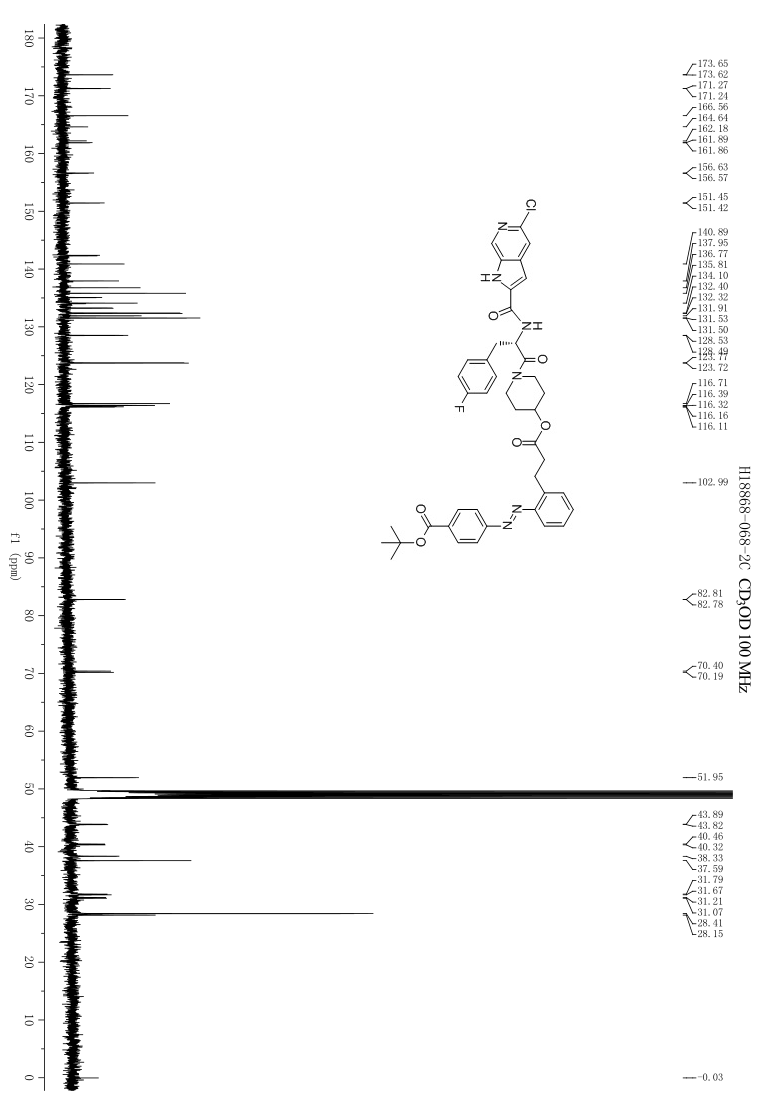
Copy of ^13^C NMR of 47

#
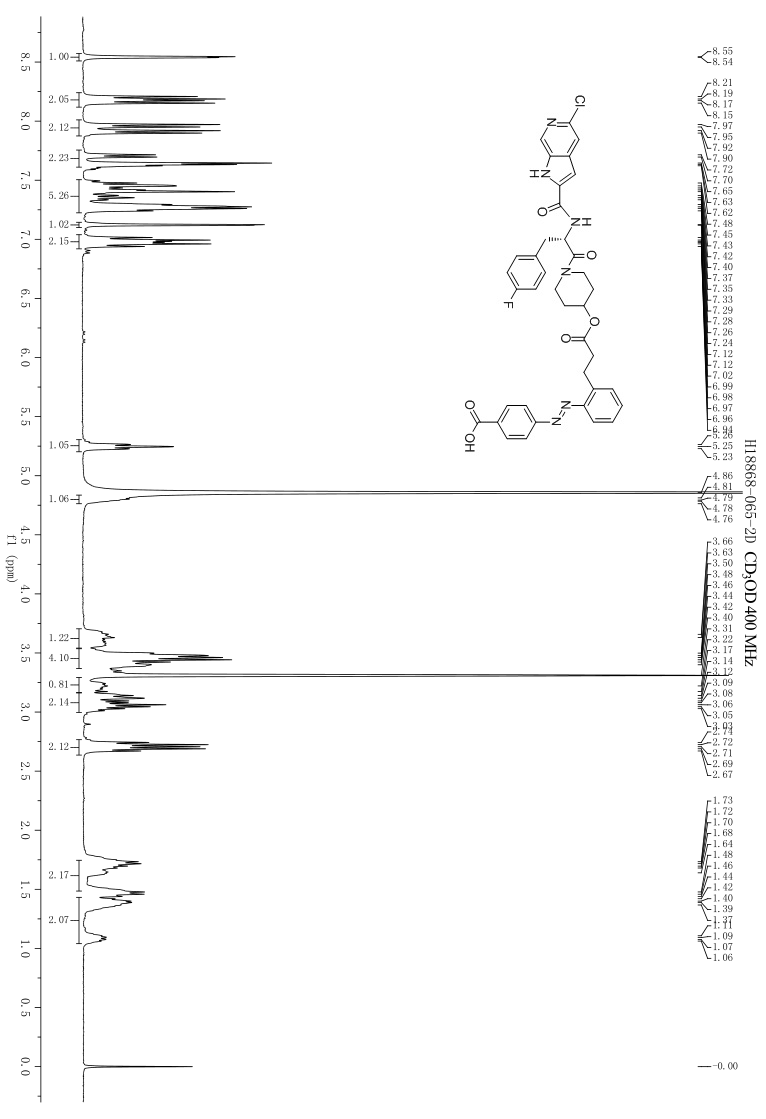
Copy of ^1^H NMR of 48

#
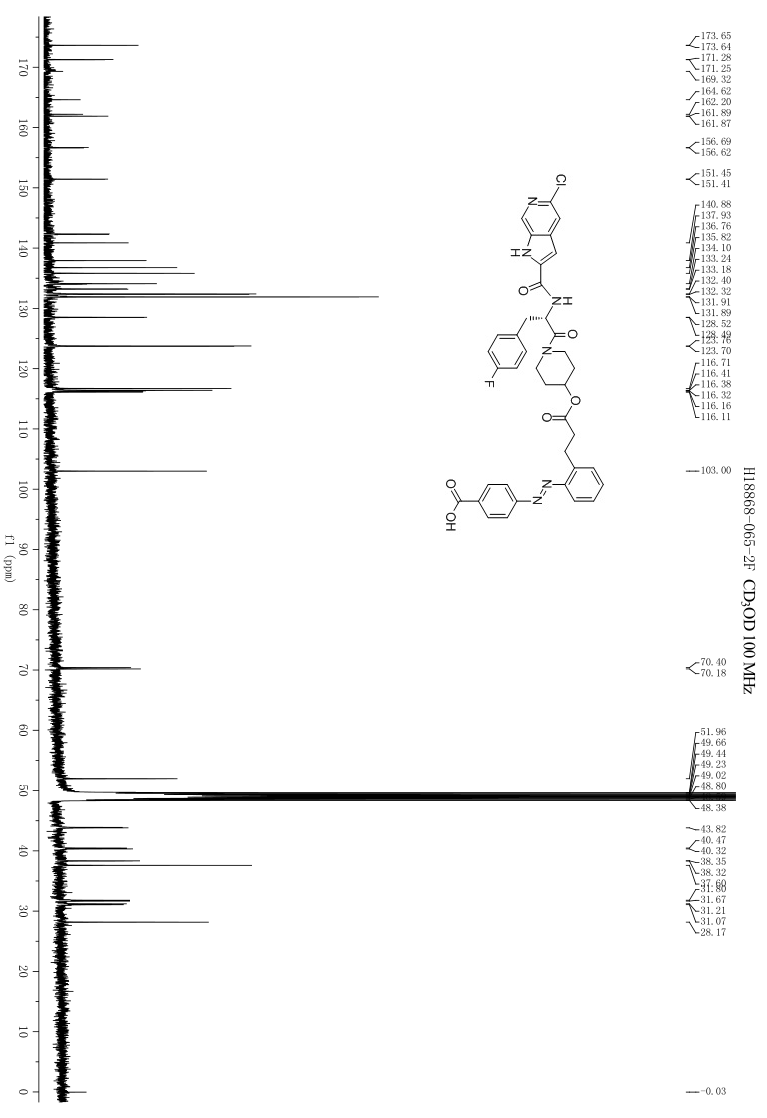
Copy of ^13^C NMR of 48

#
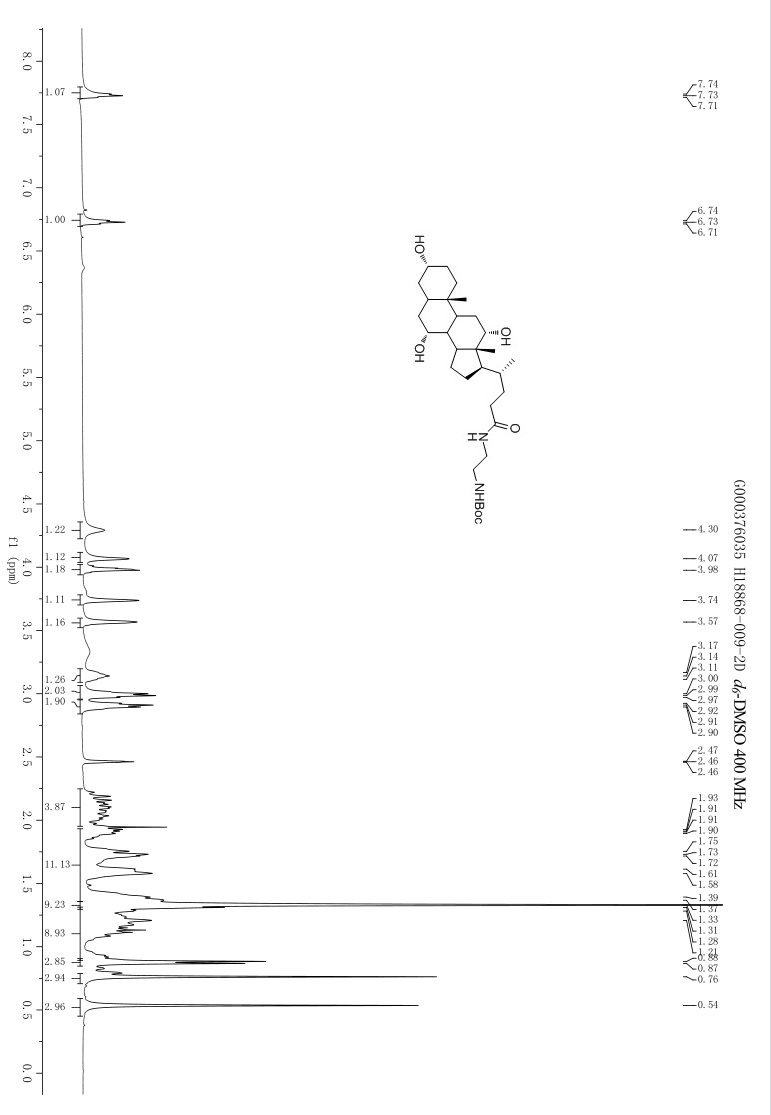
Copy of ^1^H NMR of 49

#
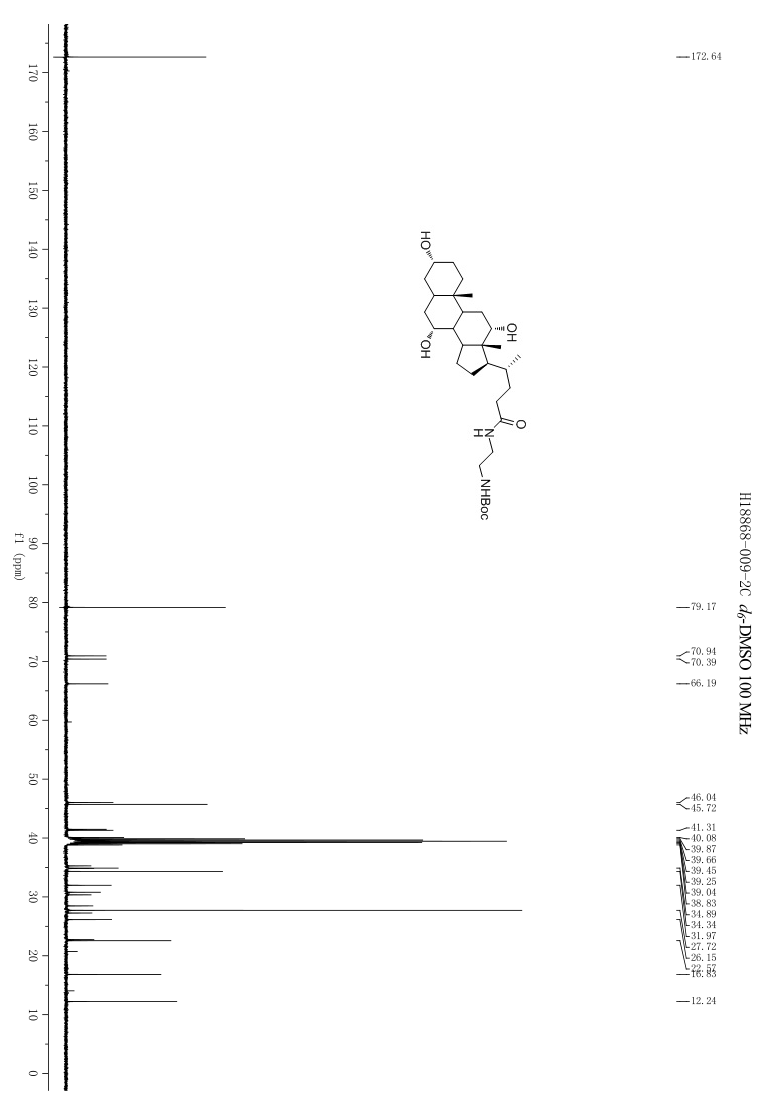
Copy of ^13^C NMR of 49

# Copy of ^1^H NMR of 50

#
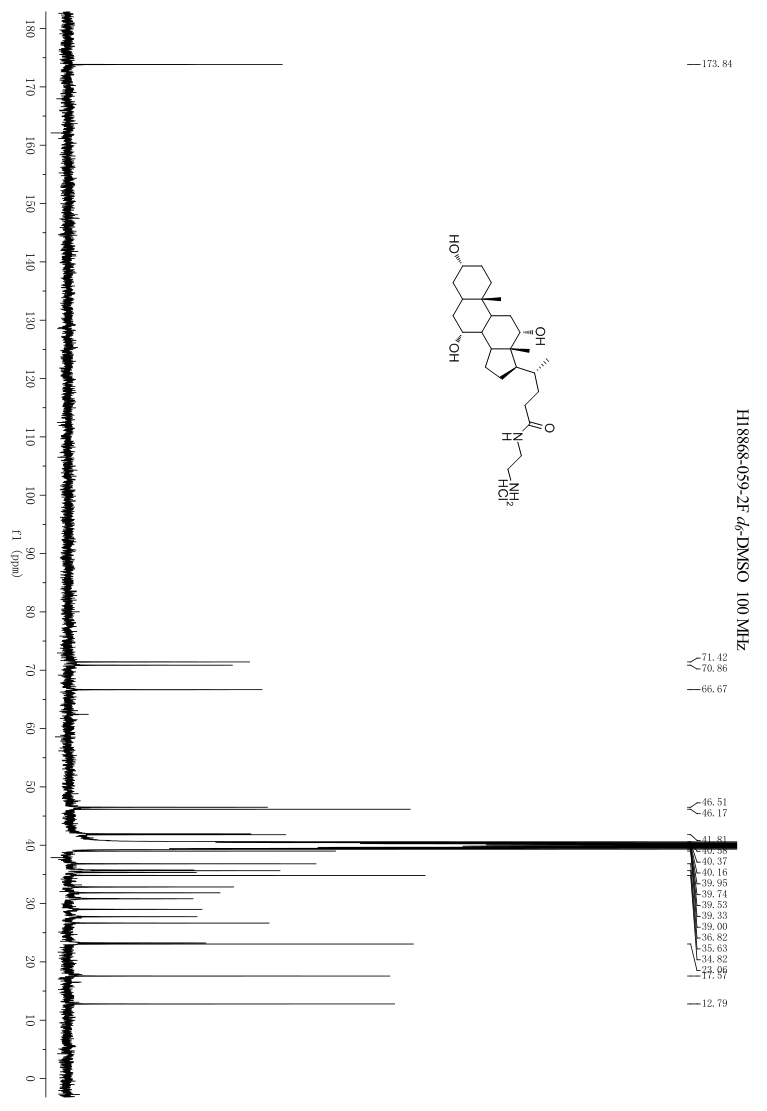
Copy of ^13^C NMR of 50

#
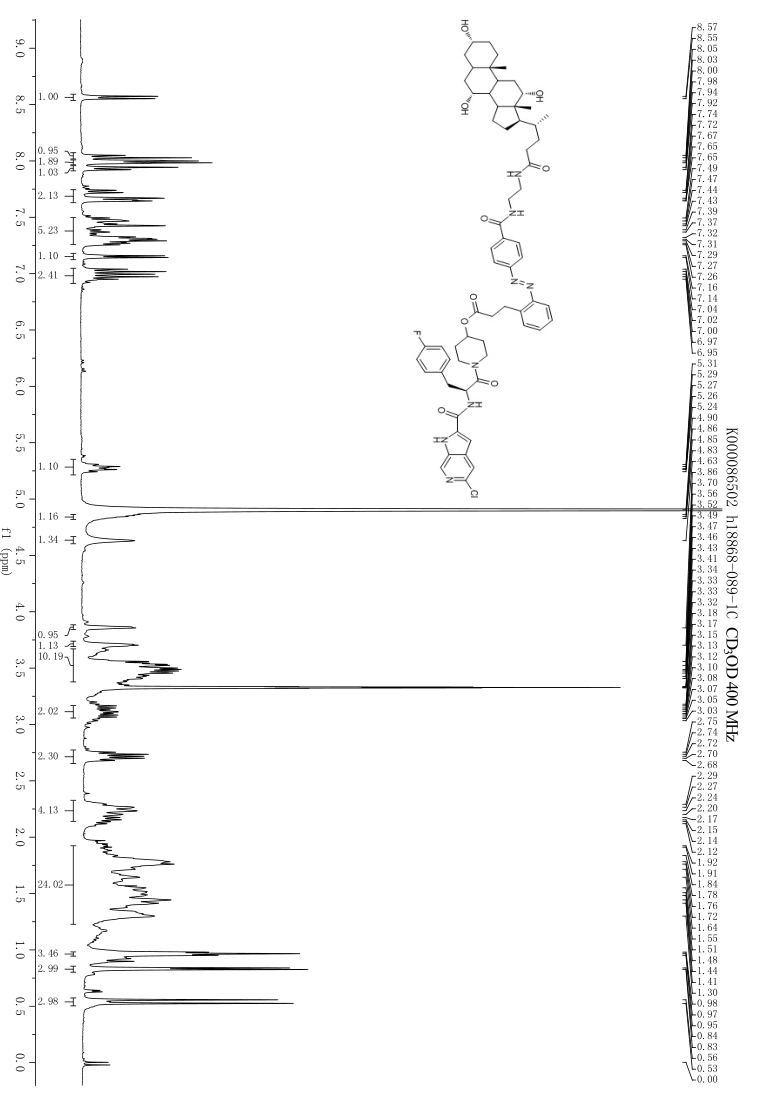
Copy of ^1^H NMR of 9

#
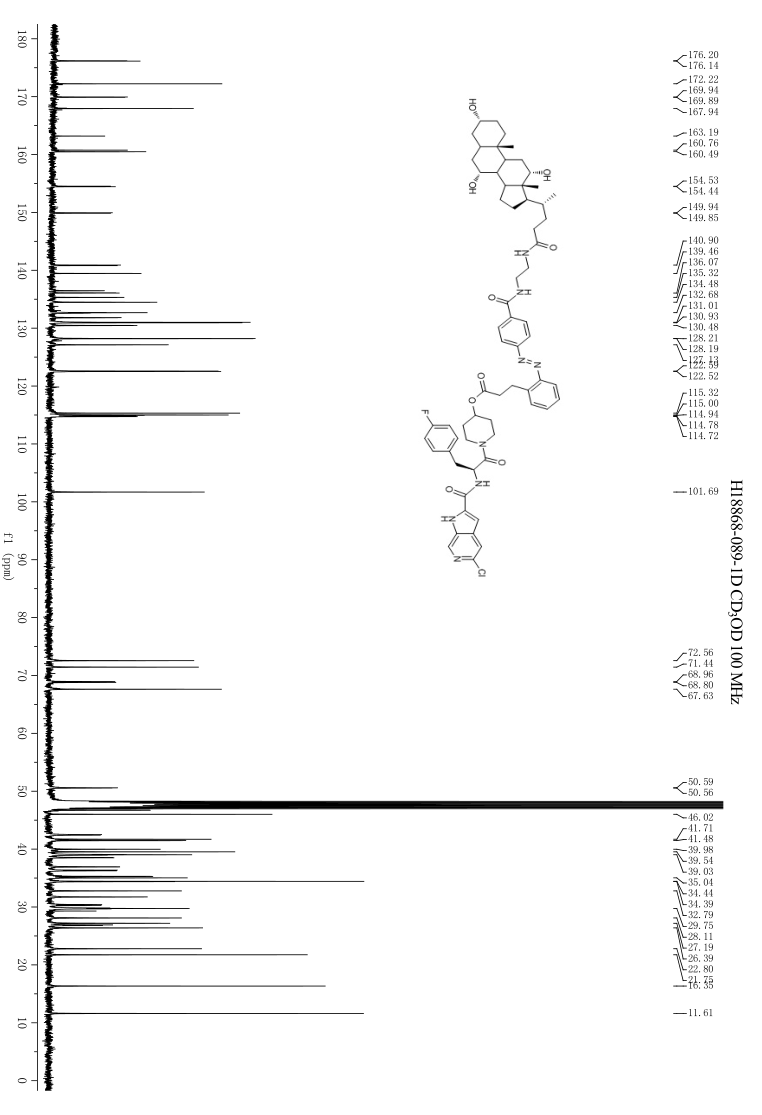
Copy of ^13^C NMR of 9

# Copy of LC-MS chromatogram for 6
